# Negative cooperativity in the formation of two H-bonds with an oxygen H-bond acceptor

**DOI:** 10.1039/d6sc01693f

**Published:** 2026-03-26

**Authors:** Maria Cristina Misuraca, Christopher A. Hunter

**Affiliations:** a Yusuf Hamied Department of Chemistry, University of Cambridge Lensfield Road Cambridge CB2 1EW UK herchelsmith.orgchem@ch.cam.ac.uk

## Abstract

Cooperative effects in H-bond networks can be quantified by measuring the effect of an intramolecular H-bond on the association constant for formation of a second intermolecular H-bond with the same functional group. This approach has been used to quantify the cooperativity associated with the interaction of a phosphoryl oxygen with two H-bond donors. A series of compounds that have an intramolecular H-bond between a phosphinamide oxygen and a phenol hydroxyl group were prepared, using substituents on the phenol to vary the strength of the intramolecular H-bond. The presence of the intramolecular interaction was confirmed by NMR spectroscopy in *n*-octane solution, and titrations were used to measure the association constants for formation of an intermolecular H-bond with perfluoro-*t*-butanol in *n*-octane. Electron-withdrawing substituents on the phenol, which increase the strength of the intramolecular H-bond, were found to decrease the strength of the intermolecular H-bond between the phosphoryl oxygen and perfluoro-*t*-butanol. The results were used to determine the H-bond acceptor parameters for the phosphinamides, *β*, and there is a linear relationship between the values of *β* and the H-bond donor parameter of the phenol involved in the intramolecular H-bond, *α*. The slope of this relationship was used to determine the cooperativity parameter (*κ* = −0.82), which quantifies the negative allosteric cooperativity between the two H-bonding interactions. Polarisation models for cooperativity in H-bond networks would predict positive cooperativity for this system, due to an increase in the polarity of the phosphorus–oxygen bond. The observation of substantial negative cooperativity suggests that the effects observed are due to secondary electrostatic interactions between the two H-bond donors that make through-space repulsive interactions with one another.

## Abstract 2

When a phosphoryl oxygen interacts with two H-bond donors, the strength of the second H-bonding interaction much weaker than the first interaction, due to electrostatic repulsion between the two donors.

## Graphical abstract

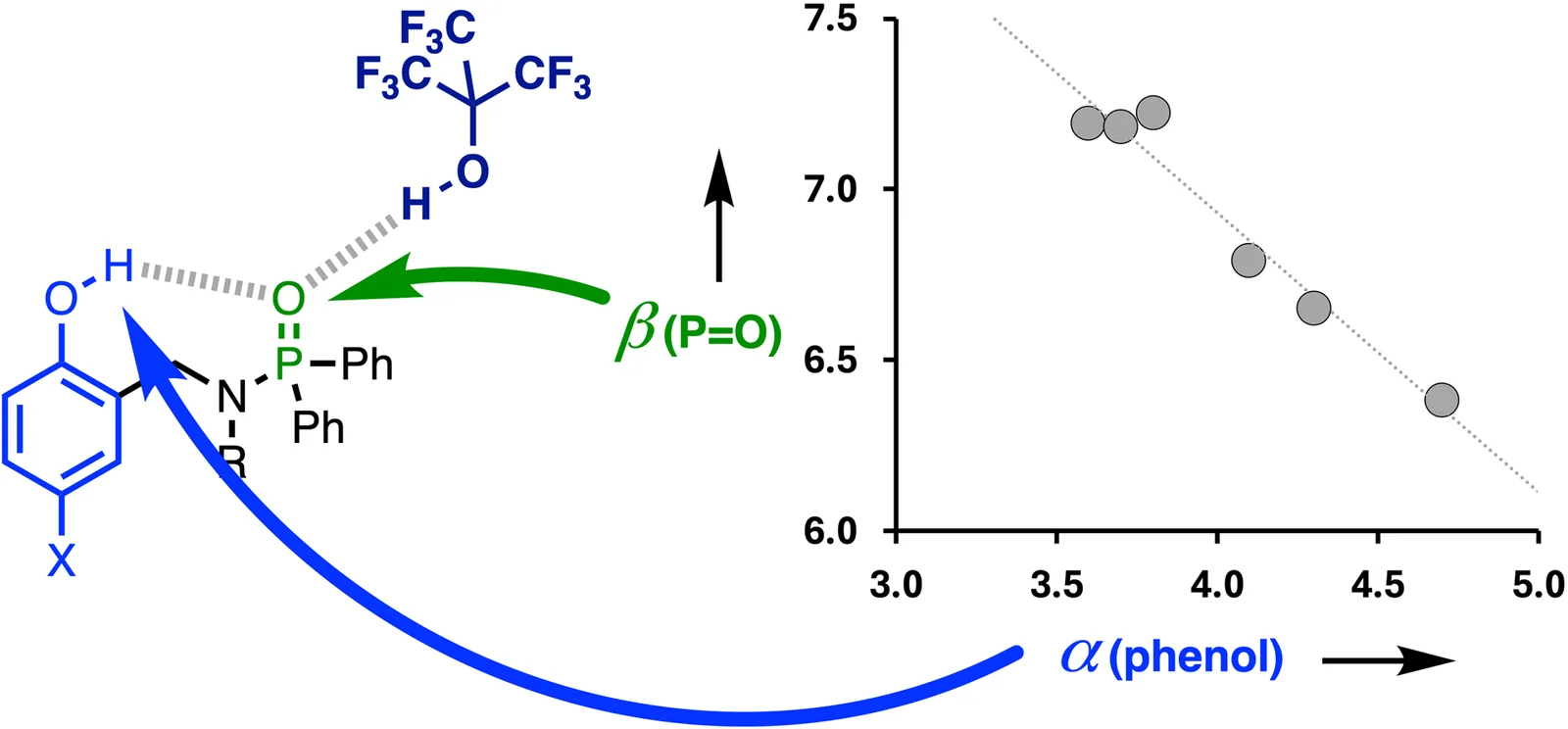

## Introduction

H-bonding is one of the most important non-covalent interactions and plays a major role in determining the relationship between chemical structure and functional properties in natural and synthetic systems, from biomolecules to materials.^1–16^ Although quantitative predictions of the thermodynamic properties of pairwise H-bonding interactions can be achieved using parameters that describe the properties of the two interacting molecules in isolation,^17^ the cooperativity present in H-bond networks adds a layer of complexity. When more than one H-bond can be formed with the same functional group, the first binding event can dramatically modify the interaction strength at the second binding site.^18^ Quantitative prediction of the thermodynamic properties of molecular ensembles that contain H-bond networks therefore remains a challenge for theory.

**Fig. 1:**
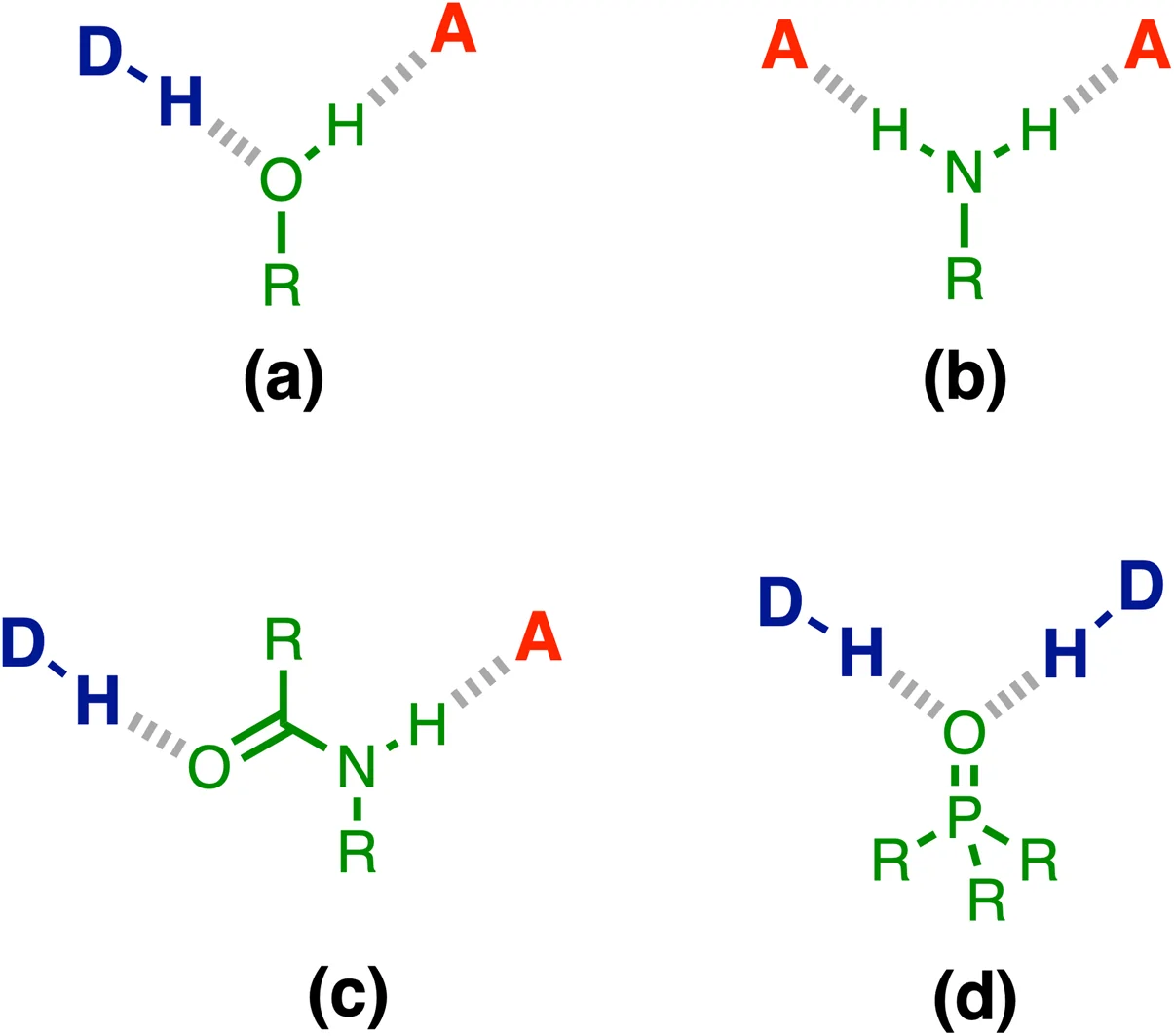
Cooperative H-bond networks. (a) Interaction of an alcohol with a H-bond donor (DH) and a H-bond acceptor (A). (b) Interaction of a primary aniline with two H-bond acceptors. (c) Interaction of a secondary amide with a H-bond donor and a H-bond acceptor. (d) Interaction of a phosphoryl group with two H-bond donors.

Experimental measurements on simple supramolecular model systems provide an ideal platform to collect quantitative data for benchmarking theoretical models. Fig. 1 illustrates examples of three-component networks where a central functional group forms two H-bonds. Alcohols (Fig. 1a) and secondary amides (Fig. 1c) can interact with a H-bond donor (D) and a H-bond acceptor (A), and positive cooperativity is observed between the two interactions.^19–22^ Primary anilines (Fig. 1b) can form a doubly H-bonded complex with two acceptors, and in this case, negative cooperativity is observed.^23^

Here we investigate cooperativity in the H-bonding properties of the phosphoryl group (Fig. 1d), which can form a doubly H-bonded complex with two donors.

**Fig. 2:**
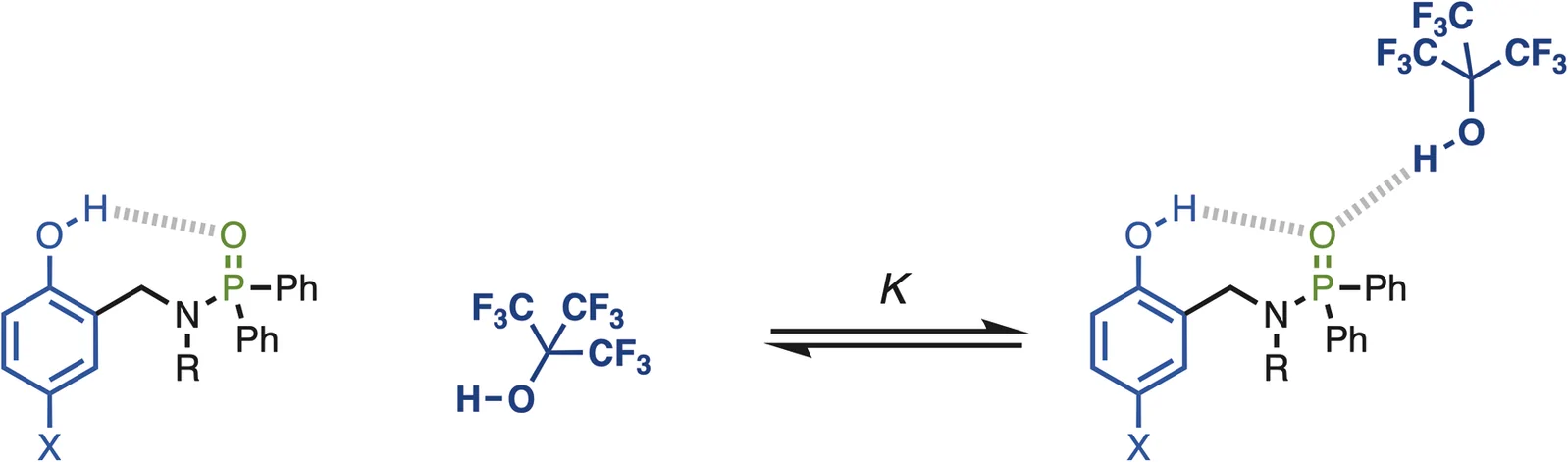
Interaction of a H-bonded phosphoryl group (green) with perfluoro-*t*-butanol (dark blue). X is a substituent that modulates the H-bond donor properties of the phenol intramolecular H-bond donor (light blue).

The approach is illustrated in Fig. 2. The strength of the intramolecular H-bond between the phenol hydroxyl group and phosphoryl oxygen H-bond acceptor can be varied by changing the X substituent. The effect of the intramolecular H-bond on the strength of the intermolecular H-bond formed with a good H-bond donor (perfluoro-*t*-butanol, PFTB) can be quantified by measuring the association constant for formation of the 1 : 1 complex (*K*). The methylene spacer ensures that through-bond electronic communication between the phenol and phosphinamide groups is minimised, so that any substituent effects on the association constant are transmitted by the intramolecular H-bond.

## Results

### Synthesis

**Fig. 3:**
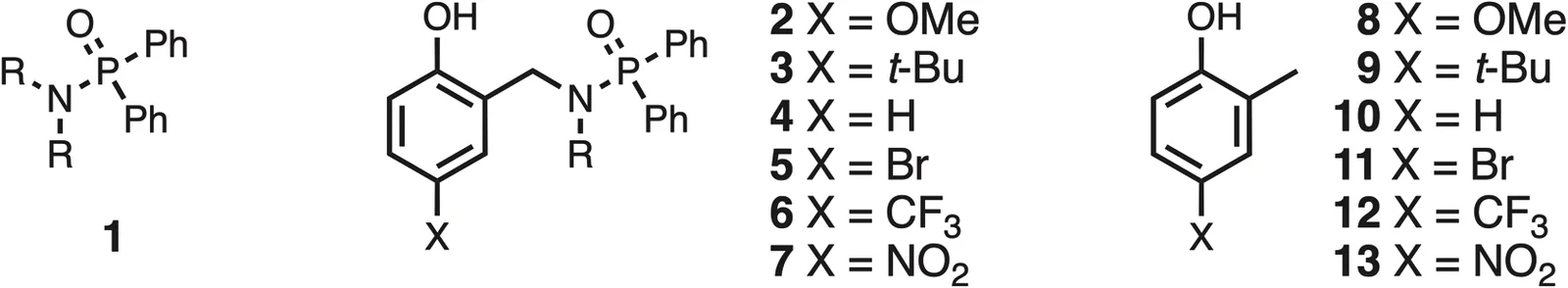
Chemical structure of phosphinamides 1–7 and reference phenols 8–13. R = *n*-hexyl.

The experiment in Fig. 2 requires a set of phosphinamides equipped with different phenol derivatives. Phosphinamides 2–7 in Fig. 3 each have a phenol group that could make an intramolecular H-bond with the phosphoryl group. Compound 1 was used as a reference phosphinamide that cannot make an intramolecular H-bond, and compounds 8–13 were used as reference phenols that cannot make an intramolecular H-bond.

**Scheme 1:**
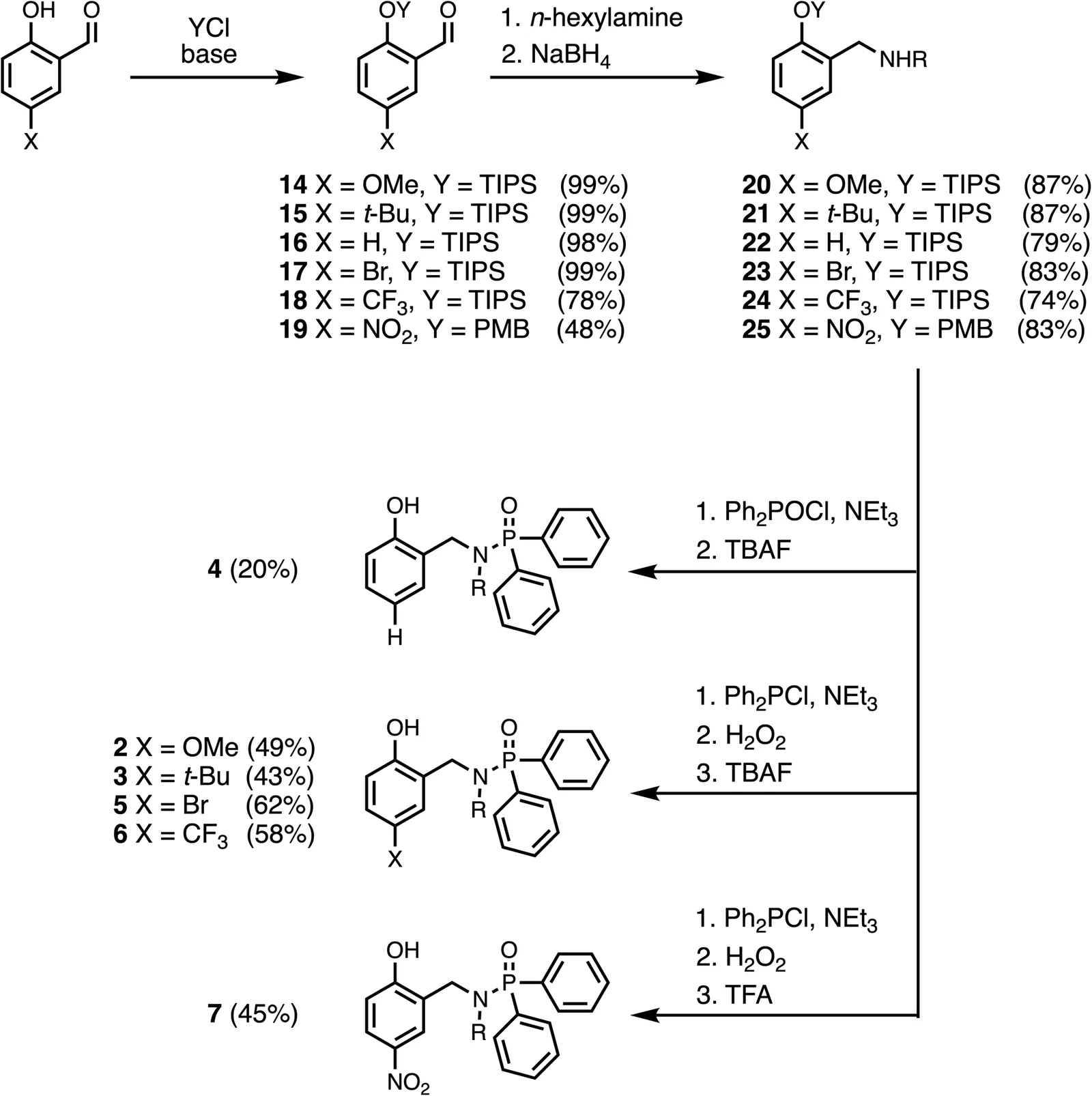
Synthesis of compounds 2–7. R = *n*-hexyl.

Scheme 1 shows the synthetic route to phosphinamides 2–7. The hydroxyl group of the corresponding 2-hydroxybenzaldehyde derivative (14–19) was first protected with either a tri-*iso*-propylsilane (TIPS) or a *p*-methoxybenzyl (PMB) group, and then reductive amination with *n*-hexylamine was used to obtain the secondary amine (20–25). Coupling the secondary amine either with chlorodiphenylphosphine, followed by oxidation with hydrogen peroxide, or with chlorodiphenylphosphine oxide gave the corresponding phosphinamide, and deprotection of the phenol group gave compounds 2–7. Similarly, phosphinamide 1 was synthesised by coupling di-*n*-hexylamine with chlorodiphenylphosphine oxide (see SI for details). Compounds 8–13 were commercially available.

### Intramolecular H-bonding

Table 1 shows ^1^H and ^31^P NMR chemical shifts measured for compounds 1–13 in deuterochloroform. For compounds 2–7, the signal due to the phenol OH group is shifted downfield by more than 5 ppm compared with the corresponding reference phenol 8–13 with the same X substituent (Δ*δ*_OH_). Similarly, the ^31^P NMR signal for compounds 2–7 is shifted downfield by more than 6 ppm compared with the reference phosphinamide 1 (Δ*δ*_P_). These large differences in chemical shift are characteristic of formation of an intramolecular H-bond between the phenol OH group and the phosphoryl oxygen, as shown in Fig. 2.^19–21^ Moreover, the magnitude of the difference in chemical shift depends on the X substituent. The largest chemical shift differences were observed for the most electron-withdrawing X substituents, and the values correlate with the H-bond donor parameter (*α*) of the corresponding reference phenol (*R*^2^ = 0.96 for Δ*δ*_OH_, and 0.99 for Δ*δ*_P_, see SI). These observations indicate that the properties of the intramolecular H-bond in compounds 2–7 depend on the H-bond donor properties of the phenol OH group.

**Table 1:** ^1^H and ^31^P NMR chemical shifts (ppm) measured in deuterochloroform at 298 K compared with the phenol H-bond donor parameter *α*

X	Phenol			Phosphinamide				
	Compound	*α* a	*δ*_OH_/ppm	Compound	*δ*_OH_/ppm	*δ*_P_/ppm	Δ*δ*_OΗ_/ppm^c^	Δ*δ*_P_/ppm^d^
—	—	—	—	1	—	30.6	—	—
OMe	8	3.7	4.5	2	9.7	36.7	5.2	6.1
*t*-Bu	9	3.6	4.6	3	9.9	36.8	5.3	6.2
H	10	3.8	4.7	4	10.0	37.1	5.3	6.5
Br	11	4.1	4.7	5	10.4	37.8	5.7	7.2
CF_3_	12	4.3	5.0 b	6	11.0	38.4	5.8	7.8
NO_2_	13	4.7	5.5	7	11.8	39.4	6.3	8.8

a Value from ref. 24.

b Value from ref. 25.

c Difference between the *δ*_OH_ values measured for compounds 2–7 and the corresponding phenol with the same X substituent 8–13.

d Difference between the *δ*_P_ values measured for compounds 2–7 and compound 1.

### Intermolecular H-bonding

**Fig. 4:**
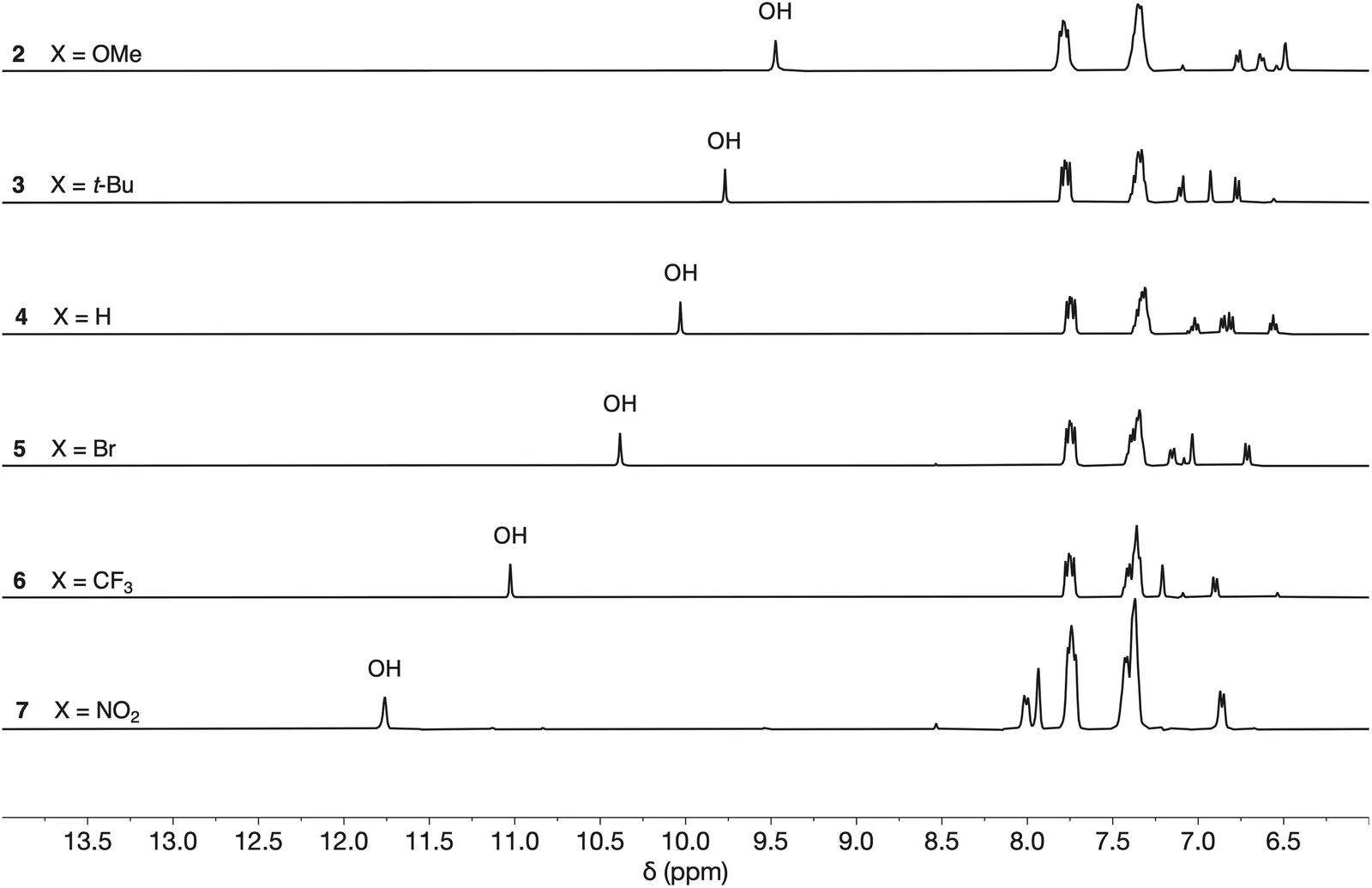
^1^H NMR spectra of compounds 2–7 (0.5 mM) in *n*-octane at 298 K recorded with WET solvent suppression.

The interaction of phosphinamides 1–7 with PFTB was investigated by NMR titrations in *n*-octane solution. NMR dilution experiments showed no evidence of self-association of these compounds at millimolar concentrations in *n*-octane (see SI). Fig. 4 shows the ^1^H NMR spectra of compounds 2–7 recorded in *n*-octane, and the chemical shifts of the signal due to the phenol OH proton are practically identical to those measured in deuterochloroform solution (see Table 1). Very large differences between the ^31^P NMR chemical shift of compound 1 and the other phosphinamides were also observed in *n*-octane solution (see SI), which confirms that the intramolecular H-bond between the phenol OH and the phosphoryl oxygen observed in deuterochloroform is also present in *n*-octane.

**Fig. 5:**
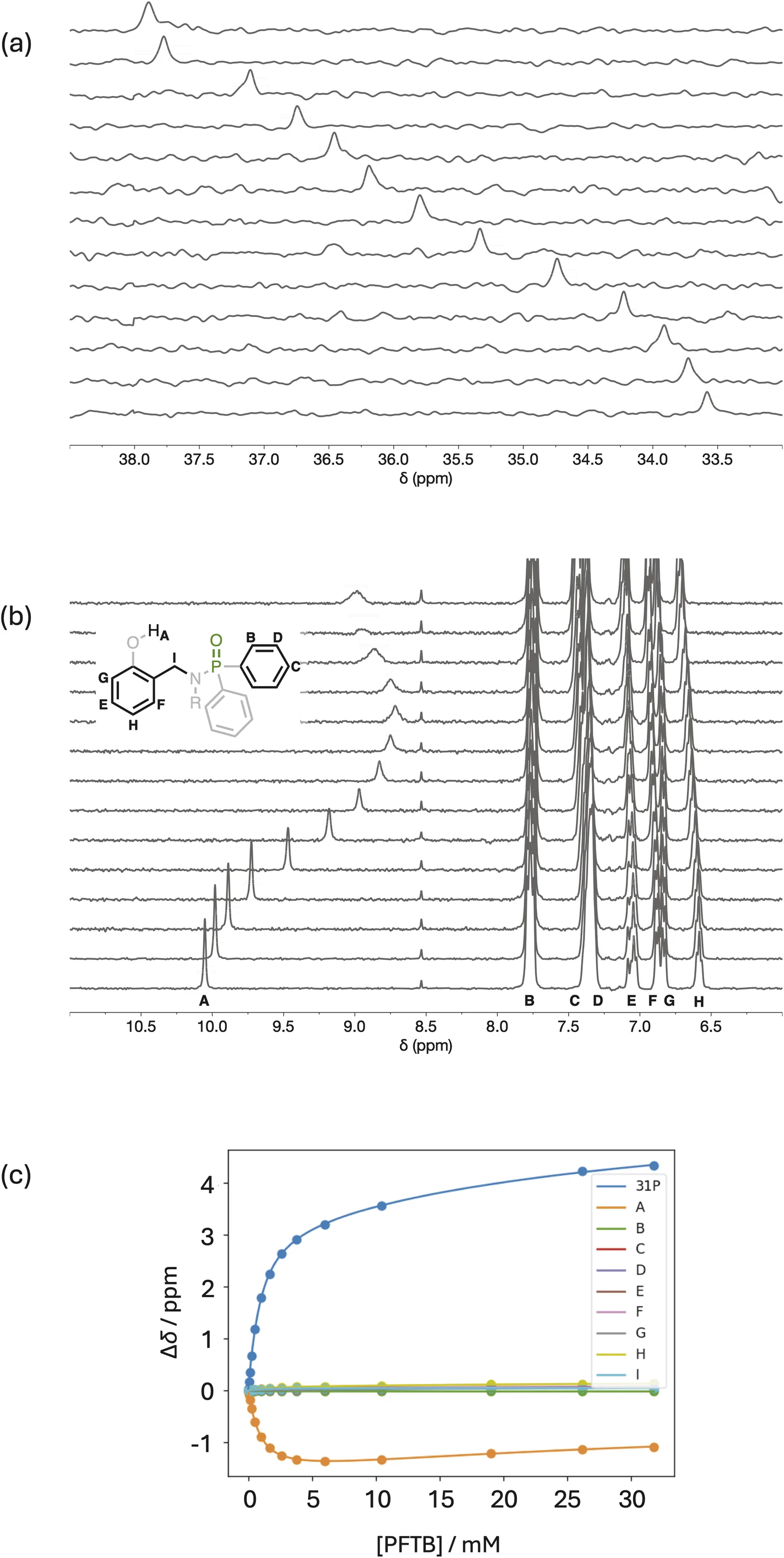
NMR titration data for addition of PFTB to 4 (0.5 mM) in *n*-octane at 298 K. (a) Partial 162 MHz ^31^P NMR spectra with increasing concentration of PFTB bottom to top. (b) Partial 400 MHz ^1^H NMR spectra recorded with WET solvent suppression and increasing concentration of PFTB bottom to top (the proton labelling scheme is shown). The signal at 8.5 ppm is due to an impurity in the solvent. (c) Best fit of the titration data to a 2 : 1 binding isotherm (*K*_1_ = 1590 M^−1^, *K*_2_ = 26 M^−1^).^26^

NMR titration experiments were carried out with phosphinamides as the host and PFTB as the guest in *n*-octane at 298 K. Fig. 5 shows the data for titration of PFTB into 4 (see SI for the other phosphinamides). Addition of PFTB led to a large increase in the chemical shift of the ^31^P signal (Fig. 5a), which is characteristic of formation of an intermolecular H-bond between the PFTB OH group and the phosphoryl oxygen. The largest change in ^1^H NMR chemical was observed for the signal due to the phenol OH, which moved more than 1 ppm upfield on addition of PFTB (Fig. 5b). This decrease in chemical shift is consistent with a decrease in the strength of the intramolecular H-bond in the 1 : 1 complex formed with PFTB. When a large excess of PFTB was added, the signal due to the phenol OH proton started to increase in chemical shift, and this change in direction is indicative of a weak second binding event. The titration data fit well to a 2 : 1 binding isotherm with a high affinity first binding event (*K*_1_ = 1590 M^−1^) and low affinity second binding event (*K*_2_ = 26 M^−1^) (Fig. 5c).^26^

Similar behaviour was observed for the other phosphinamides, and the titration data fit well to a 2 : 1 binding isotherm in all cases (see SI for details). The association constants for formation of the 1 : 1 complexes (*K*_1_) are summarised in Table 2, and the stepwise association constant for formation of the 2 : 1 complex (*K*_2_) was less than 40 M^−1^ in all cases. The association constant for formation of the 1·PFTB complex was too large to be determined accurately, but the NMR titration data can be used to place a lower limit of 10^4^ M^−1^ on the value (see SI). In other words, the association constants in Table 2 measured for the phosphinamides that have an intramolecular H-bond are at least an order of magnitude lower than the value for the reference phosphinamide that does not have an intramolecular H-bond. This result suggests that there is strong negative cooperativity between the two H-bonds made with the oxygen of the phosphoryl group in the PFTB complexes of compounds 2–7 (see Fig. 2). The substituent effects on the values of *K*_1_ listed in Table 2 support this conclusion. Electron-withdrawing X groups, which increase the H-bond donor parameter of the phenol OH group (*α* values in Table 1) and increase the strength of the intramolecular H-bond, lead to a decrease in the strength of the intermolecular H-bond, *i.e.* lower values of *K*_1_.

**Table 2:** Association constants (*K*_1_/M^−1^) for formation of 1 : 1 complexes with perfluoro-*t*-butanol measured by NMR titrations in *n*-octane at 298 K and H-bond acceptor parameters (*β*) calculated using eqn (1)^a^

X	Compound	*K*_1_/M^−1^	*β*
—	1	—	10.7 b
OMe	2	1640 ± 260	7.2
*t*-Bu	3	1650 ± 440	7.2
H	4	1730 ± 170	7.2
Br	5	910 ± 170	6.8
CF_3_	6	730 ± 60	6.6
NO_2_	7	490 ± 150	6.4

a Each titration was repeated at least three times, and the average values are reported with errors at the 95% confidence limit.

b H-bond acceptor parameter determined by UV-vis titration of compound 1 into 4-phenyl azophenol in *n*-octane at 298 K (see SI for details).

**Fig. 6:**
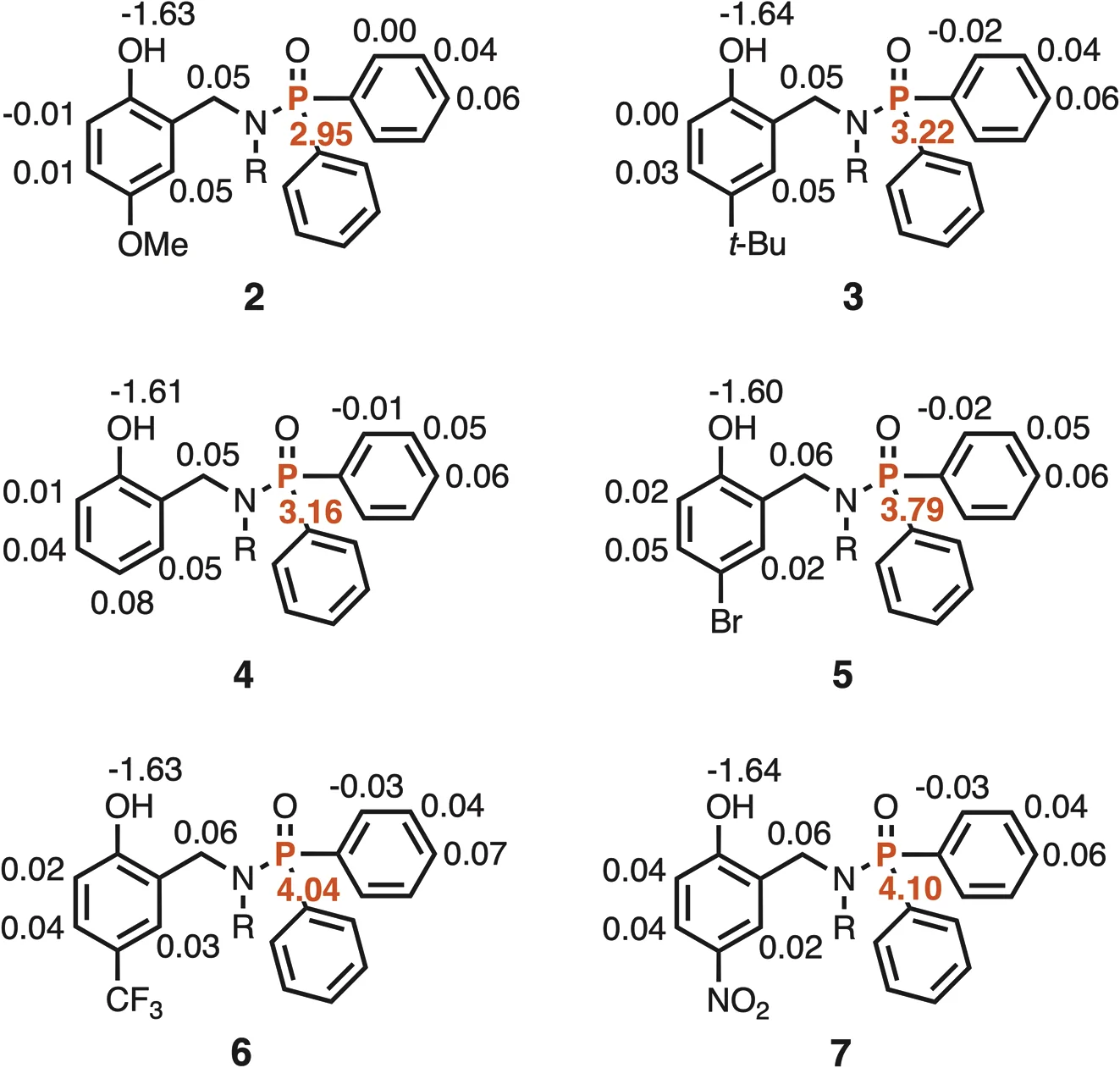
Limiting complexation-induced changes in chemical shift (Δ*δ*/ppm) for formation of 1 : 1 complexes between phosphinamides 2–7 and PFTB in *n*-octane. ^1^H NMR signals shown in black, and ^31^P NMR signals shown in red.

The limiting complexation-induced changes in chemical shift for formation of the 1 : 1 complexes with PFTB are summarized in Fig. 6. In all cases, there is a 3–5 ppm increase in the chemical shift of the ^31^P signal, which is indicative of formation of an intermolecular H-bond between the phosphoryl oxygen and the OH group of PFTB. There is a consistent decrease of 1.6 ppm in the chemical shift of the signal due to the phenol OH group in the 1 : 1 complexes, which suggests that the intermolecular H-bond competes with the intramolecular H-bond to some extent. However, it is clear that the intramolecular H-bond is intact in the 1 : 1 complexes, because the chemical shifts of the signals due to the phenol OH groups in the 1 : 1 complexes are 3–5 ppm higher than the chemical shifts observed for the reference phenols that do not make intramolecular H-bonds. These observations are consistent with negative cooperativity that leads to mutual weakening of the two H-bonds in the 1 : 1 complex.

**Fig. 7:**
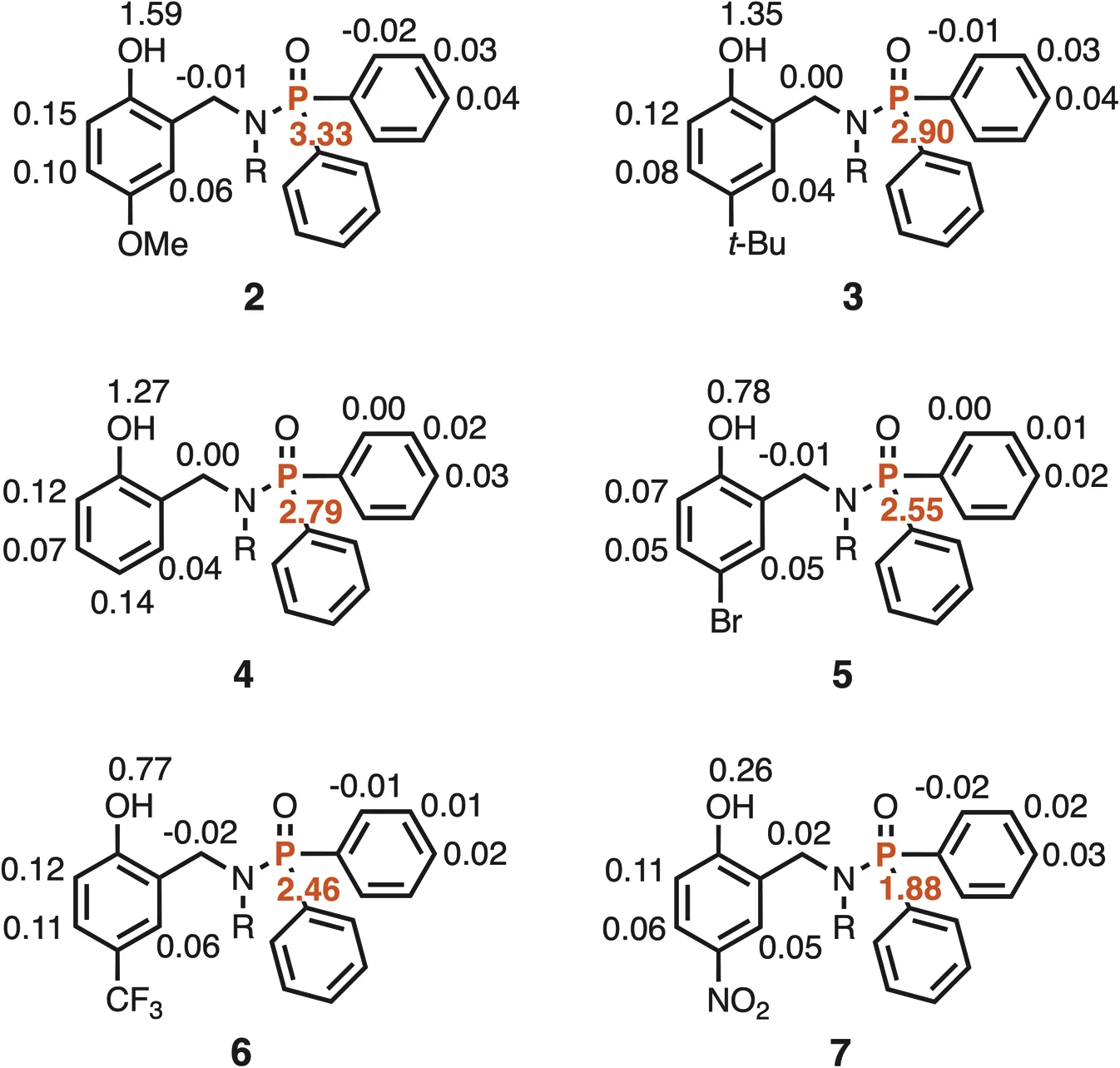
Limiting complexation-induced changes in chemical shift (Δ*δ*/ppm) for formation of 1 : 2 complexes between phosphinamides 2–7 and PFTB in *n*-octane. ^1^H NMR signals shown in black, and ^31^P NMR signals shown in red.

**Fig. 8:**
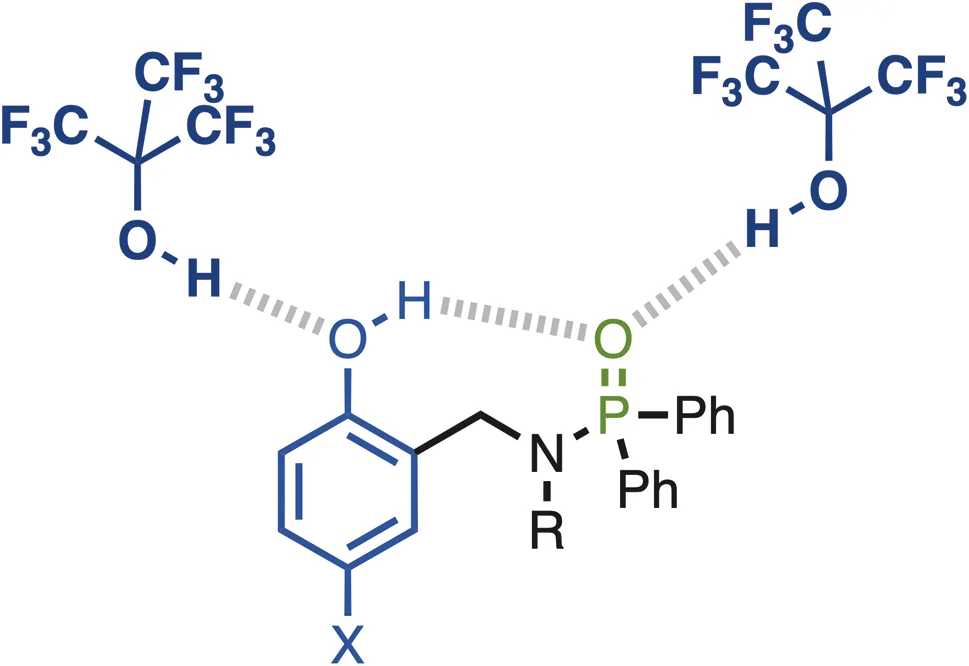
Possible structure of the 2 : 1 complexes formed with PFTB in *n*-octane.

The 2 : 1 complexes are less well-characterised, because saturation is not reached in the binding isotherm (less than 50% bound for the second binding event in most cases). Nevertheless the limiting complexation-induced changes in chemical shift for formation of the 2 : 1 complexes with PFTB can be used to deduce something about the site of the second binding interaction (Fig. 7). There is an increase of 0.3–1.6 ppm the chemical shift of the signal due to the OH group and an increase of 1.9–3.3 ppm the chemical shift of the ^31^P signal on binding the second equivalent of PFTB. These observations suggest the second binding event leads to an increase in the strength of the intramolecular H-bond in the 2 : 1 complex compared with the 1 : 1 complex. The second largest change in ^1^H NMR chemical shift is observed for the signal due to the CH proton *ortho* to the OH group (+0.1 ppm), which suggests that the second PFTB forms a H-bond with the oxygen acceptor of the phenol OH group (Fig. 8). As we have shown previously, such interactions between hydroxyl groups increase the polarity of the phenol H-bond donor, which would lead to an increase in the strength of the intramolecular H-bond with the phosphoryl group.^19–21^

The cooperative effect of the intramolecular interaction on the H-bond properties of the phosphoryl oxygen can be quantified by converting the association constants in Table 2 to H-bond acceptor parameters, *β*, using eqn (1).

1 Δ*G*°/kJ mol^−1^ = −*RT* ln *K*_1_ = −(*α* − *α*_S_)(*β* − *β*_S_) + 6

where *α*_S_ and *β*_S_ are the H-bond parameters of the solvent (*α*_S_ = 1.2 and *β*_S_ = 0.6), and *α* is the H-bond donor parameter of PFTB (*α* = 4.9).^17,24^

**Fig. 9:**
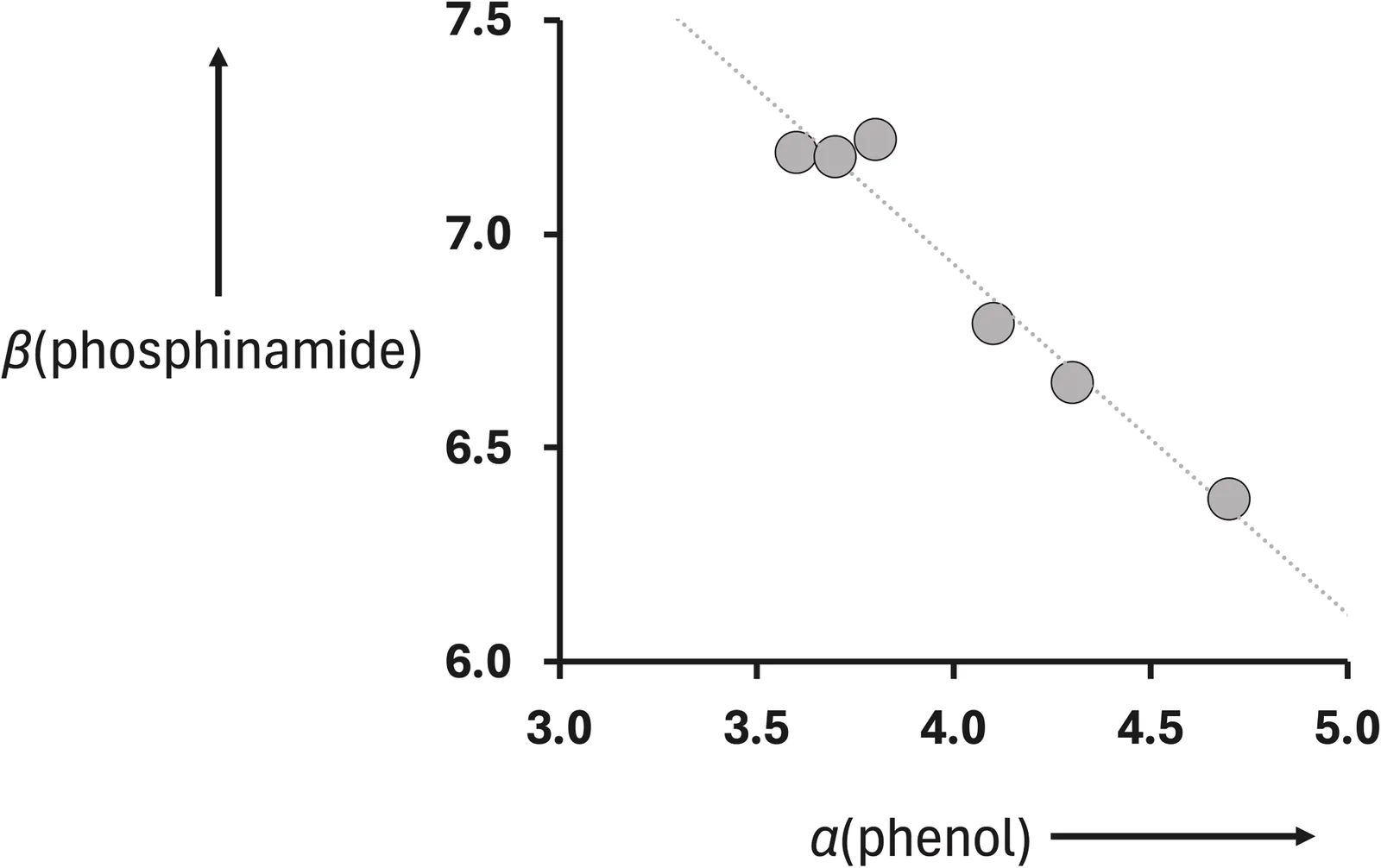
Relationship between the H-bond acceptor parameters of phosphinamides 2–7, *β*(phosphinamide) in Table 2, and the H-bond donor parameters of the corresponding reference phenols 8–13, *α*(phenol) in Table 1. The best fit line is *y* = −0.82*x* + 10.2 (*R*^2^ = 0.96).

Fig. 9 shows that there is a linear relationship between the value of *β* measured for compounds 2–7 (Table 2) and the value of *α* for the corresponding reference phenols 8–13 (Table 1). The slope of the best fit line defines the cooperativity parameter, *κ*, which is −0.82 (eqn (2)).^19^

2 *β* = *β*_0_ + *κα*_D_

where *β*_0_ is the H-bond acceptor parameter of a free phosphinamide, *β* is the H-bond acceptor parameter of a phosphinamide that is H-bonded to a second H-bond donor, which has a H-bond donor parameter *α*_D_, and *κ* is a functional-group-specific parameter that quantifies the sensitivity to cooperative effects.

Although there is a significant extrapolation involved, the intercept of the line of best fit in Fig. 9 is close to the value of *β*_0_ that can be estimated using the value measured for compound 1 (10.7). The cooperativity parameter measured for the phosphoryl oxygen, *κ*, is large and negative. A negative value indicates negative cooperativity, *i.e.* the two H-bonds formed with the phosphoryl oxygen are mutually destabilising. The magnitude of *κ* is large enough to change the H-bond properties of a H-bonded phosphoryl oxygen from one of the best H-bond acceptors with a *β* value at the top of the H-bond acceptor scale to a mid-range H-bond acceptor that resembles a carbonyl group.

## Conclusions

Compounds with an intramolecular H-bond between a phosphinamide oxygen and a phenol hydroxyl group have been used to investigate the effect of cooperativity on the H-bond acceptor properties of the phosphoryl oxygen. Downfield changes in the ^1^H NMR chemical shift of the phenol hydroxyl group and in the ^31^P NMR chemical shift of the phosphoryl group were used to confirm the presence of an intramolecular H-bond in *n*-octane solution. ^1^H and ^31^P NMR titration experiments showed that perfluoro-*t*-butanol (PFTB) binds to the phosphoryl oxygen to form a 1 : 1 complex, in which the phosphoryl oxygen forms an intramolecular H-bond with the phenol H-bond donor and an intermolecular H-bond with the PFTB H-bond donor. The association constants for formation of 1 : 1 complexes were measured for a series of phosphinamides in which the strength of the intramolecular H-bond was varied using polarising substituents on the phenol. Electron-withdrawing substituents, which increase the strength of the intramolecular H-bond, were found to decrease the strength of the intermolecular H-bond between the phosphoryl oxygen and the PFTB.

The results were used to determine the H-bond acceptor parameters for the phosphinamides, *β*, and there is a linear relationship between the values of *β* and the H-bond donor parameter of the phenol involved in the intramolecular H-bond, *α*. The slope of this relationship was used to determine the cooperativity parameter (*κ* = −0.82), which quantifies the negative allosteric cooperativity between the two H-bonding interactions. The magnitude of *κ* for the phosphoryl oxygen is much larger than the values measured previously for the negative cooperativity involved in the interaction of two acceptors with primary anilines (*κ* = −0.10) and the positive cooperativity involved in the interaction of a donor and an acceptor with alcohols and secondary amides (*κ* = +0.33 and +0.20 respectively).^19–22^

**Fig. 10:**
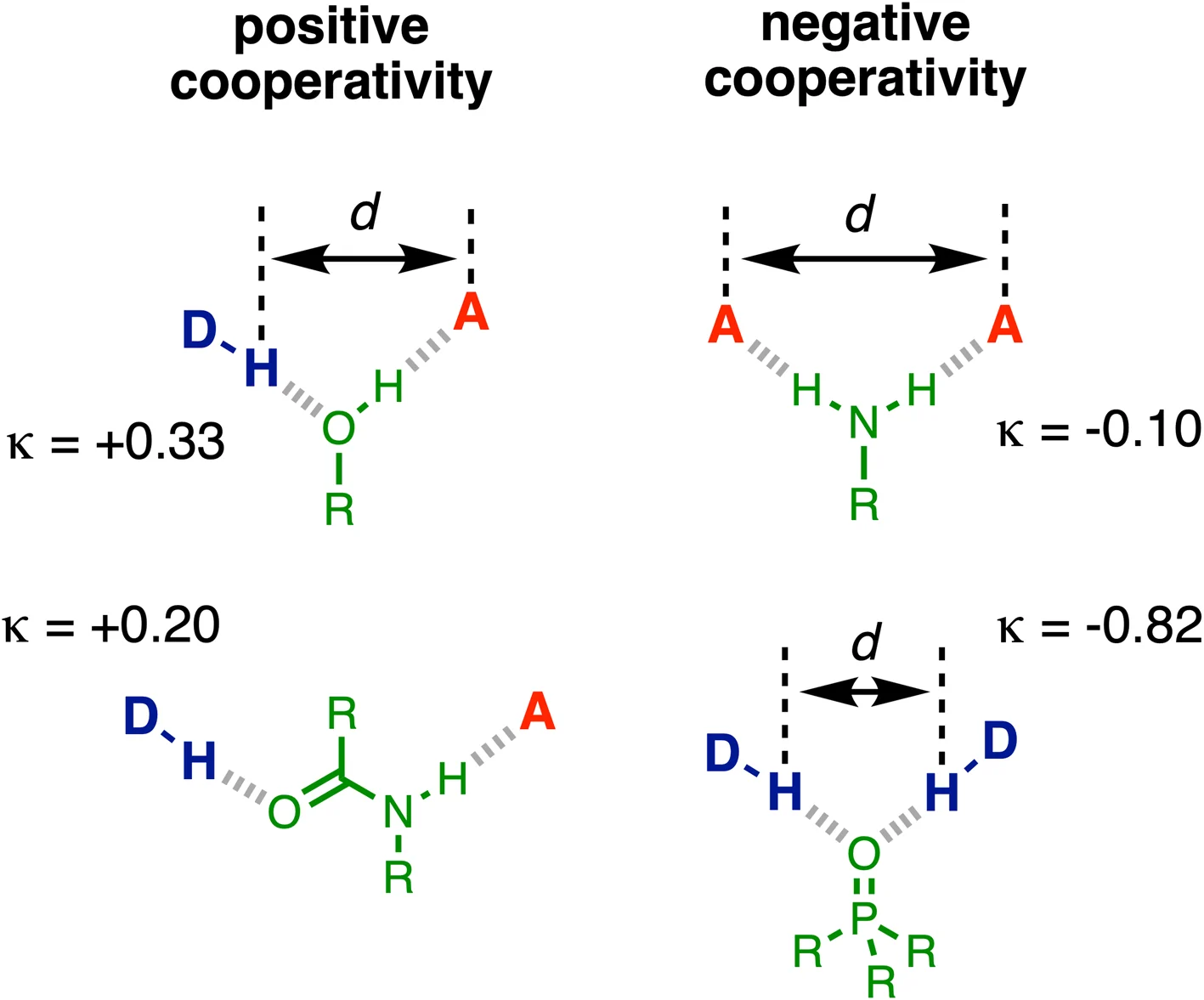
Comparison of the cooperativity parameters *κ* measured for formation of two H-bonding interactions with different functional groups and the distance between the two donor and/or acceptor sites (d).

Cooperative effects are a common feature of H-bonded networks involving multiple interactions, and the measured values of *κ* are summarised in Fig. 10. One interpretation of the positive cooperativity observed in H-bonded networks involving alcohols and secondary amides is that formation of the first H-bond leads to polarisation of the functional group, and this increase in polarity stabilises the second H-bond. The experiments described here show that formation of a H-bond to a phosphoryl oxygen substantially weakens the second H-bond formed by the oxygen with a second H-bond donor. In this case, polarisation of the phosphorus–oxygen bond by the first H-bond would lead to a more negative oxygen and an increase in the strength of the second H-bond. Although polarisation may play a role, it is clear that this is not the major effect operating in this system. A more likely explanation for the observed results is that there is a significant through-space electrostatic repulsion between the two H-bond donors that point towards one another in the doubly H–bonded complex. This effect is likely to play a major role in the cooperativity observed for alcohols and anilines, whereas the donor and acceptor are too far apart to make direct electrostatic interactions in the secondary amide system (Fig. 10). Bond polarisation is likely the most important factor governing cooperativity in secondary amides. For the other functional groups, Fig. 10 shows that there is a correlation between the measured values of *κ* and the distance between the two donor and/or acceptor sites. The large value of *κ* observed for the phosphoryl oxygen probably stems from the close approach of the two H-bond donors, which leads to larger electrostatic interactions than in the other functional groups.

## Author contributions

The manuscript was written through contributions of all authors.

## Conflicts of interest

There are no conflicts to declare.

## Supplementary Material

SC-017-D6SC01693F-s001

## Data Availability

All supporting data is provided in the supplementary information (SI). Supplementary information: synthetic procedures, full characterization including ^1^H and ^13^C NMR spectra of all compounds, NMR and UV-vis absorption dilutions and titrations. See DOI: https://doi.org/10.1039/d6sc01693f.
