# Supplementary material for: Negative cooperativity in the formation of two H-bonds with an oxygen H-bond acceptor
Source: Chem Sci. 2026 Mar 26;17(19):9609–15. doi: 10.1039/d6sc01693f (PMC13019641; doi:10.1039/d6sc01693f)
Supplement: SC-017-D6SC01693F-s001 [file SC-017-D6SC01693F-s001.pdf]

Supplementary Information (SI) for Chemical Science.
This journal is © The Royal Society of Chemistry 2026

SUPPLEMENTARY INFORMATION

NEGATIVE COOPERATIVITY IN THE FORMATION OF TWO H-BONDS WITH AN OXYGEN H-BOND ACCEPTOR

MARIA CRISTINA MISURACA AND CHRISTOPHER A. HUNTER*

YUSUF HAMIED DEPARTMENT OF CHEMISTRY, UNIVERSITY OF CAMBRIDGE, LENSFIELD ROAD, CAMBRIDGE CB2 1EW, UK.
**HERCHELSMITH.ORGCHEM@CH.CAM.AC.UK*

TABLE OF CONTENTS

1. **MATERIALS AND METHODS** **S2**
2. **SYNTHESIS AND CHARACTERIZATION OF COMPOUNDS** **S3**
   2.1 **SYNTHESIS OF 1** **S4**
   2.2 **SYNTHESIS OF 2** **S6**
   2.3 **SYNTHESIS OF 3** **S11**
   2.4 **SYNTHESIS OF 4** **S17**
   2.5 **SYNTHESIS OF 5** **S21**
   2.6 **SYNTHESIS OF 6** **S27**
   2.7 **SYNTHESIS OF 7** **S32**
3. **NMR TITRATIONS IN *N*-OCTANE** **S38**
4. **UV-VIS TITRATIONS IN *N*-OCTANE** **S54**

1. MATERIALS AND METHODS

All commercial reagents were purchased from Sigma-Aldrich, Alfa Aesar, Fisher Scientific, Fluorochem or Acros Organics and were used without any further purification unless specified otherwise. Anhydrous solvents were obtained from a Grubbs PS-MD-5 solvent purification system. Thin layer chromatography was carried out using silica gel 60F (Merck) on glass plates and visualised using UV light (254 or 365 nm) and developed using a potassium permanganate solution. Flash chromatography was performed on an automated system (CombiFlash® Rf⁺ or CombiFlash® Rf⁺ Lumen) using pre-packed cartridges of silica (50 µm PuriFlash® columns). Analytical HPLC was performed on an Agilent HP-1100 Series HPLC system, using XSelect® CSH C18 (2.5 um, 4.6 x 50 mm) column. Preparative HPLC was performed on an Agilent HP-1100 Series HPLC system, using XSelect® CSH C18 (5 um, 19 x 150 mm) column.

^{1}H-NMR, ^{13}C-NMR and ^{31}P NMR characterization spectra were recorded on various Bruker spectrometers (400 MHz AVIII 400, 400 MHz Neo 400, 700 MHz AVIII 700 Cryo) using the residual solvent as the internal standard. ^{1}H-NMR and ^{31}P NMR titration spectra in n-octane were recorded in a Bruker 400 MHz AVIII 400 spectrometer using a capillary with d_6-DMSO as internal standard. All chemical shifts (*δ*) are quoted in ppm and coupling constants given in Hz.

UPLC analysis of samples was performed using Waters Acquity H-class UPLC coupled with a single quadrupole Waters SQD2. Three different columns were used: Acquity UPLC CSH C18 column (130 Å, 1.7 µm, 2.1 mm x 50 mm), Acquity UPLC BEH C8 column (130 Å, 1.7 µm, 2.1 mm x 50 mm) and Acquity UPLC BEH C4 column (300 Å, 1.7 µm, 2.1 mm x 50 mm).

HRMS analysis was performed in an Agilent 1100 pump and autosampler with a Waters LCT Premier TOF (Time of Flight) mass spectrometer.

2. SYNTHESIS AND CHARACTERIZATION OF COMPOUNDS.

a)

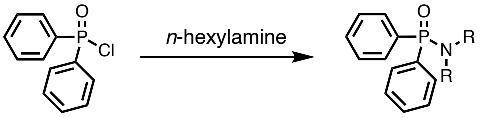

1 (50%)

b)

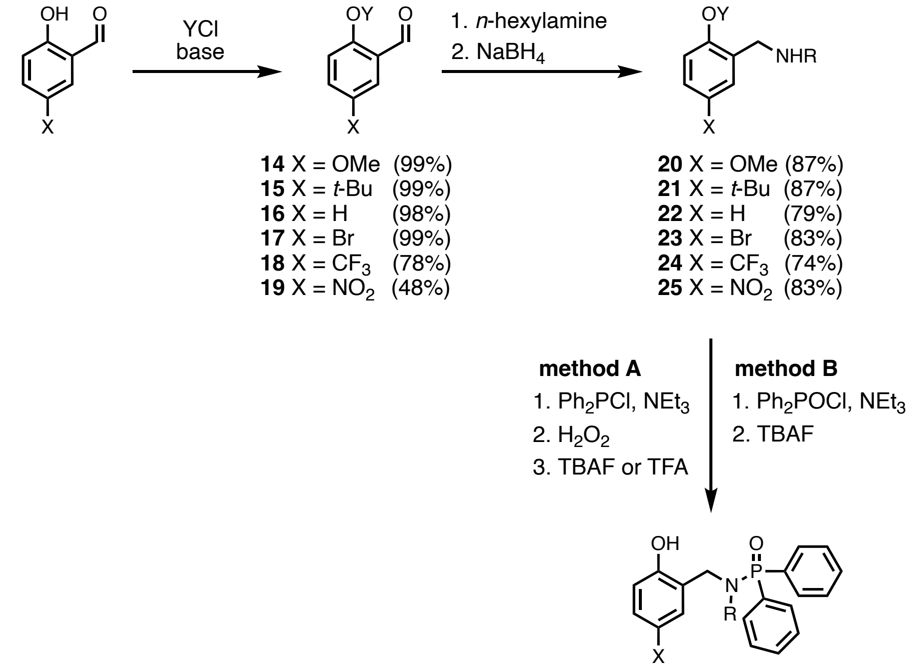

14 X = OMe (99%)
15 X = *t*-Bu (99%)
16 X = H (98%)
17 X = Br (99%)
18 X = CF_3 (78%)
19 X = NO_2 (48%)

20 X = OMe (87%)
21 X = *t*-Bu (87%)
22 X = H (79%)
23 X = Br (83%)
24 X = CF_3 (74%)
25 X = NO_2 (83%)

2 X = OMe (49%)
3 X = *t*-Bu (43%)
4 X = H (20%)
5 X = Br (62%)
6 X = CF_3 (58%)
7 X = NO_2 (45%)

Figure S1. General scheme for the synthesis of a) compound **1**; b) compounds **2 - 7**. Y = TIPS or PMB; base = Et_3N; R = *n*-hexyl.

2.1 SYNTHESIS OF 1.

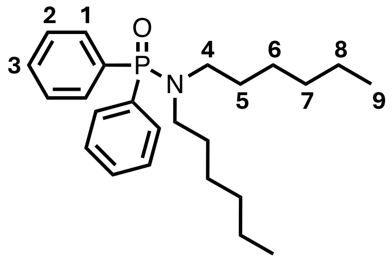

Chlorodiphenylphosphine oxide (0.4 ml, 2.10 mmol) was added dropwise to a solution of dihexylamine (1.00 ml, 4.29 mmol) in dry tetrahydrofuran (30 ml). The reaction mixture was allowed to stir at room temperature under inert atmosphere for 18 hours. After evaporating tetrahydrofuran, the residue was redissolved in ethyl acetate and the organic layer was washed with brine and dried over Na_2SO_4 anhydrous. The solution was then concentrated under reduced pressure the pure product was isolated as a white solid by silica flash chromatography using a mixture of dichloromethane and methanol: yield 0.40 g (50%).

^{1}H NMR (500 MHz, $CDCl_3$)**:** δ_H = 7.84 (m, 4H, H_{C1}), 7.48 (m, 2H, H_{C3}), 7.42 (m, 4H, H_{C2}), 2.93 (m, 4H, H_{C4}), 1.52 (m, 4H, H_{C5}), 1.26-1.04 (m, 12H, $H_{C6-C7-C8}$), 0.81 (t, 6H, *J* = 7.2 Hz, H_{C9}).

^{13}C NMR (126 MHz, $CDCl_3$)**:** δ_C = 132.59 (d, *J* = 128.7 Hz, C-P), 132.53 (d, *J* = 9.2 Hz, C1), 131.67 (d, *J* = 2.7 Hz, C3), 128.49 (d, *J* = 12.4 Hz, C2), 45.89 (d, *J* = 3.2 Hz, C4), 31.51 (C7), 28.73 (d, *J* = 3.7 Hz, C5), 26.71 (C6), 22.62 (C8), 14.09 (C9).

^{31}P NMR (203 MHz, $CDCl_3$)**:** δ_P = 30.61.

HRMS (ESI$^+$)**:** calculated for $[C_{24}H_{37}NOP]^+$ 386.2542, found $[M+H]^+$ 386.2608; calculated for $[C_{24}H_{36}NOPNa]^+$ 408.2542, found $[M+Na]^+$ 408.2466.

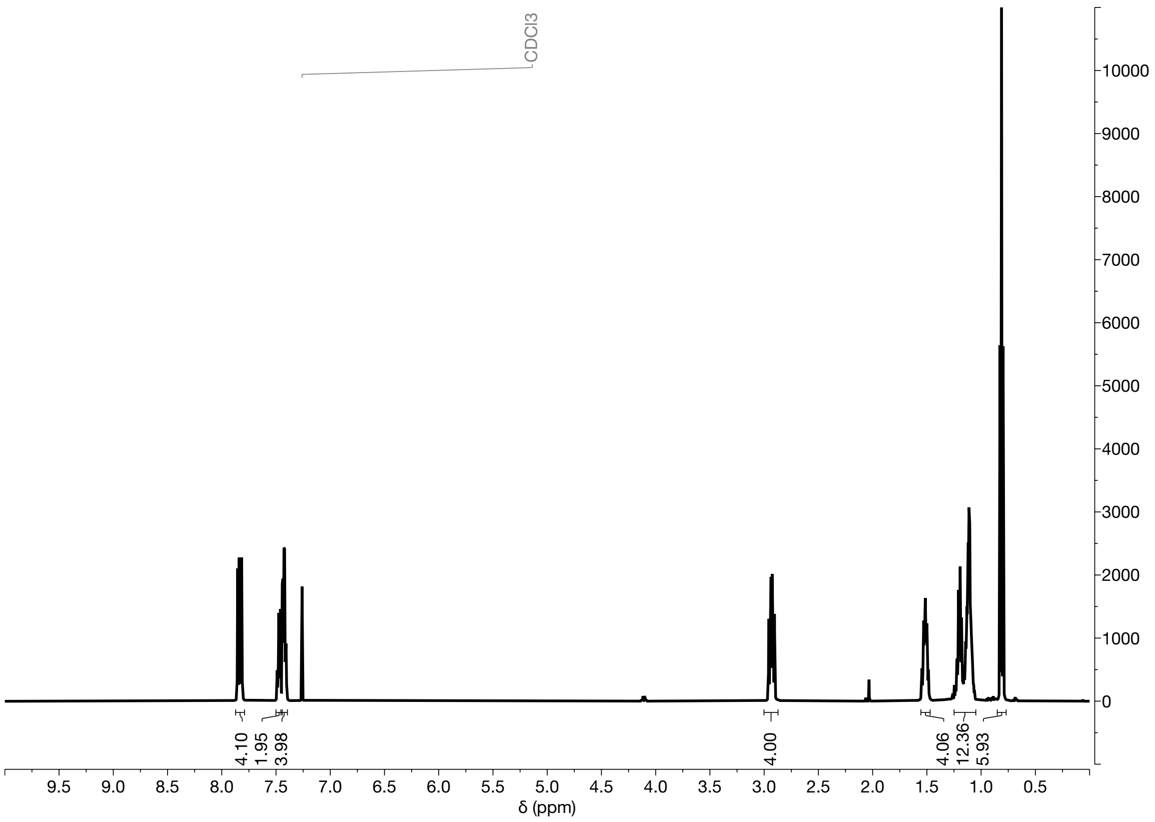

Figure S2. ^{1}H NMR (500 MHz, $CDCl_3$) of compound **1**.

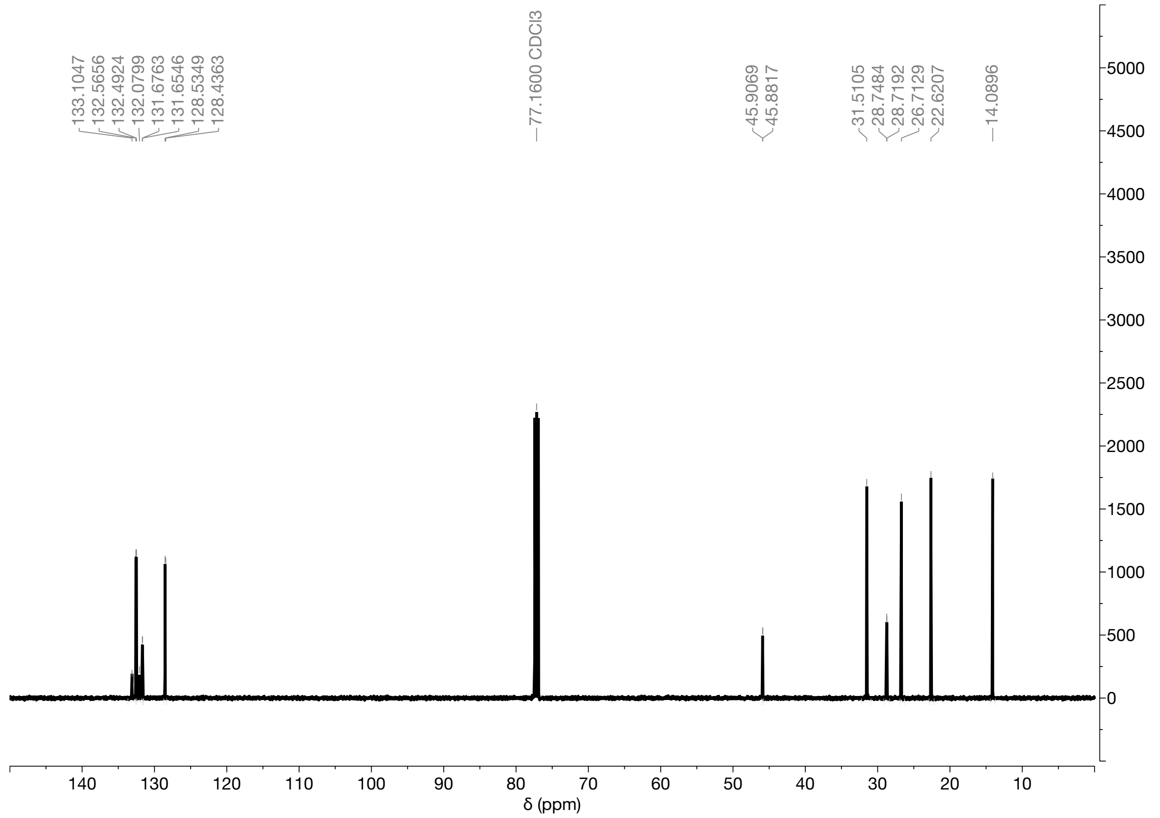

Figure S3. ^{13}C NMR (126 MHz, $CDCl_3$) of compound **1**.

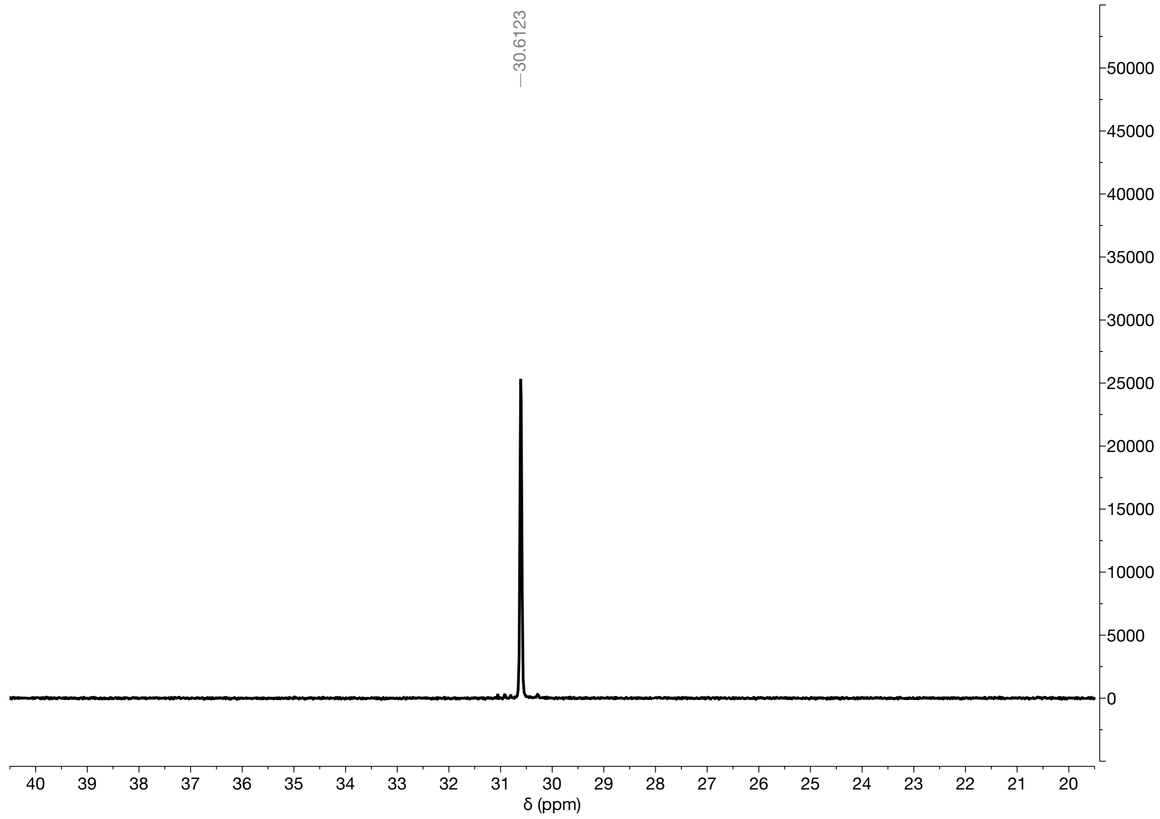

Figure S4. ^{31}P NMR (203 MHz, $CDCl_3$) of compound **1**.

2.2 SYNTHESIS OF 2.

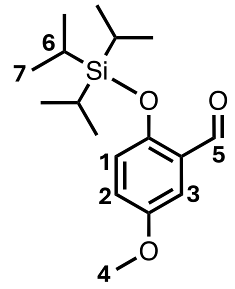

[14] Imidazole (2.10 g, 30.85 mmol) and TIPSCl (4.00 ml, 18.69 mmol) were added to a solution of 2-hydroxy-5-methoxybenzaldehyde (2.00 ml, 15.70 mmol) in dimethylformamide (30 ml). The reaction mixture was allowed to stir at room temperature for 18 hours. The solution was then acidified with 0.1M HCl (aq.) and extracted with ethyl acetate. The organic layer was washed with 5% LiCl (aq.), brine and dried over Na_2SO_4 anhydrous. The solution was then concentrated under reduced pressure and the pure product was afforded as a yellow oil by silica flash chromatography using a mixture of petroleum ether and ethyl acetate: yield 4.82 g (99%).

^{1}H NMR (400 MHz, $CDCl_3$)**:** δ_H = 10.50 (s, 1H, CHO), 7.27 (d, 1H, J = 3.3 Hz, H_{C3}), 7.04 (dd, 1H, J = 9.0, 3.4 Hz, H_{C2}), 6.83 (d, 1H, J = 9.0 Hz, H_{C1}), 3.79 (s, 3H, H_{C4}), 1.32 (m, 3H, H_{C6}), 1.12 (d, 18H, J = 7.4 Hz, H_{C7}).

^{13}C NMR (101 MHz, $CDCl_3$)**:** δ_C = 190.24 (C5), 154.10 (C-C5), 153.87 (C-OMe), 126.73 (C-OTIPS), 124.10 (C2), 121.13 (C1), 109.57 (C3), 55.87 (C4), 18.09 (C7), 13.11 (C6).

HRMS (ESI$^+$)**:** calculated for $[C_{17}H_{29}O_3Si]^+$ 309.1797, found $[M+H]^+$ 309.1871; calculated for $[C_{17}H_{28}O_3SiNa]^+$ 331.1797, found $[M+Na]^+$ 331.1684.

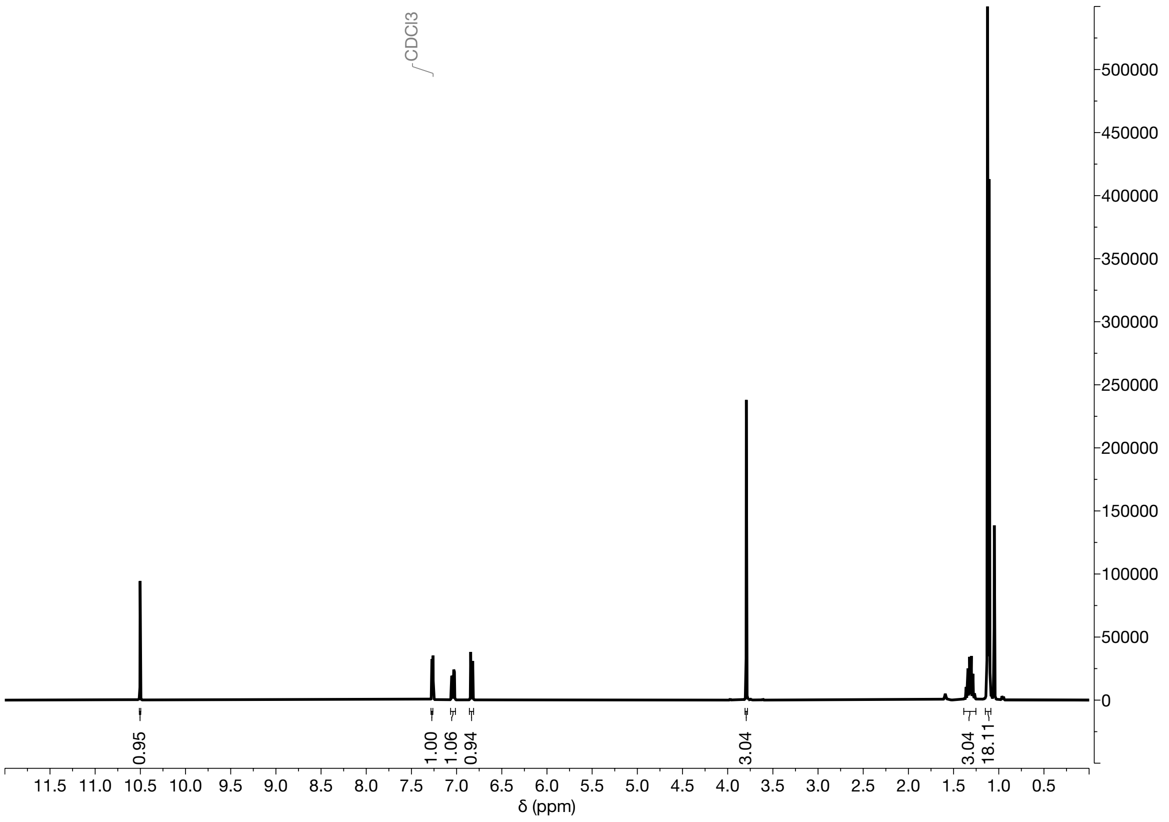

Figure S5. ^{1}H NMR (400 MHz, $CDCl_3$) of compound **14**.

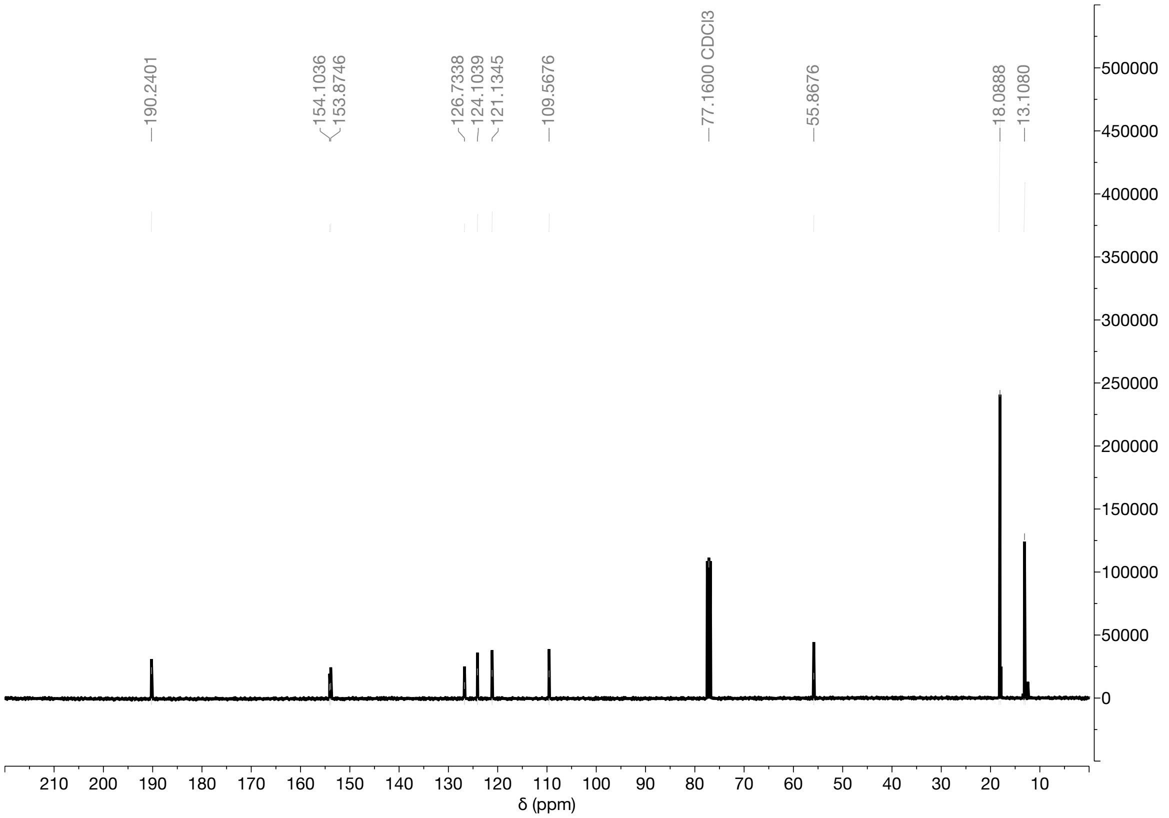

Figure S6. ^{13}C NMR (101 MHz, $CDCl_3$) of compound **14**.

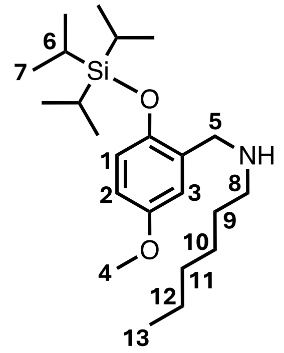

[20] A mixture of **14** (2.04 g, 6.61 mmol) and *n*-hexylamine (1.30 ml, 9.84 mmol) in dry dichloromethane (30 ml) was stirred over molecular sieves at room temperature for 18 hours. The reaction mixture was then cooled to 0 °C and diluted with methanol (30 ml). Sodium borohydride (1.51 g, 39.92 mmol) was added in small aliquots and the reaction was left stirring for 30 minutes. The solution was then quenched with water and filtered. After extracting with ethyl acetate, the organic layer was washed with brine and dried over Na_2SO_4 anhydrous. The solution was then concentrated under reduced pressure and the pure product was afforded as a light-yellow oil by silica flash chromatography using a mixture of dichloromethane and ethyl acetate: yield 2.28 g (87%).

^{1}H NMR (500 MHz, $CDCl_3$): δ_H = 6.85 (d, 1H, J = 3.1 Hz, H_{C3}), 6.71 (d, 1H, J = 8.8 Hz, H_{C1}), 6.64 (dd, 1H, J = 8.8, 3.2 Hz, H_{C2}), 3.78 (s, 2H, H_{C5}), 3.76 (s, 3H, H_{C4}), 2.60 (t, 2H, J = 7.3, H_{C8}), 1.50 (p, 2H, J = 7.1 Hz, H_{C9}), 1.28 (m, 9H, $H_{C10\text{-}12}$, H_{C6}), 1.10 (d, 18H, J = 7.5 Hz, H_{C7}), 0.87 (t, 3H, J = 7.0, H_{C13}).

^{13}C NMR (126 MHz, $CDCl_3$)**:** δ_C = 153.63 (C̲-C5), 148.11 (C̲-OTIPS), 130.93 (C̲-OMe), 118.46 (C1), 115.53 (C3), 112.78 (C2), 55.79 (C4), 49.93 (C5), 49.43 (C8), 31.93 (C12), 30.24 (C9), 27.25 (C10), 22.76 (C11), 18.23 (C7), 14.18 (C13), 13.19 (C6).

HRMS (ESI$^+$)**:** calculated for $[C_{23}H_{44}NO_2Si]^+$ 393.3136, found $[M+H]^+$ 394.3129.

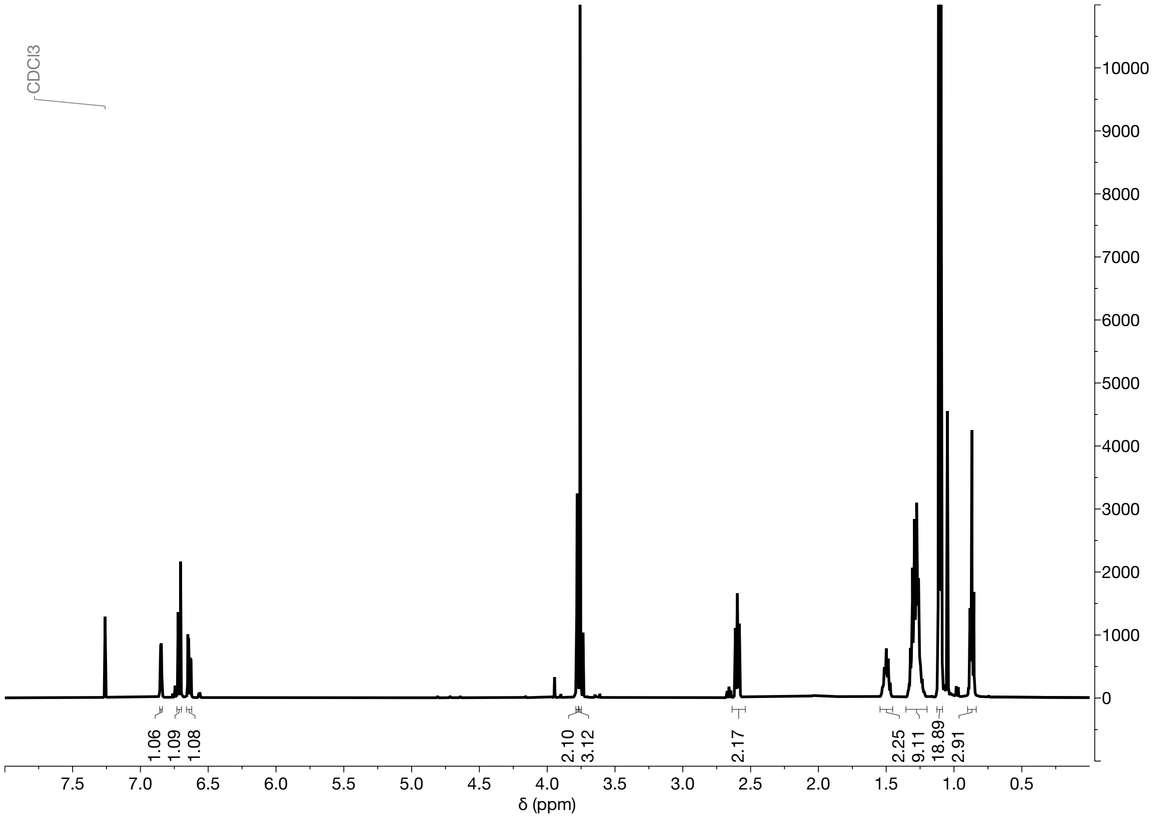

Figure S7. ^{1}H NMR (400 MHz, $CDCl_3$) of compound **20**.

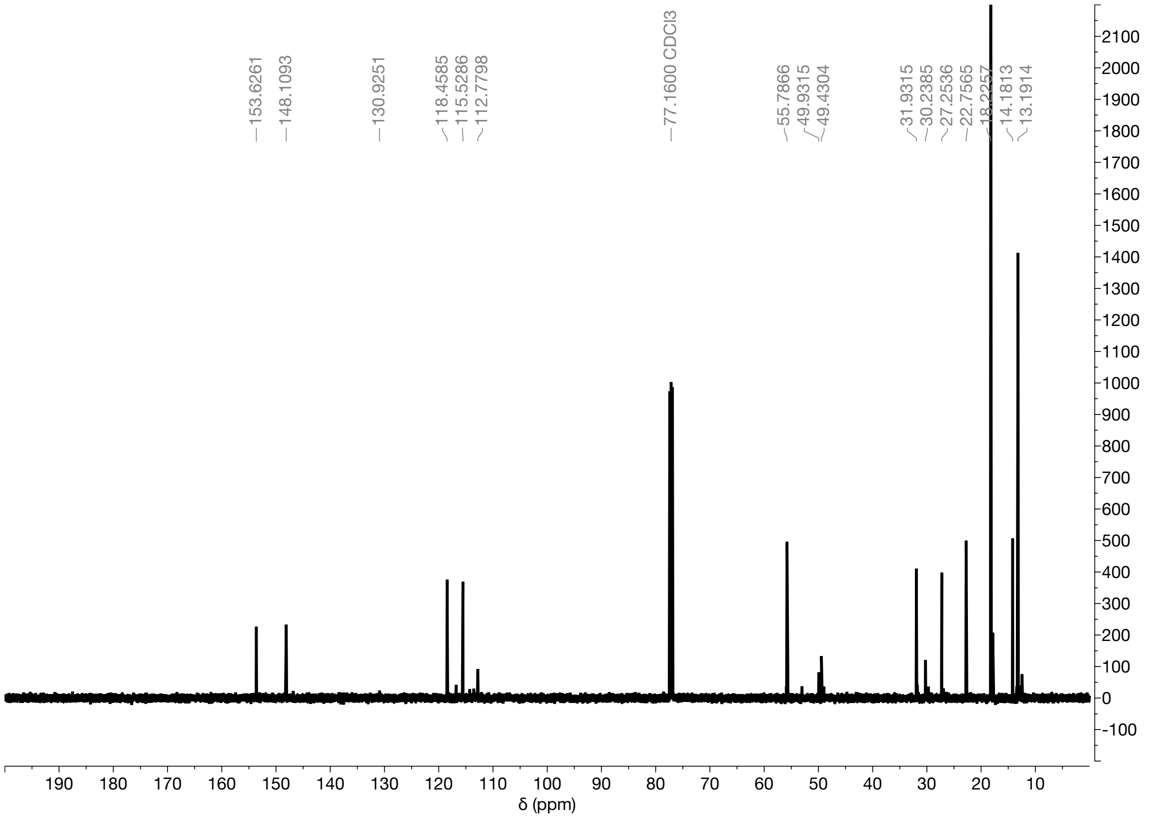

Figure S8. ^{13}C NMR (101 MHz, $CDCl_3$) of compound **20**.

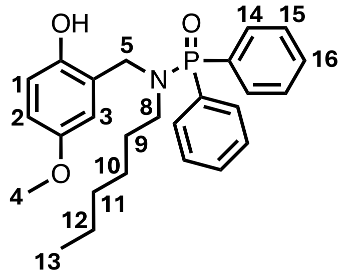

[2] Chlorodiphenylphosphine (0.60 ml, 3.25 mmol) and triethylamine (1.00 ml, 7.17 mmol) were added dropwise to a solution of **20** (1.03 g, 2.62 mmol) in dry tetrahydrofuran (15 ml) at -78°C. The reaction mixture was allowed to stir at room temperature under inert atmosphere for 18 hours. Thereafter, the reaction mixture was cooled to 0 °C and 30% H_2O_2 (aq.) (0.40 ml, 3.91 mmol) was added. The mixture was left stirring for 1 hour before diluting it with iced water. After extracting with dichloromethane, the organic layer was washed with saturated NH_4Cl (aq.) and dried over Na_2SO_4 anhydrous. After concentrating the solution under reduced pressure, 0.20 g of the resulting oil was dissolved in tetrahydrofuran (1 ml) and the solution was cooled to 0 ºC. A 1M solution of TBAF in tetrahydrofuran (0.70 ml, 0.70 mmol) was, then, dropwise added and the mixture was stirred at room temperature for 30 minutes. After adding water (1 ml), the solution was extracted with ethyl acetate. The organic layer was then washed with brine, dried over Na_2SO_4 anhydrous and concentrated under reduced pressure. The final product was purified by preparative HPLC using a XSelect® CSH C18 (5 um, 19 x 150 mm) column with 45% acetonitrile in water: overall yield 0.56 g (49%).

^{1}H NMR (400 MHz, $CDCl_3$): δ_H = 9.66 (s, 1H, O<u>H</u>), 7.79 (m, 4H, H_{C14}), 7.55 (m, 2H, H_{C16}), 7.48 (m, 4H, H_{C15}), 6.92 (d, 1H, J = 8.8 Hz, H_{C1}), 6.79 (dd, 1H, J = 8.8, 3.1 Hz, H_{C2}), 6.63 (d, 1H, J = 3.1 Hz, H_{C3}), 4.21 (d, 2H, J = 10.9 Hz, H_{C5}), 3.74 (s, 3H, H_{C4}), 2.72 (m, 2H, H_{C8}), 1.40 (p, 2H, J = 7.2 Hz, H_{C9}), 1.13 (m, 2H, H_{C12}), 0.99 (m, 4H, $H_{C10\text{-}C11}$), 0.78 (t, 3H, J = 7.2 Hz, H_{C13}).

^{13}C NMR (101 MHz, $CDCl_3$): δ_C = 152.47 (<u>C</u>-OMe), 150.81 (<u>C</u>-OH), 132.47 (d, J = 10.0 Hz, C14), 132.45 (d, J = 2.8 Hz, C16), 130.80 (d, J = 128.7 Hz, <u>C</u>-P), 128.85 (d, J = 12.8 Hz, C15), 123.80 (<u>C</u>-C5), 119.79 (C1), 117.48 (C3), 114.74 (C2), 55.92 (C4), 46.83 (d, J = 4.2 Hz, C5), 45.89 (d, J = 4.3 Hz, C8), 31.30 (C10), 28.39 (d, J = 3.1 Hz, C9), 26.38 (C11), 22.53 (C12), 14.0 (C13).

^{31}P NMR (162 MHz, $CDCl_3$): δ_P = 36.73.

HRMS (ESI$^+$)**:** calculated for $[C_{26}H_{33}NO_3P]^+$ 438.2128, found $[M+H]^+$ 438.2201; calculated for $[C_{26}H_{32}NO_3PNa]^+$ 460.2128, found $[M+Na]^+$460.2018.

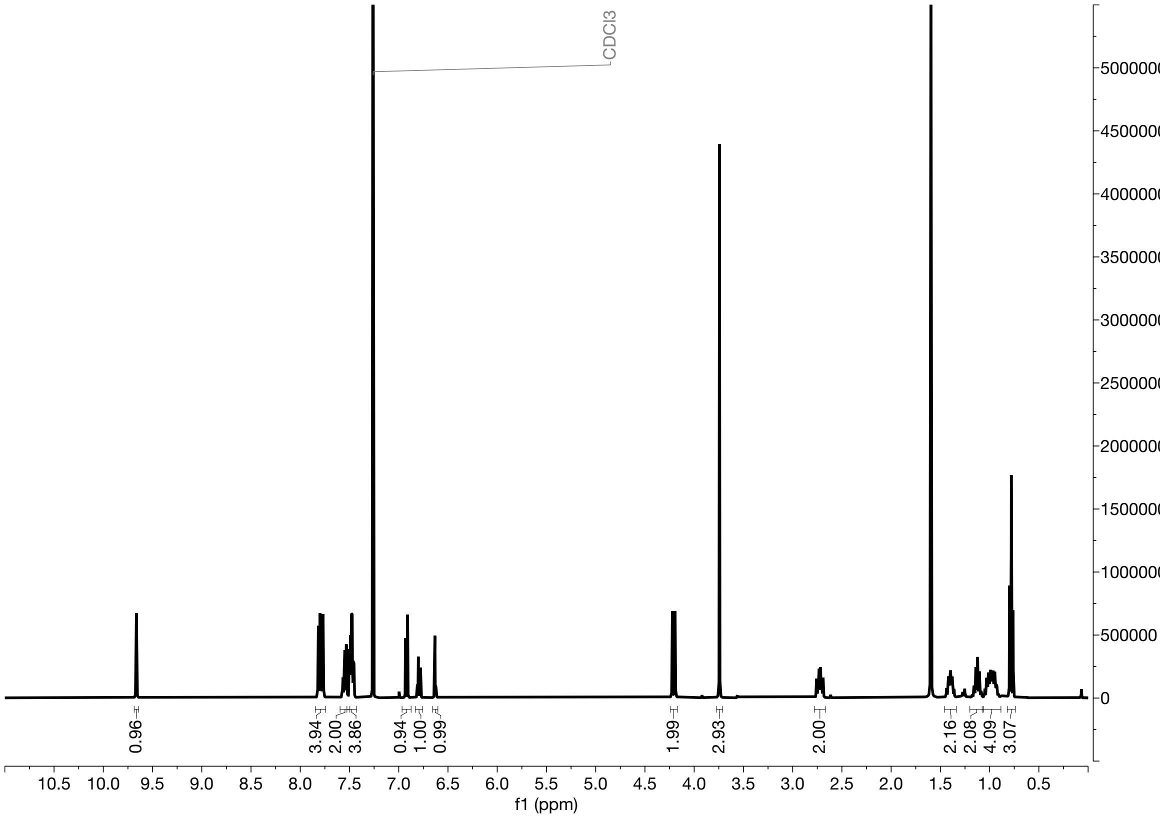

Figure S9. ^{1}H NMR (400 MHz, $CDCl_3$) of compound **2**.

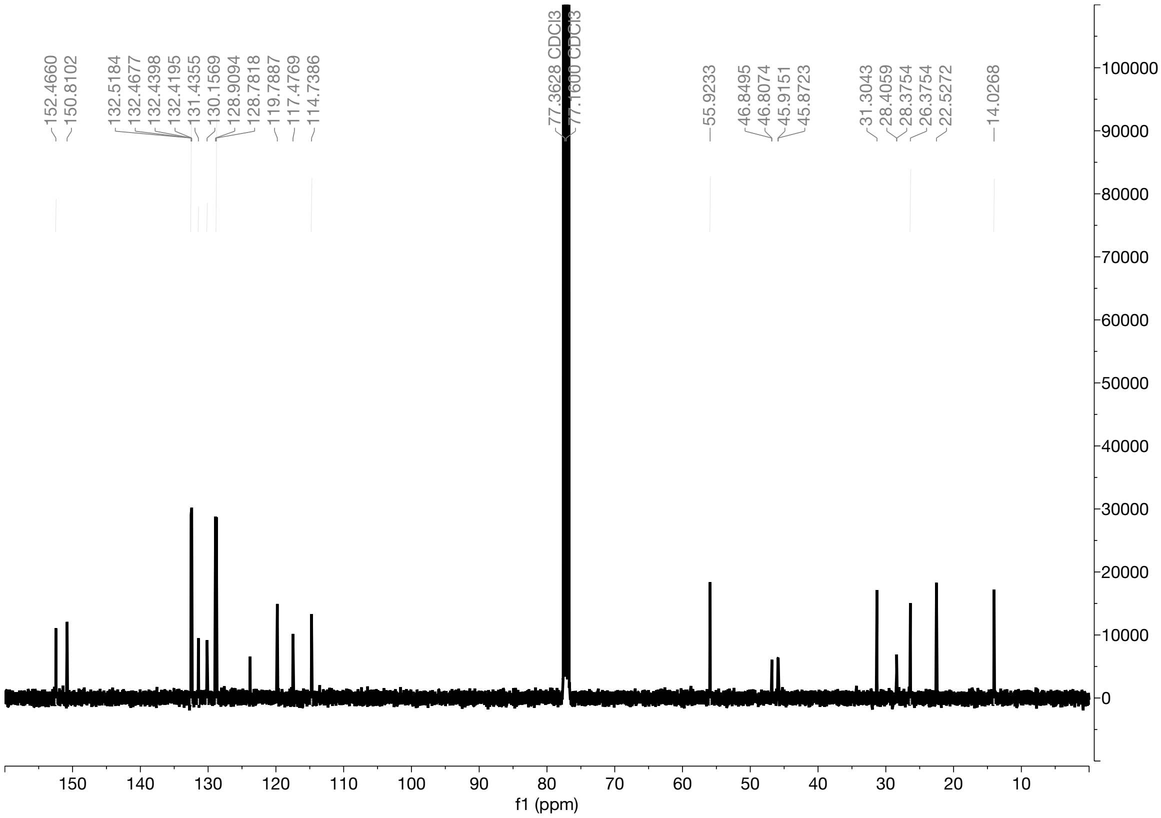

Figure S10. ^{13}C NMR (101 MHz, $CDCl_3$) of compound **2**.

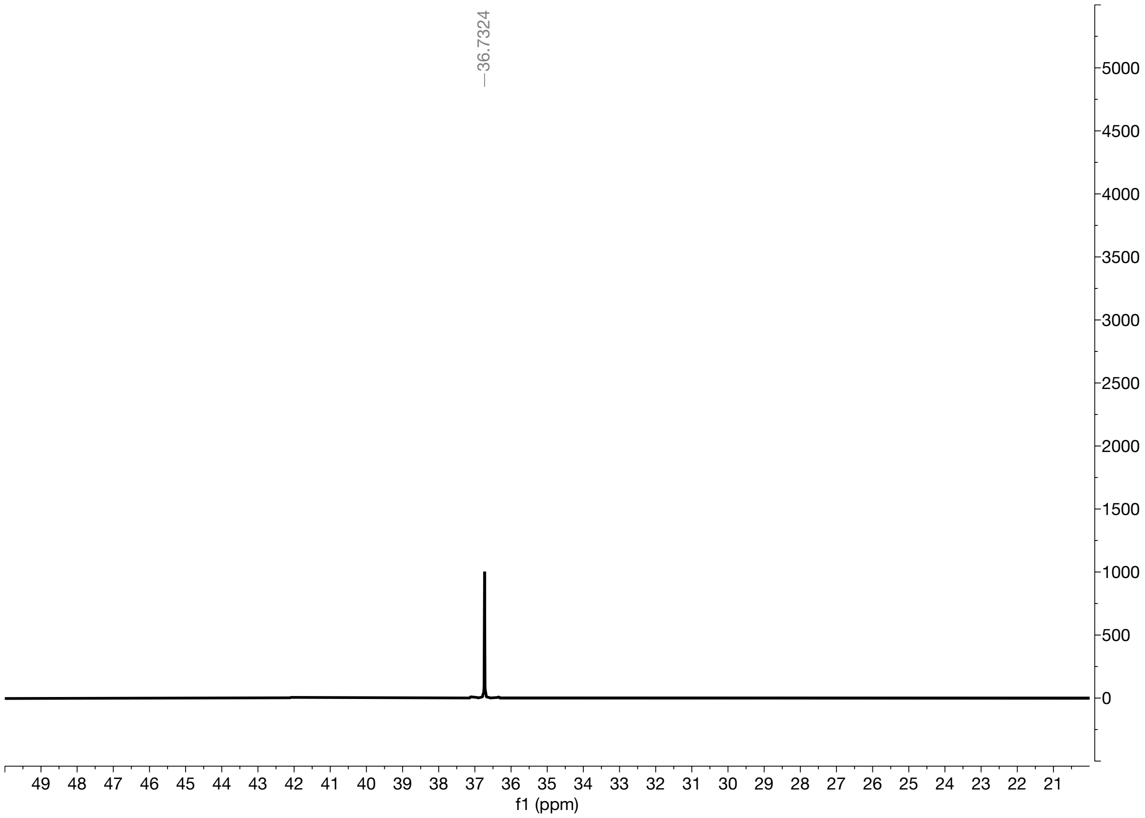

Figure S11. ^{31}P NMR (162 MHz, $CDCl_3$) of compound **2**.

2.3 Synthesis of 3.

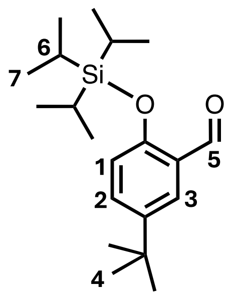

[15] Imidazole (0.83 g, 12.19 mmol) and TIPSCl (1.50 ml, 7.01 mmol) were added to a solution of 5-tert-Butyl-2-hydroxybenzaldehyde (0.90 ml, 4.98 mmol) in dimethylformamide (10 ml). The reaction mixture was allowed to stir at room temperature for 18 hours. The solution was then acidified with 0.1M HCl (aq.) and extracted with ethyl acetate. The organic layer was washed with 5% LiCl (aq.), brine and dried over Na_2SO_4 anhydrous. The solution was then concentrated under reduced pressure and the the pure product was afforded as a yellow oil by silica flash chromatography using a mixture of petroleum ether and dichloromethane: yield 1.65 g (99%).

^{1}H NMR (400 MHz, $CDCl_3$): δ_H = 10.54 (s, 1H, C<u>H</u>O), 7.81 (d, 1H, *J* = 2.7 Hz, H_{C3}), 7.47 (dd, 1H, *J* = 8.7, 2.7 Hz, H_{C2}), 6.83 (d, 1H, *J* = 8.7 Hz, H_{C1}), 1.35 (m, 3H, H_{C6}), 1.30 (s, 9H, H_{C4}), 1.12 (d, 18H, *J* = 7.5 Hz, H_{C7}).

^{13}C NMR (101 MHz, $CDCl_3$)**:** δ_C = 190.75 (C5), 157.46 (<u>C</u>-OTIPS), 144.02 (<u>C</u>-*t*Bu), 133.24 (C2), 126.06 (<u>C</u>-C5), 124.61 (C3), 119.39 (C1), 34.38 (C11), 31.41 (C7), 13.13 (C6).

HRMS (ESI$^+$)**:** calculated for $[C_{20}H_{35}O_2Si]^+$ 335.2315, found $[M+H]^+$ 335.2387.

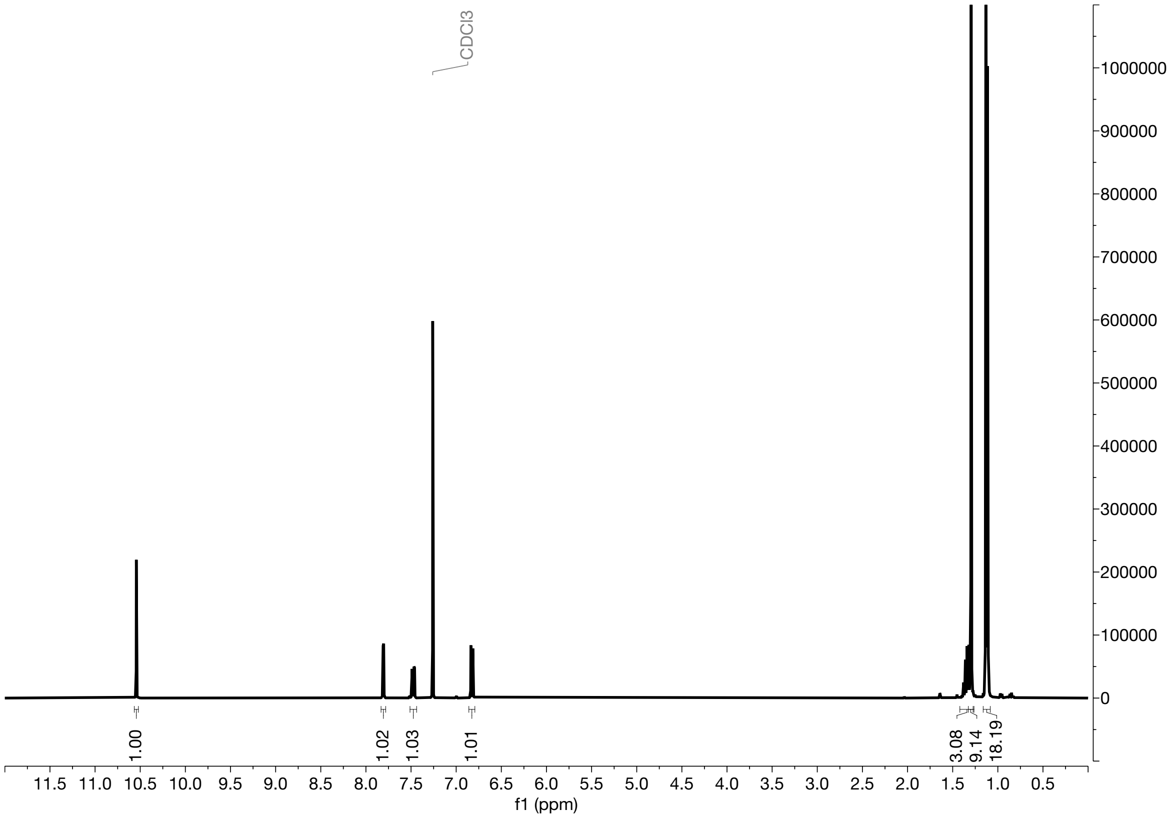

Figure S12. ^{1}H NMR (400 MHz, $CDCl_3$) of compound **15**.

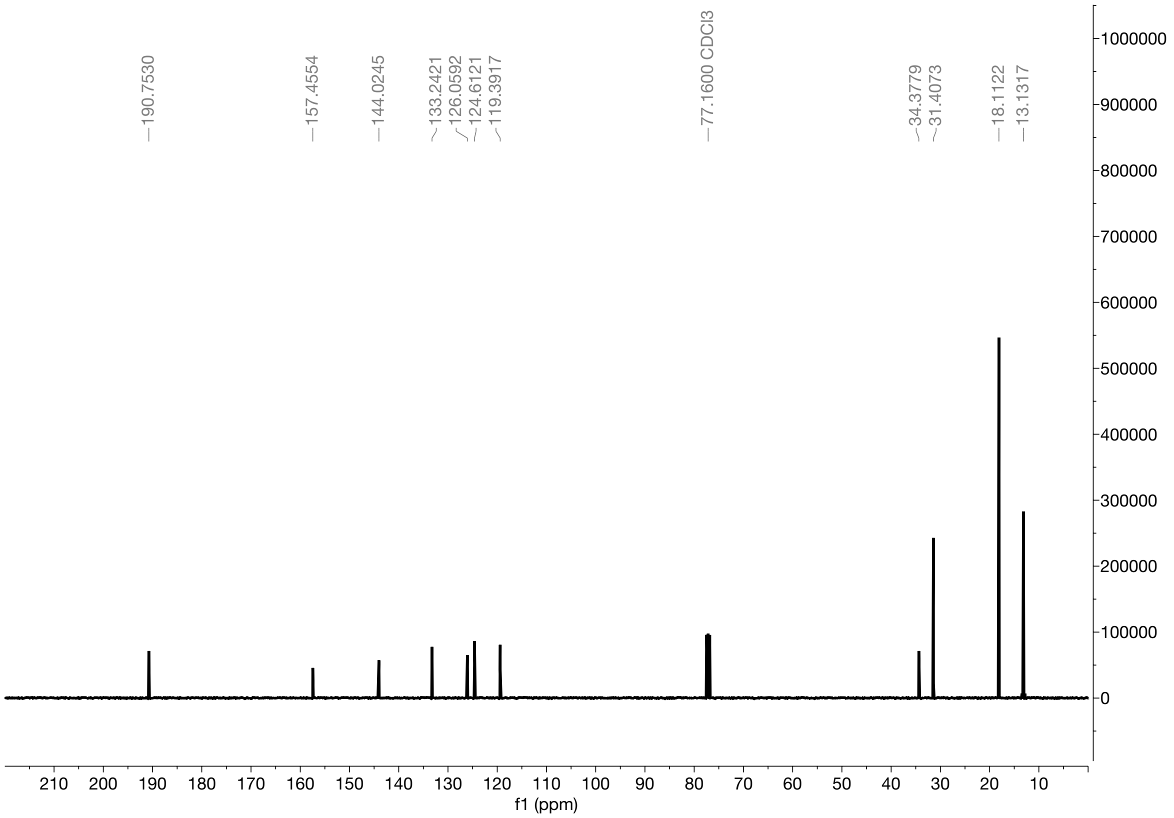

Figure S13. ^{13}C NMR (101 MHz, $CDCl_3$) of compound **15**.

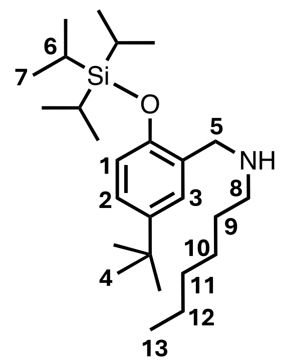

[21] A mixture of **15** (1.05 g, 3.14 mmol) and *n*-hexylamine (0.70 ml, 5.30 mmol) in dry dichloromethane (15 ml) was stirred over molecular sieves at room temperature for 18 hours. The reaction mixture was then cooled to 0 °C and diluted with methanol (15 ml). Sodium borohydride (0.68 g, 17.98 mmol) was added in small aliquots and the reaction was left stirring for 30 minutes. The solution was then quenched with water and filtered. After extracting with ethyl acetate, the organic layer was washed with brine and dried over Na_2SO_4 anhydrous. The solution was then concentrated under reduced pressure and the pure product was afforded as a yellow solid by silica flash chromatography using a mixture of dichloromethane and methanol: yield 1.15 g (87%).

^{1}H NMR (400 MHz, $CDCl_3$): δ_H = 7.23 (d, 1H, *J* = 2.5 Hz, H_{C3}), 7.09 (dd, 1H, *J* = 8.5, 2.6 Hz, H_{C2}), 6.71 (d, 1H, *J* = 8.4 Hz, H_{C1}), 3.78 (s, 2H, H_{C5}), 2.61 (t, 2H, *J* = 7.3, H_{C8}), 1.51 (p, 2H, *J* = 7.1 Hz, H_{C9}), 1.30 (m, 18H, H_{C4}, H_{C10-12}, H_{C6}), 1.11 (d, 18H, *J* = 7.5 Hz, H_{C7}), 0.87 (t, 3H, *J* = 6.2, H_{C13}).

^{13}C NMR (101 MHz, $CDCl_3$)**:** δ_C = 151.89 (C-OTIPS), 143.36 (C-*t*Bu), 129.02 (C-C5), 127.32 (C3), 124.69 (C2), 117.36 (C1), 50.41 (C5), 49.51 (C8), 34.18 (C-C4), 31.94 (C12), 31.69 (C4), 30.20 (C9), 27.30 (C10), 22.76 (C11), 18.26 (C7), 14.18 (C13), 13.23 (C6).

HRMS (ESI$^+$)**:** calculated for $[C_{26}H_{50}NOSi]^+$ 420.3656, found $[M+H]^+$ 420.3652.

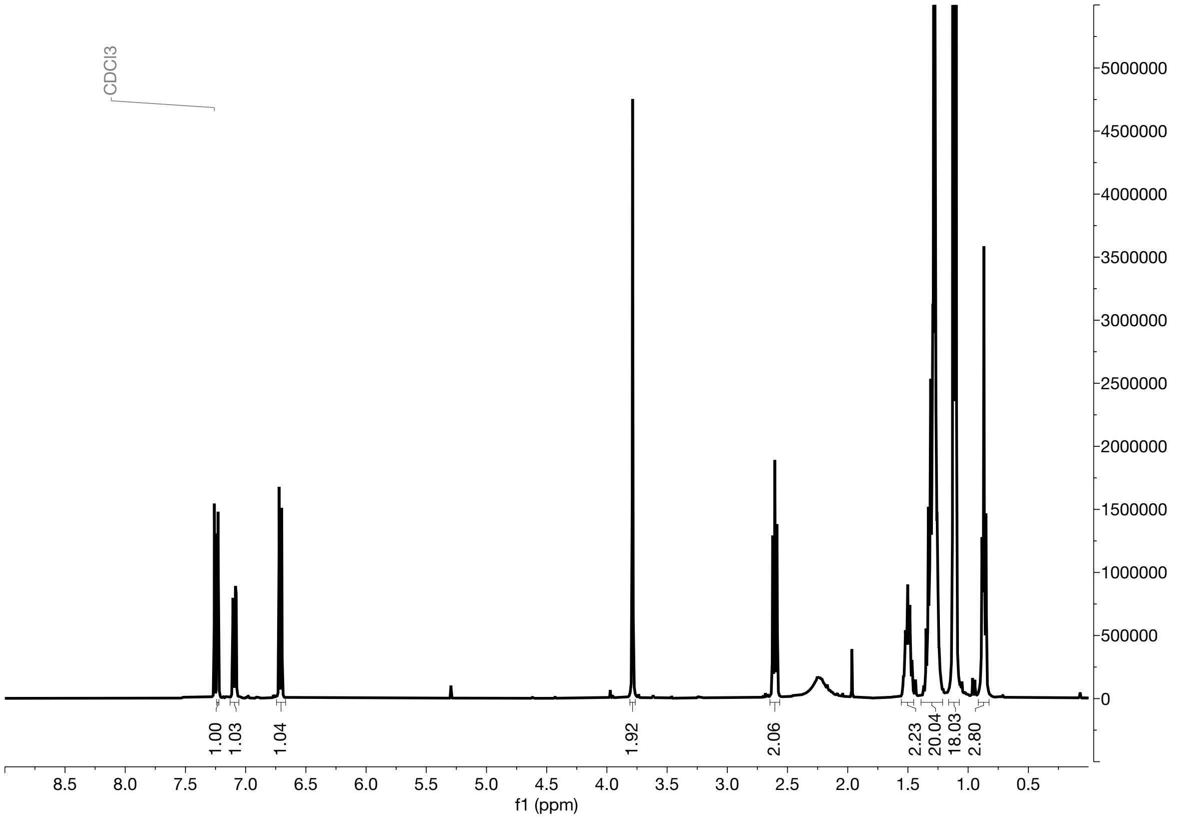

Figure S14. ^{1}H NMR (400 MHz, $CDCl_3$) of compound **21**.

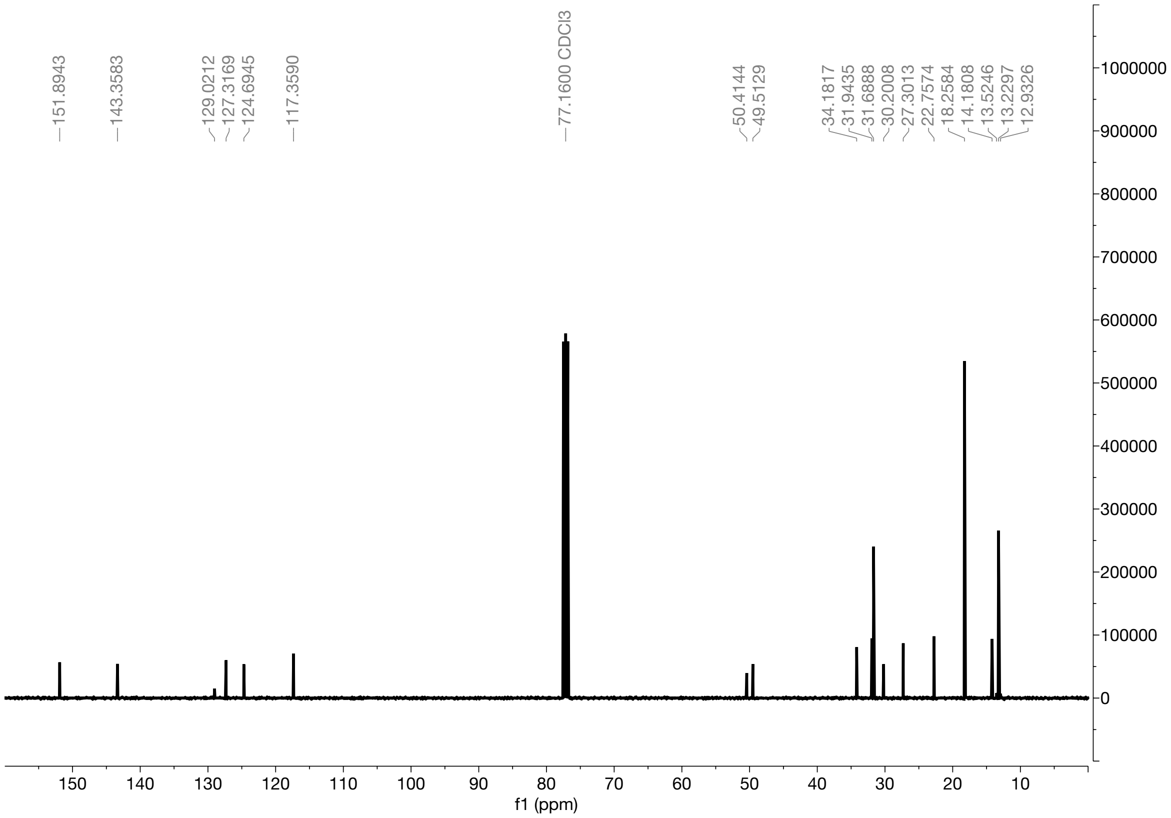

Figure S15. ^{13}C NMR (101 MHz, $CDCl_3$) of compound **21**.

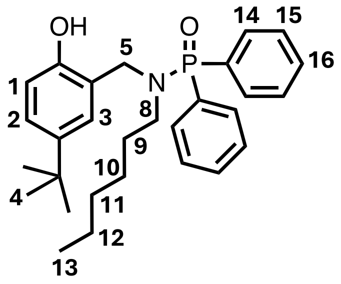

[3] Chlorodiphenylphosphine (0.60 ml, 3.25 mmol) and triethylamine (1.00 ml, 7.17 mmol) were added dropwise to a solution of **21** (1.09 ml, 2.61 mmol) in dry tetrahydrofuran (15 ml) at -78°C. The reaction mixture was allowed to stir at room temperature under inert atmosphere for 18 hours. Thereafter, the reaction mixture was cooled to 0 °C and 30% H_2O_2 (aq.) (0.40 ml, 3.91 mmol) was added. The mixture was left stirring for 1 hour before diluting it with iced water. After extracting with dichloromethane, the organic layer was washed with saturated NH_4Cl (aq.) and dried over Na_2SO_4 anhydrous. After concentrating the solution under reduced pressure, 0.20 g of the resulting oil was dissolved in tetrahydrofuran (1 ml) and the solution was cooled to 0 ºC. A 1M solution of TBAF in tetrahydrofuran (0.70 ml, 0.70 mmol) was, then, dropwise added and the mixture was stirred at room temperature for 30 minutes. After adding water (1 ml), the solution was extracted with ethyl acetate. The organic layer was then washed with brine, dried over Na_2SO_4 anhydrous and concentrated under reduced pressure. The final product was purified by preparative HPLC using a XSelect® CSH C18 (5 um, 19 x 150 mm) column with 25% acetonitrile in water: overall yield 0.53 g (43%).

^{1}H NMR (400 MHz, $CDCl_3$)**:** δ_H = 9.86 (s, 1H, O<u>H</u>), 7.79 (m, 4H, H_{C14}), 7.56 (m, 2H, H_{C16}), 7.48 (m, 4H, H_{C15}), 7.24 (dd, 1H, J = 8.5, 2.5 Hz, H_{C2}), 7.04 (d, 1H, J = 2.5 Hz, H_{C3}), 6.91 (d,

1H, J = 8.5 Hz, H_{C1}), 4.26 (d, 2H, J = 10.5 Hz, H(C5)), 2.70 (m, 2H, H_{C8}), 1.37 (p, 2H, J = 7.3 Hz, H_{C9}), 1.27 (s, 9H, H_{C4}), 1.12 (m, 2H, H_{C12}), 1.01 (m, 2H, H_{C11}), 0.92 (m, 2H, H_{C10}), 0.77 (t, 3H, J = 7.2 Hz, H_{C13}).

^{13}C NMR (101 MHz, $CDCl_3$)**:** δ_C = 154.57 (<u>C</u>-*t*Bu), 141.68 (<u>C</u>-C5), 132.46 (d, J = 10.0 Hz, C14), 132.41 (d, J = 2.8 Hz, C16), 130.97 (d, J = 128.2 Hz, <u>C</u>-P), 128.81 (d, J = 12.8 Hz, C15), 128.65 (C3), 126.79 (C2), 122.06 (d, J = 1.5 Hz, <u>C</u>-OH), 118.47 (C1), 47.26 (d, J = 4.4 Hz, C5), 46.19 (d, J = 4.5 Hz, C8), 33.95 (<u>C</u>-C4), 31.69 (C4), 31.35(C11), 28.50 (d, J = 3.1 Hz, C9), 26.41 (C10), 22.55 (C12), 14.02 (C13).

^{31}P NMR (162 MHz, $CDCl_3$): δ_P = 36.80.

HRMS (ESI+)**:** calculated for $[C_{29}H_{38}NO_2PNa]^+$ 486.2657, found $[M+H]^+$ 486.2552.

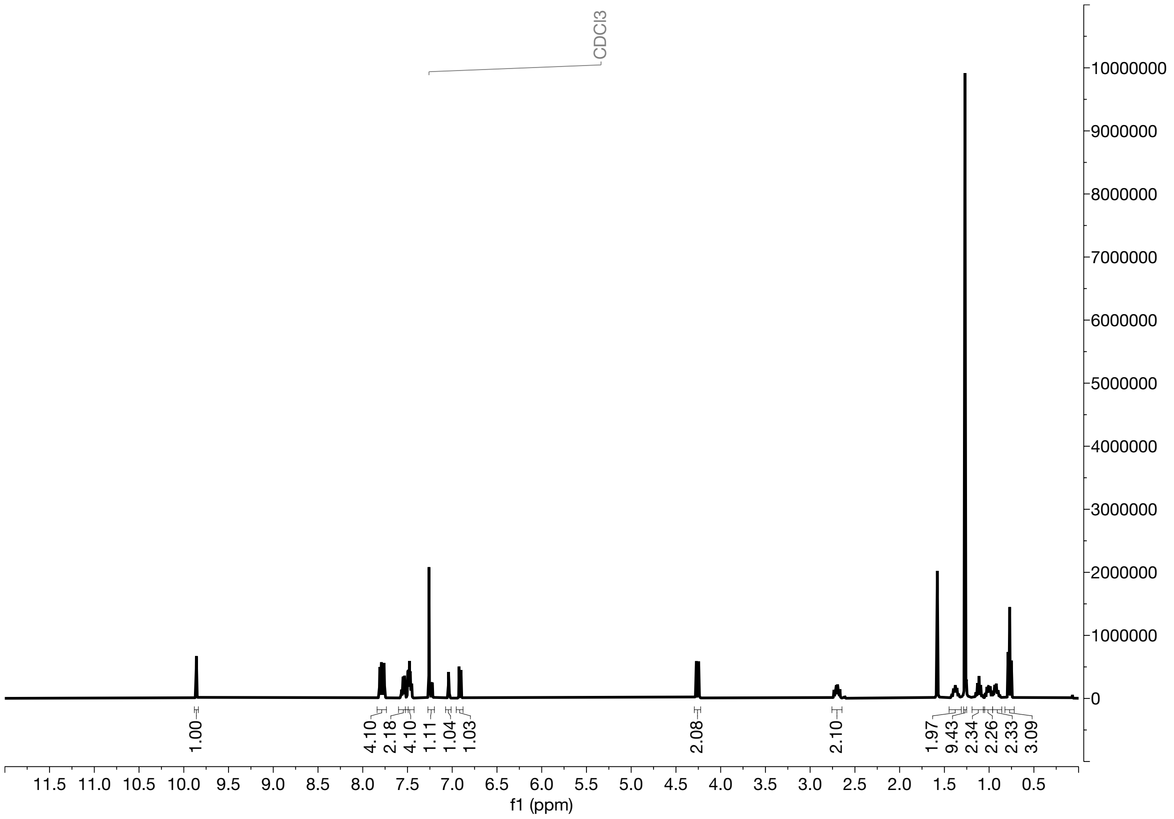

Figure S16. ^{1}H NMR (400 MHz, $CDCl_3$) of compound **3**.

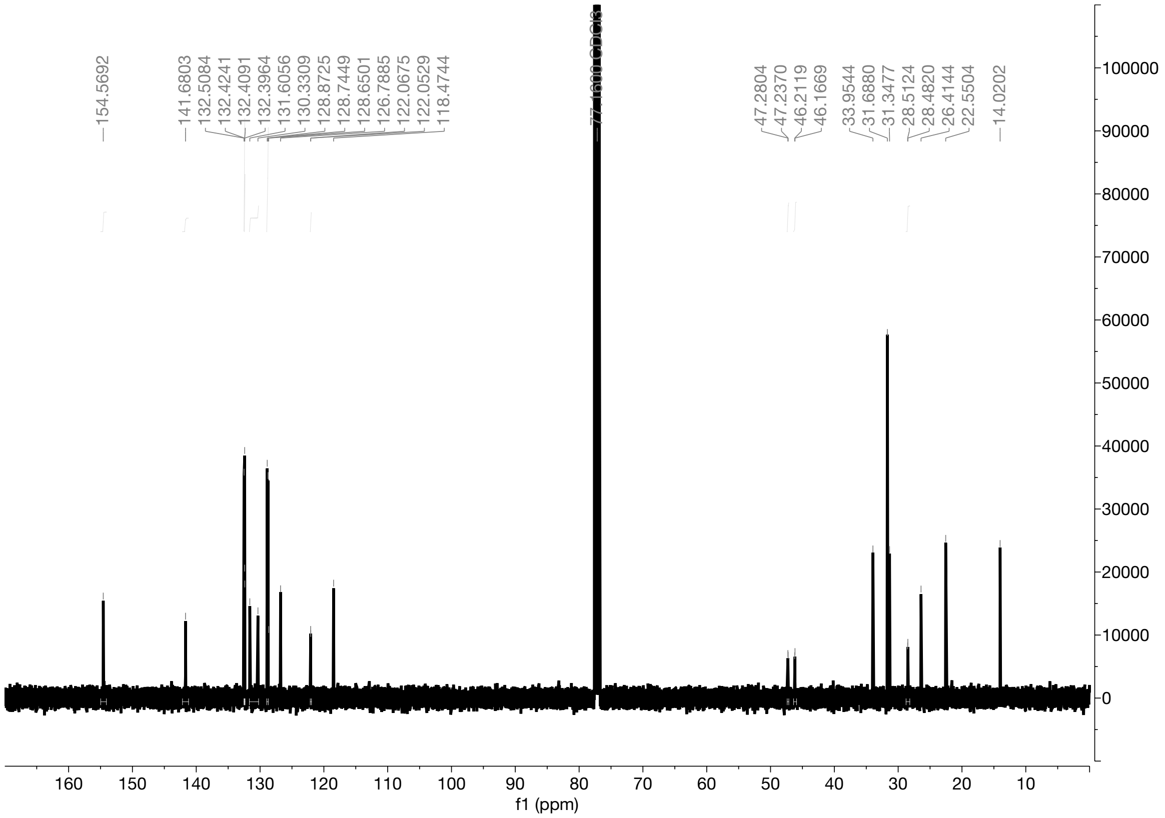

Figure S17. ^{13}C NMR (101 MHz, $CDCl_3$) of compound **3**.

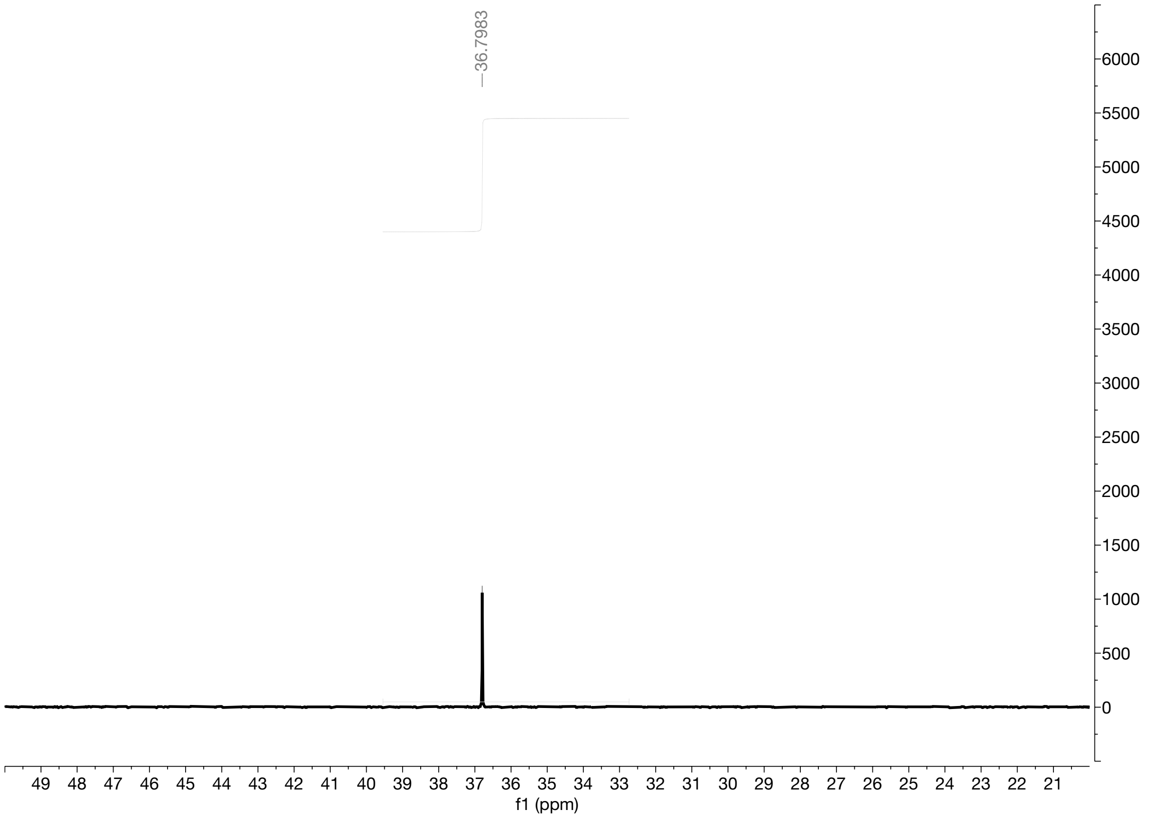

Figure S18. ^{31}P NMR (162 MHz, $CDCl_3$) of compound **3**.

2.4 SYNTHESIS OF 4.

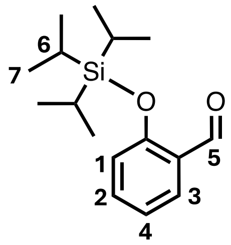

[16] Imidazole (2.51 g, 36.93 mmol) and TIPSCl (5.00 ml, 23.37 mmol) were added to a solution of salicylaldehyde (2.00 ml, 18.77 mmol) in dimethylformamide (35 ml). The reaction mixture was allowed to stir at room temperature for 18 hours. The solution was then acidified with 0.1M HCl (aq.) and extracted with ethyl acetate. The organic layer was washed with 5% LiCl (aq.), brine and dried over Na_2SO_4 anhydrous. The solution was then concentrated under reduced pressure and the pure product was afforded as a transparent oil by silica flash chromatography using a mixture of petroleum ether and ethyl acetate: yield 5.13 g (98%).

^{1}H NMR (500 MHz, $CDCl_3$): δ_H = 10.55 (d, 1H, J = 0.9 Hz, C<u>H</u>O), 7.80 (dd, 1H, J = 7.8, 1.9 Hz, H_{C1}), 7.44 (m, 1H, H_{C4}), 7.00 (m, 1H, H_{C2}), 6.89 (dd, 1H, J = 8.3, 1.0 Hz, H_{C3}), 1.35 (m, 3H, H_{C6}), 1.13 (d, 18H, J = 7.5 Hz, H_{C7}).

^{13}C NMR (126 MHz, $CDCl_3$): δ_C = 190.41 (C5), 159.56 (<u>C</u>-C5), 135.86 (C4), 128.43 (C1), 126.94 (<u>C</u>-OTIPS), 121.27 (C2), 119.88 (C3), 18.08 (C7), 13.13 (C6).

HRMS (ESI+)**:** calculated for $[C_{16}H_{27}O_2Si]^+$ 279.1717, found $[M+H]^+$ 279.1784.

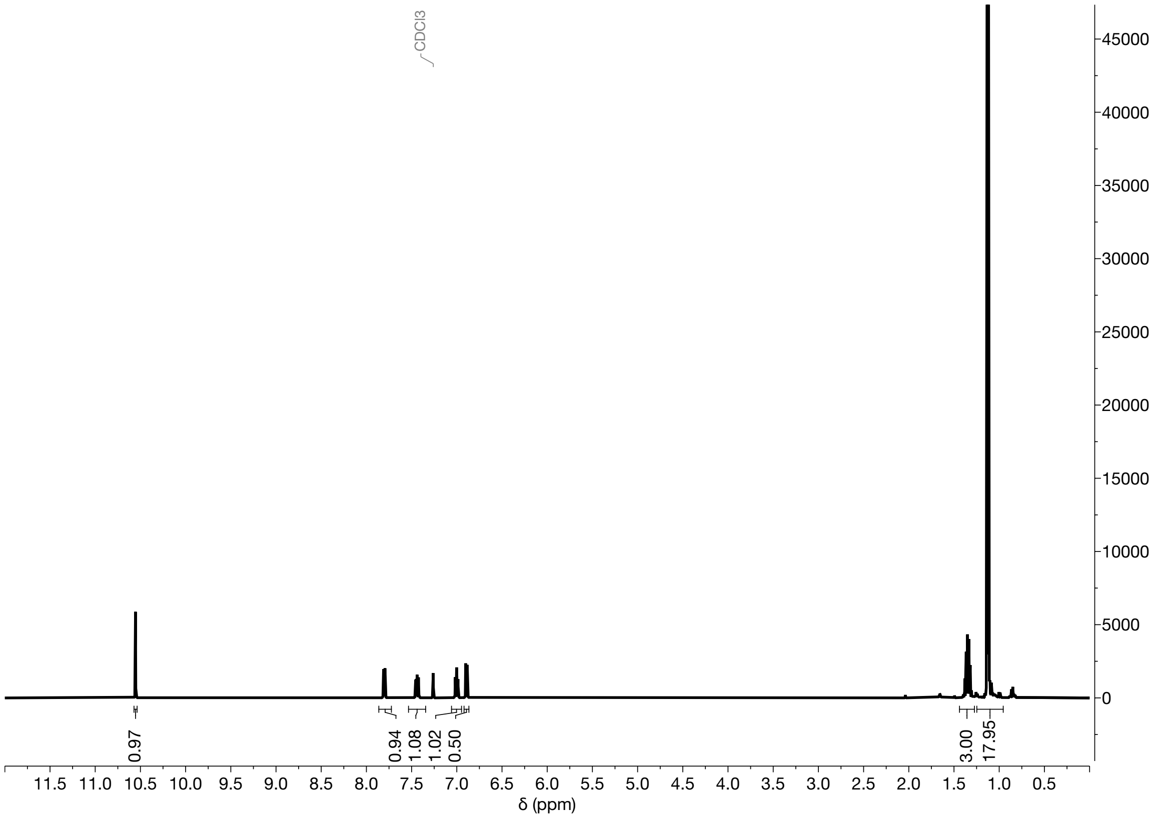

Figure S19. ^{1}H NMR (500 MHz, $CDCl_3$) of compound **16**.

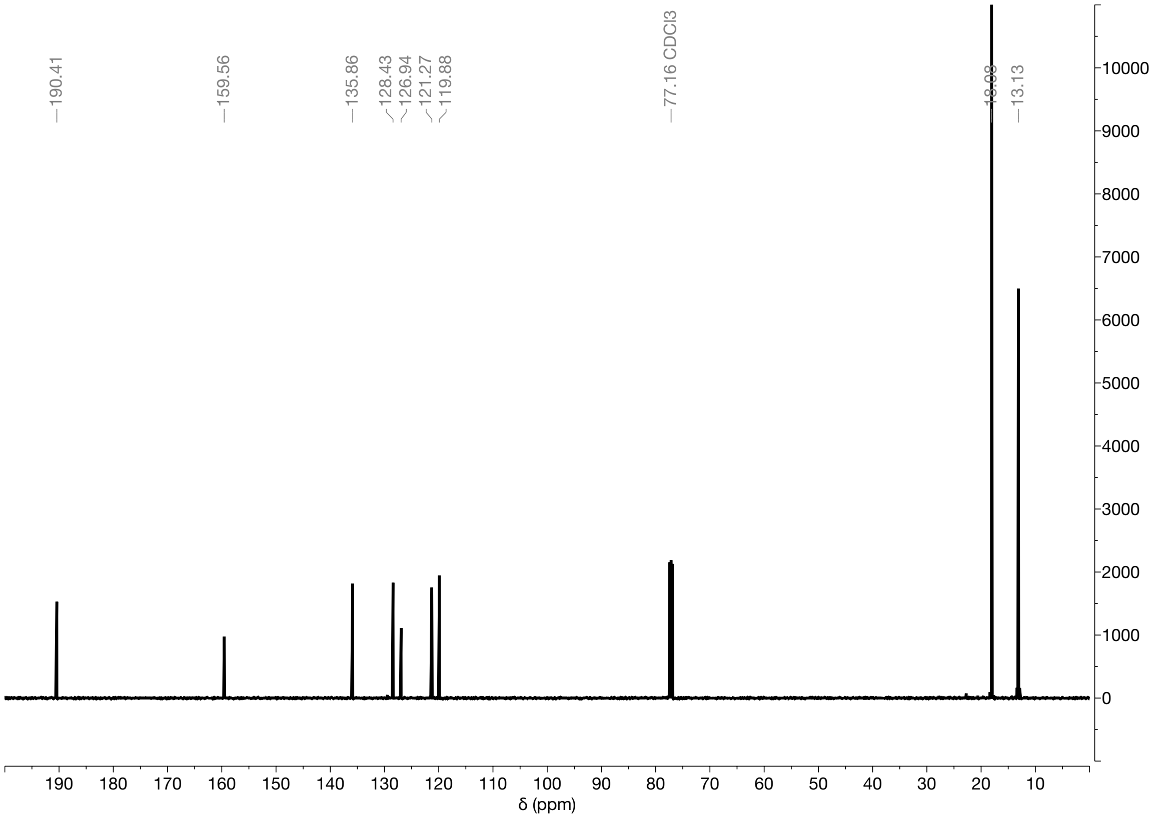

Figure S20. ^{13}C NMR (126 MHz, $CDCl_3$) of compound **16**.

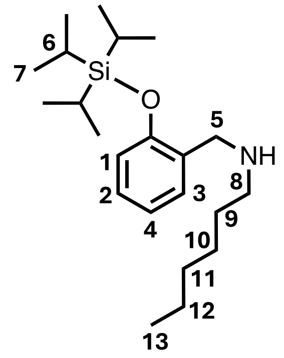

[22] A mixture of **16** (0.56 g, 2.02 mmol) and *n*-hexylamine (0.40 ml, 3.03 mmol) in dry dichloromethane (10 ml) was stirred over molecular sieves at room temperature for 3 hours. The reaction mixture was then cooled to 0 °C and diluted with methanol (10 ml). Sodium borohydride (0.40 g, 10.57 mmol) was added in small aliquots and the reaction was left stirring for 2 hours. The solution was then quenched with water and filtered. After extracting with ethyl acetate, the organic layer was washed with brine and dried over Na_2SO_4 anhydrous. The solution was then concentrated under reduced pressure and the resulting light-yellow oil was used directly in the next step without further purification.

^{1}H NMR (400 MHz, $CDCl_3$): δ_H = 7.23 (dd, 1H, *J* = 7.5, 1.8 Hz, H(C1)), 7.10 (m, 1H, H(C4)), 6.89 (m, 1H, H(C2)), 6.80 (dd, 1H, *J* = 8.1, 1.2 Hz, H(C3)), 3.80 (s, 2H, H(C5)), 2.59 (t, 2H, *J* = 7.1 Hz, H(C8)), 1.50 (p, 2H, *J* = 7.6 Hz, H(C9)), 1.29 (m, 9H, H(C10-11-12), H(C6)), 1.12 (d, 18H, *J* = 7.4 Hz, H(C7)), 0.87 (t, 3H, *J* = 6.6 Hz, H(C13)).

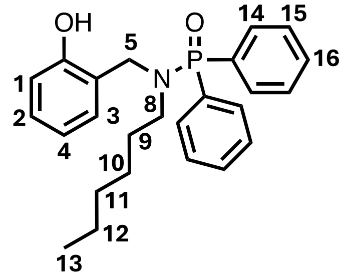

[4] Chlorodiphenylphosphine oxide (0.6 ml, 3.14 mmol) and triethylamine (0.45 ml, 3.23 mmol) were added dropwise to a solution of **22** (0.58 ml, 1.59 mmol) in dry tetrahydrofuran (10 ml). The reaction mixture was allowed to stir at room temperature under inert atmosphere for 18 hours. After evaporating tetrahydrofuran, the residue was redissolved in ethyl acetate and the organic layer was washed with 5% HCl (aq), water and brine, and dried over Na_2SO_4 anhydrous. After concentrating the solution under reduced pressure, 0.11 g of the resulting oil was dissolved in tetrahydrofuran (1 ml) and the solution was cooled to 0 ºC. A 1M solution of TBAF in tetrahydrofuran (0.40 ml, 0.40 mmol) was, then, dropwise added and the mixture was stirred at room temperature for 30 minutes. After adding water (1 ml), the solution was extracted with ethyl acetate. The organic layer was then washed with brine, dried over Na_2SO_4 anhydrous and concentrated under reduced pressure. The final product was purified by preparative HPLC using a XSelect® CSH C18 (5 um, 19 x 150 mm) column with 75% acetonitrile in water: overall yield 0.13 g (20%).

^{1}H NMR (500 MHz, $CDCl_3$)**:** δ_H = 10.06 (bs, 1H, O<u>H</u>), 7.77 (m, 4H, H_{C14}), 7.55 (m, 2H, H_{C16}), 7.48 (m, 4H, H_{C15}), 7.22 (m, 1H, H_{C2}), 7.05 (dd, 1H, *J* = 7.5, 1.7 Hz, H_{C3}), 6.98 (dd, 1H, *J* = 8.1, 1.2 Hz, H_{C1}), 6.79 (m, 1H, H_{C4}), 4.27 (d, 2H, *J* = 10.4 Hz, H_{C5}), 2.71 (m, 2H, H_{C8}), 1.37 (p, 2H, *J* = 7.5 Hz, H_{C9}), 1.12 (m, 2H, H_{C12}), 0.98 (m, 2H, H_{C11}), 0.90 (m, 2H, H_{C10}), 0.78 (t, 3H, *J* = 7.3 Hz, H_{C13}).

^{13}C NMR (126 MHz, $CDCl_3$)**:** δ_C = 157.06 (<u>C</u>-OH), 132.48 (C14), 132.43 (d, *J* = 7.6 Hz, C16), 131.76 (C3), 130.79 (d, *J* = 128.3 Hz, <u>C</u>-P), 130.10 (C2), 128.84 (d, *J* = 12.9 Hz, C15), 122.77 (<u>C</u>-C5), 119.15 (C4), 119.02 (C1), 46.83 (d, *J* = 3.9 Hz, C5), 46.06 (d, *J* = 4.2 Hz, C8), 31.24 (C11), 28.46 (d, *J* = 2.7 Hz, C9), 26.34 (C10), 22.50 (C12), 14.02 (C13).

^{31}P NMR (203 MHz, $CDCl_3$): δ_P = 37.13.

HRMS (ESI+) calculated for $[C_{25}H_{30}NO_2PNa]^+$ 430.2033, found $[M+Na]^+$ 430.1924.

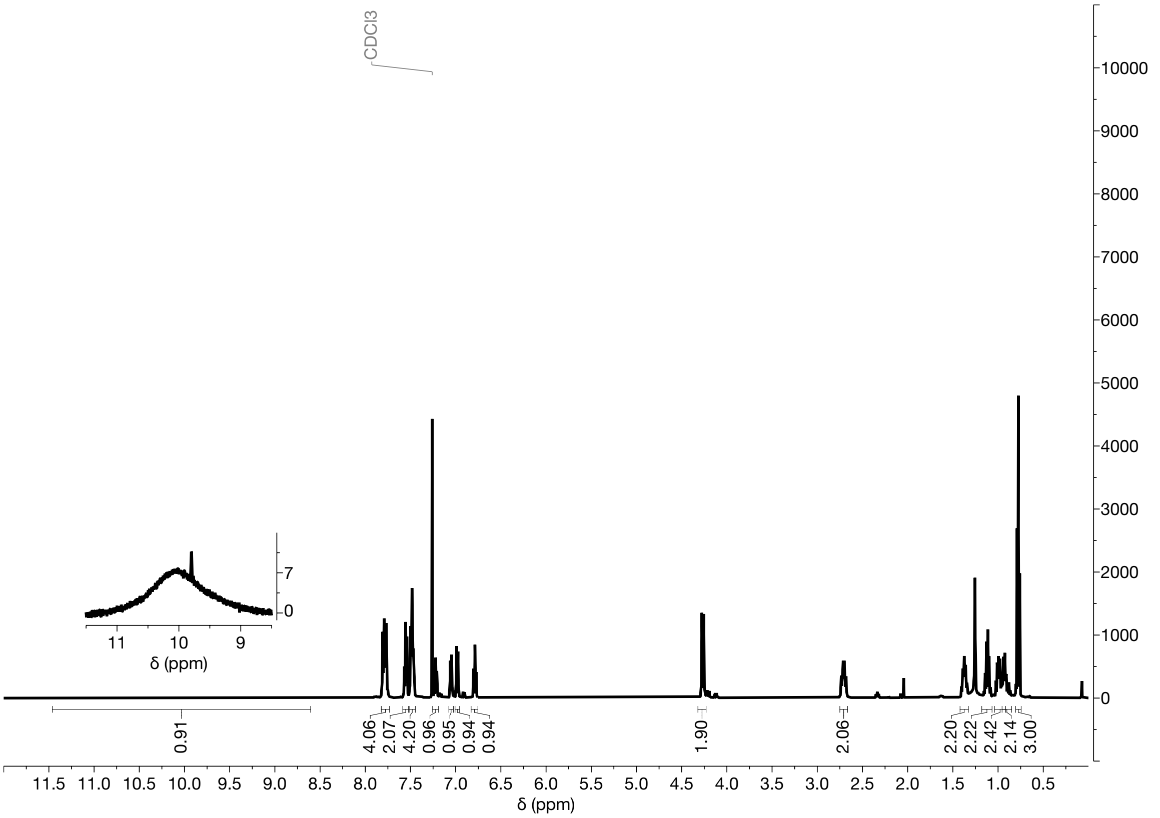

Figure S21. ^{1}H NMR (500 MHz, $CDCl_3$) of compound **4**.

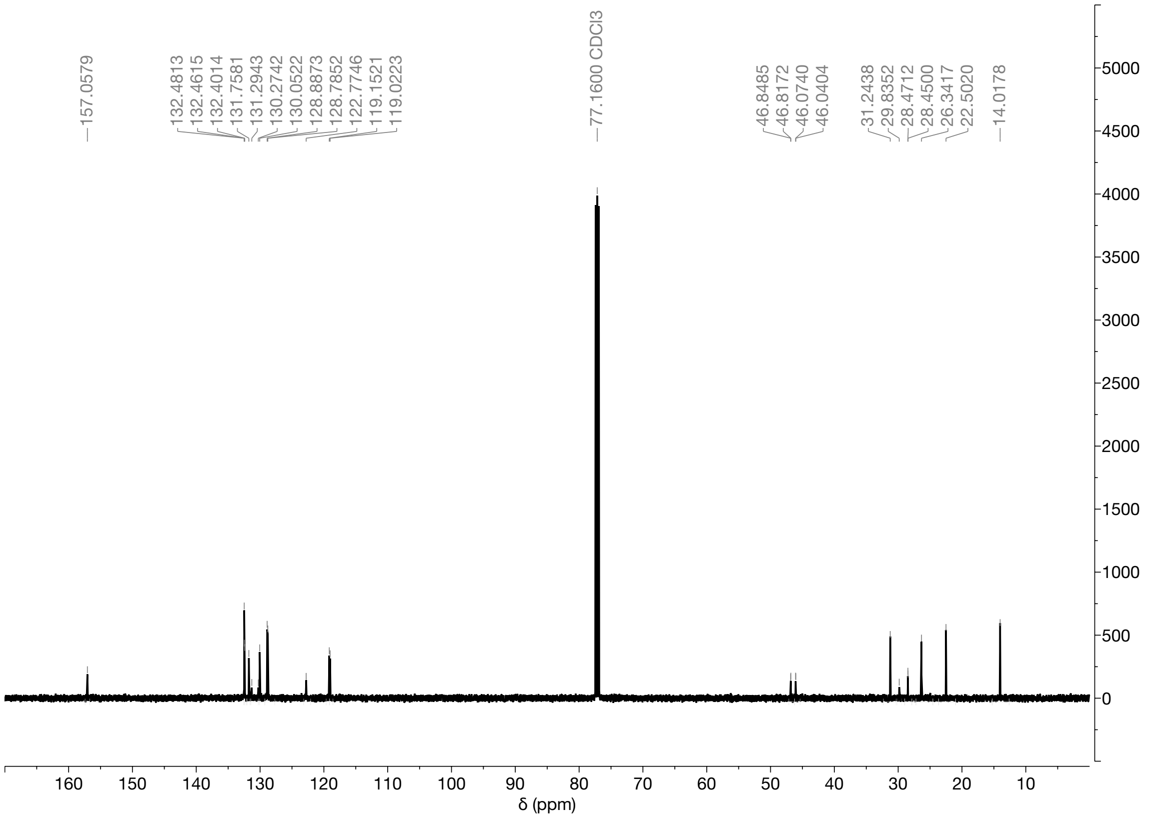

Figure S22. ^{13}C NMR (126 MHz, $CDCl_3$) of compound **4**.

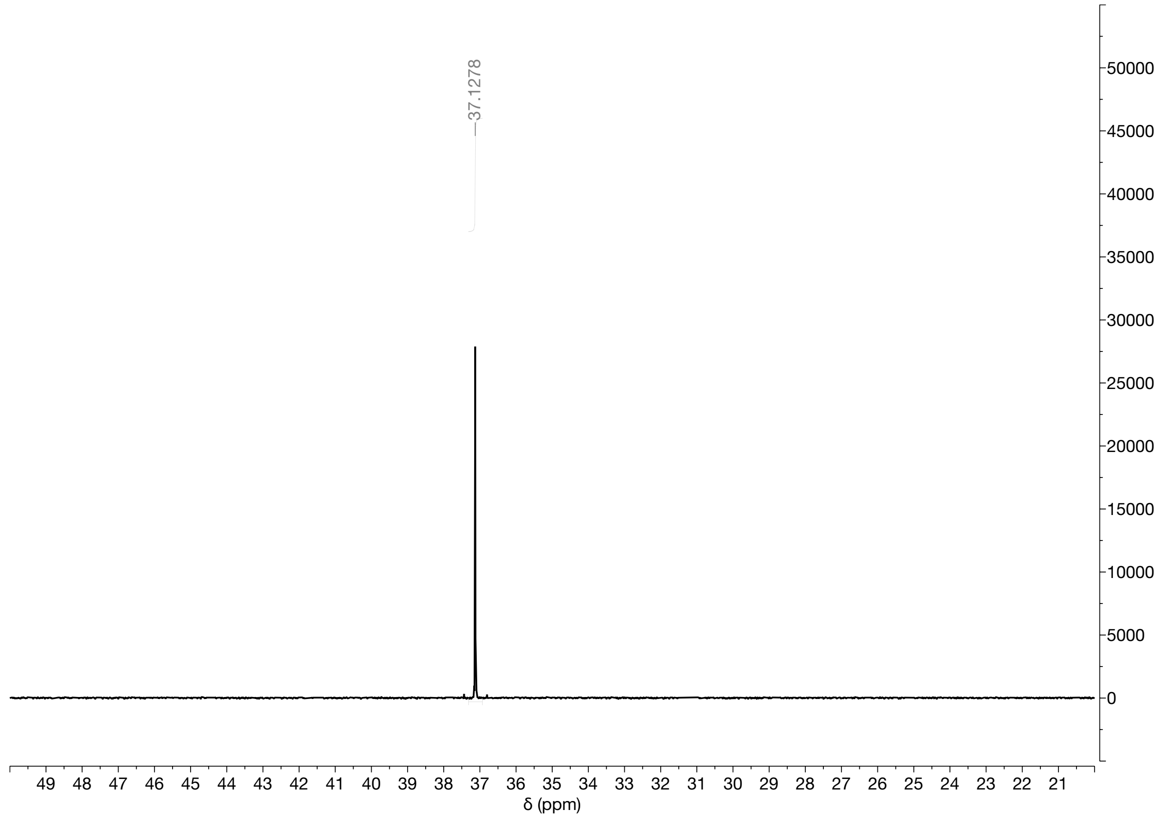

Figure S23. ^{31}P NMR (203 MHz, $CDCl_3$) of compound **4**.

2.5 SYNTHESIS OF 5.

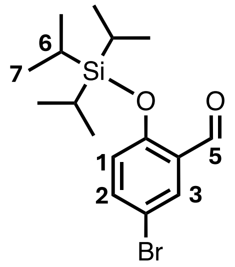

[17] Imidazole (1.76 g, 25.85 mmol) and TIPSCl (3.00 ml, 14.02 mmol) were added to a solution of 5-bromosalicylaldehyde (2.55 g, 12.43 mmol) in dimethylformamide (25 ml). The reaction mixture was allowed to stir at room temperature for 18 hours. The solution was then acidified with 0.1M HCl (aq.) and extracted with ethyl acetate. The organic layer was washed with 5% LiCl (aq.), brine and dried over Na_2SO_4 anhydrous. The solution was then concentrated under reduced pressure and the pure product was afforded as a white solid by basic alumina flash chromatography using a mixture of petroleum ether and dichloromethane: yield 4.40 g (99%).

^{1}H NMR (400 MHz, $CDCl_3$): δ_H = 10.44 (s, 1H, C<u>H</u>O), 7.90 (d, 1H, *J* = 2.7 Hz, H_{C3}), 7.52 (dd, 1H, *J* = 8.8, 2.7 Hz, H_{C2}), 6.79 (d, 1H, *J* = 8.8 Hz, H_{C1}), 1.34 (m, 3H, H_{C6}), 1.12 (d, 18H, *J* = 7.4 Hz, H_{C7}).

^{13}C NMR (101 MHz, $CDCl_3$): δ_C = 188.93 (<u>C</u>HO), 158.48 (<u>C</u>-OTIPS), 138.34 (C2), 131.14 (C3), 128.19 (<u>C</u>-C5), 121.77 (C1), 113.95 (<u>C</u>-Br), 55.87 (C4), 18.04 (C7), 13.09 (C6).

HRMS (ESI+)**:** calculated for $[C_{16}H_{25}BrO_2Si]^+$ 356.0809, found $[M]^+$ 356.0812.

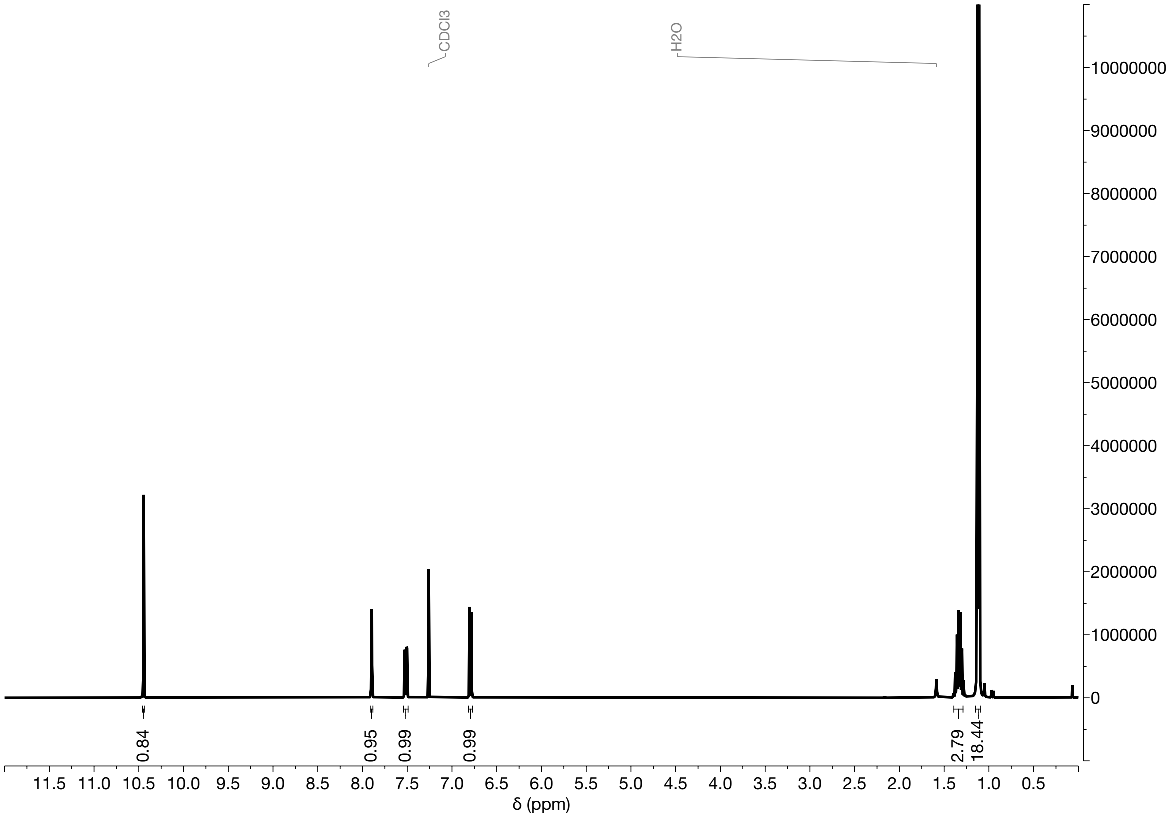

Figure S24. ^{1}H NMR (400 MHz, $CDCl_3$) of compound **17**.

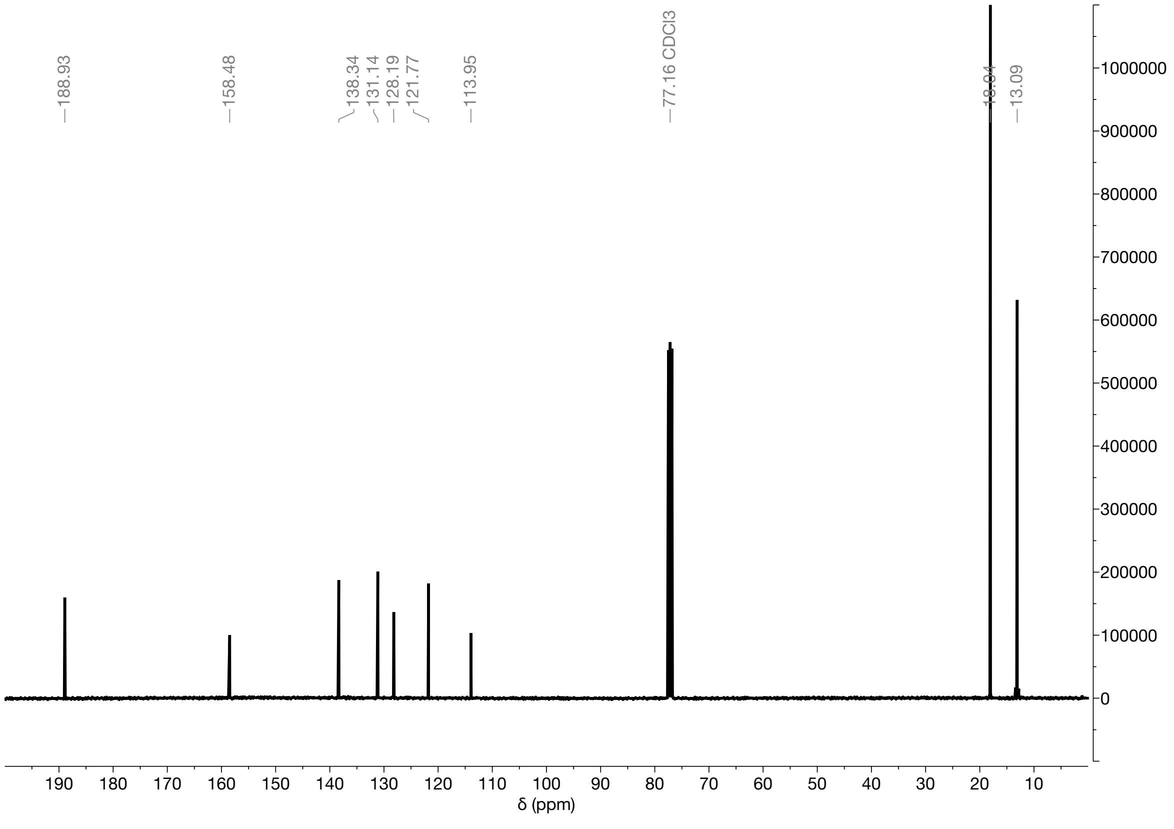

Figure S25. ^{13}C NMR (101 MHz, $CDCl_3$) of compound **17**.

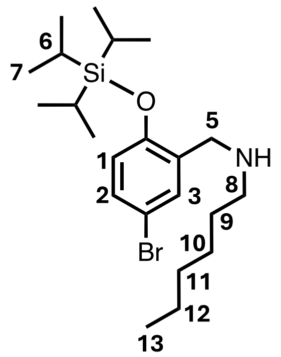

[23] A mixture of **17** (1.08 g, 3.02 mmol) and *n*-hexylamine (0.60 ml, 4.54 mmol) in dry dichloromethane (15 ml) was stirred over molecular sieves at room temperature for 18 hours. The reaction mixture was then cooled to 0 °C and diluted with methanol (15 ml). Sodium borohydride (0.62 g, 16.39 mmol)was added in small aliquots and the reaction was left stirring for 30 minutes. The solution was then quenched with water and filtered. After extracting with ethyl acetate, the organic layer was washed with brine and dried over Na_2SO_4 anhydrous. The solution was then concentrated under reduced pressure and the pure product was afforded as a white solid by silica flash chromatography using a mixture of dichloromethane and methanol: yield 1.11 g (83%).

^{1}H NMR (400 MHz, $CDCl_3$): δ_H = 7.38 (d, 1H, *J* = 2.6 Hz, H_{C3}), 7.19 (dd, 1H, *J* = 8.6, 2.6 Hz, H_{C2}), 6.66 (d, 1H, *J* = 8.6 Hz, H_{C1}), 3.75 (s, 2H, H_{C5}), 2.58 (t, 2H, *J* = 7.2, H_{C8}), 1.48 (p, 2H, *J* = 6.9 Hz, H_{C9}), 1.29 (m, 9H, H_{C10-12}, H_{C6}), 1.10 (d, 18H, *J* = 7.4 Hz, H_{C7}), 0.87 (t, 3H, *J* = 6.4, H_{C13}).

^{13}C NMR (101 MHz, $CDCl_3$)**:** δ_C = 153.37 (C-OTIPS), 133.00 (C-C5), 132.66 (C3), 130.53 (C2), 119.61 (C1), 113.12 (C-Br), 49.56 (C8), 49.43 (C5), 31.93 (C12), 30.35 (C9), 27.23 (C10), 22.75 (C11), 18.16 (C7), 14.18 (C13), 13.15 (C6).

HRMS (ESI$^+$)**:** calculated for $[C_{22}H_{41}BrNOSi]^+$ 442.2135, found $[M+H]^+$ 442.2136.

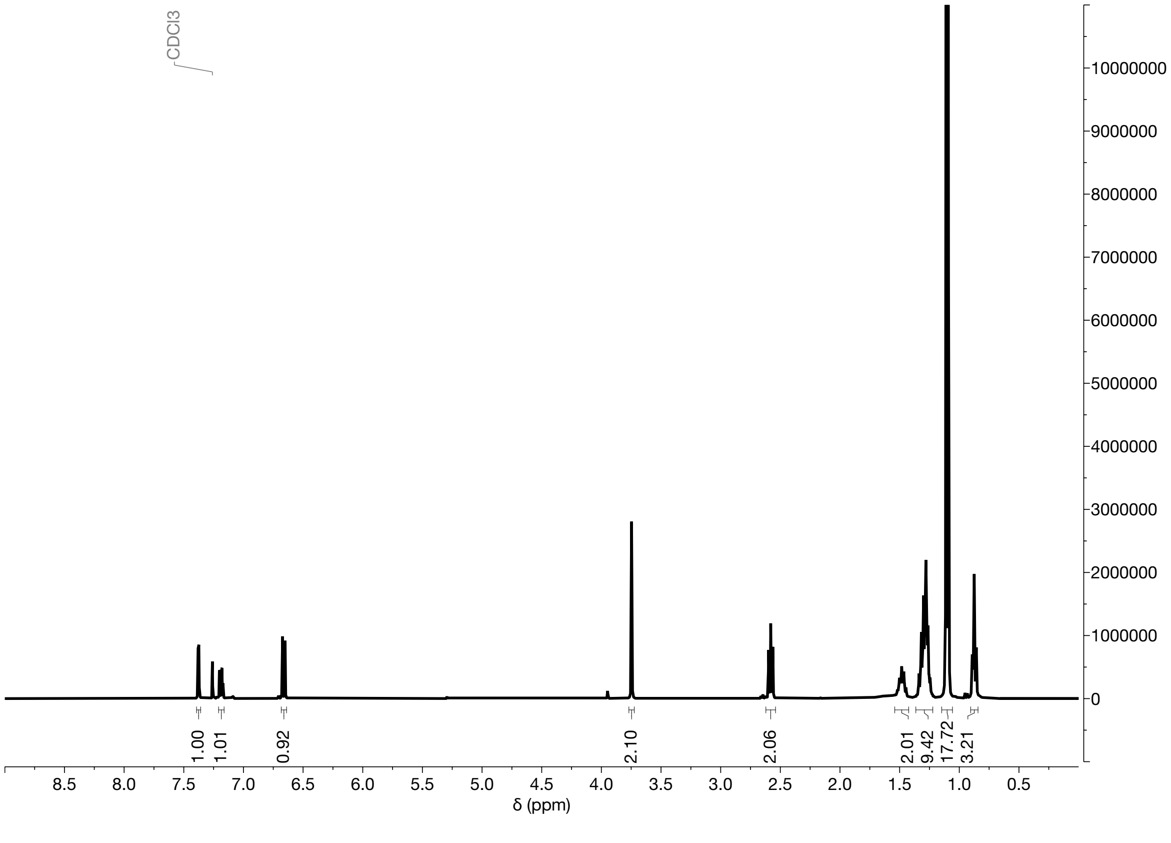

Figure S26. ^{1}H NMR (400 MHz, $CDCl_3$) of compound **23**.

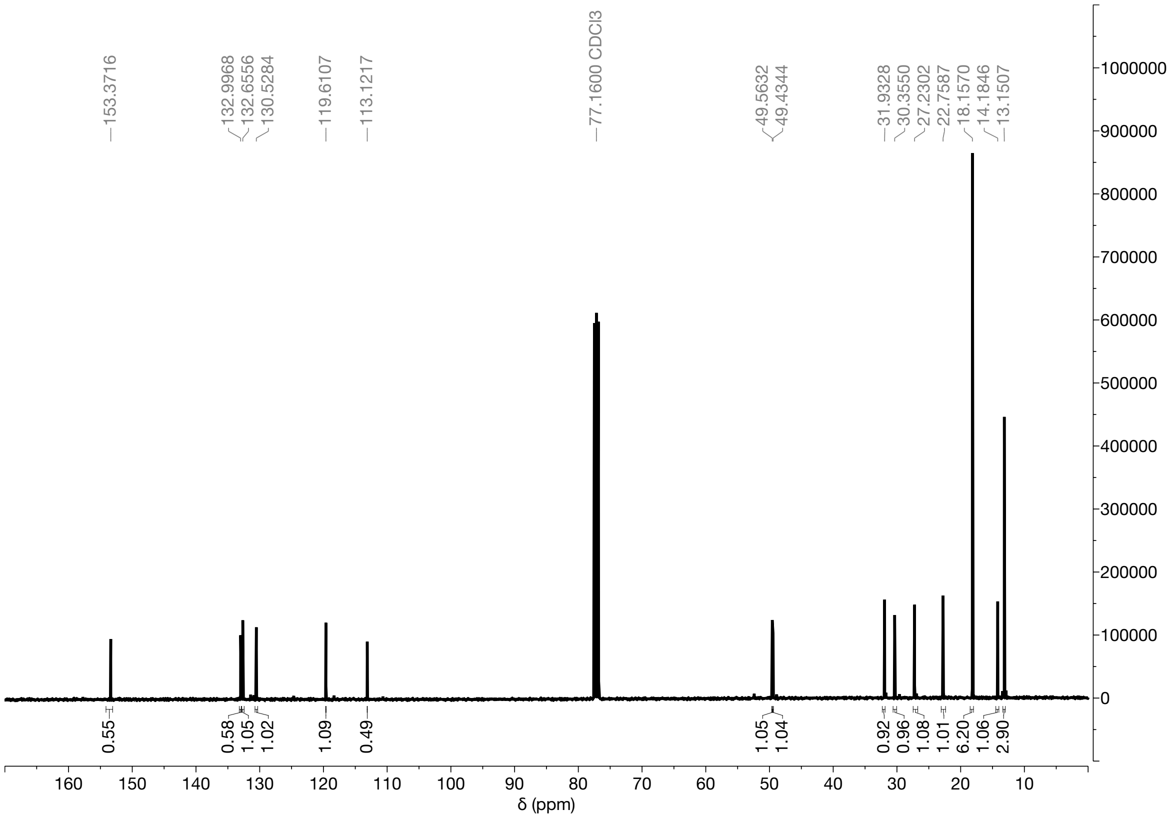

Figure S27. ^{13}C NMR (101 MHz, $CDCl_3$) of compound **23**.

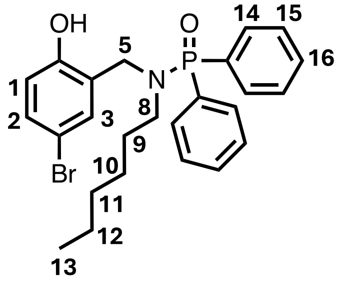

[5] Chlorodiphenylphosphine (0.60 ml, 3.25 mmol) and triethylamine (0.80 ml, 5.74 mmol) were added dropwise to a solution of **23** (1.03 g, 2.33 mmol) in dry tetrahydrofuran (15 ml) at -78°C. The reaction mixture was allowed to stir at room temperature under inert atmosphere for 18 hours. Thereafter, the reaction mixture was cooled to 0 °C and 30% H_2O_2 (aq.) (0.40 ml, 3.91 mmol) was added. The mixture was left stirring for 1 hour before diluting it with iced water After extracting with dichloromethane, the organic layer was washed with saturated NH_4Cl (aq.) and dried over Na_2SO_4 anhydrous. After concentrating the solution under reduced pressure, 0.20 g of the resulting oil was dissolved in tetrahydrofuran (1 ml) and the solution was cooled to 0 °C. A 1M solution of TBAF in tetrahydrofuran (0.70 ml, 0.70 mmol) was, then, dropwise added and the mixture was stirred at room temperature for 30 minutes. After adding water (1 ml), the solution was extracted with ethyl acetate. The organic layer was then washed with brine, dried over Na_2SO_4 anhydrous and concentrated under reduced pressure. The final product was purified by preparative HPLC using a XSelect® CSH C18 (5 um, 19 x 150 mm) column with 30% acetonitrile in water: overall yield 0.70 g (62%).

^{1}H NMR (400 MHz, $CDCl_3$)**:** δ_H = 10.42 (s, 1H, O<u>H</u>), 7.77 (m, 4H, H_{C14}), 7.57 (m, 2H, H_{C16}), 7.49 (m, 4H, H_{C15}), 7.30 (dd, 1H, J = 8.7, 2.5 Hz, H_{C2}), 7.15 (d, 1H, J = 2.5 Hz, H_{C3}), 6.86 (d,

1H, J = 8.7 Hz, H_{C1}), 4.20 (d, 2H, J = 10.5 Hz, H_{C5}), 2.72 (m, 2H, H_{C8}), 1.35 (p, 2H, J = 7.5 Hz, H_{C9}), 1.14 (m, 2H, H_{C12}), 1.01 (m, 2H, H_{C11}), 0.94 (m, 2H, H_{C10}), 0.79 (t, 3H, J = 7.3 Hz, H_{C13}).

^{13}C NMR (101 MHz, $CDCl_3$)**:** δ_C = 156.39 (C-OH), 133.95 (C3), 132.79 (C2), 132.64 (d, J = 2.8 Hz, C16), 132.41 (d, J = 10.1 Hz, C14), 130.46 (d, J = 128.1 Hz, C-P), 128.92 (d, J = 12.9 Hz, C15), 125.07 (d, J = 1.4 Hz, C-C5), 121.06 (C1), 110.71 (C-Br), 46.61 (d, J = 4.3 Hz, C5), 46.32 (d, J = 4.5 Hz, C8), 31.22 (C11), 28.48 (d, J = 2.7 Hz, C9), 26.29 (C10), 22.51 (C12), 14.05 (C13).

^{31}P NMR (162 MHz, $CDCl_3$): δ_P = 37.76.

HRMS (ESI+) calculated for $[C_{25}H_{30}BrNO_2P]^+$ 486.1192, found $[M+H]^+$ 486.1188.

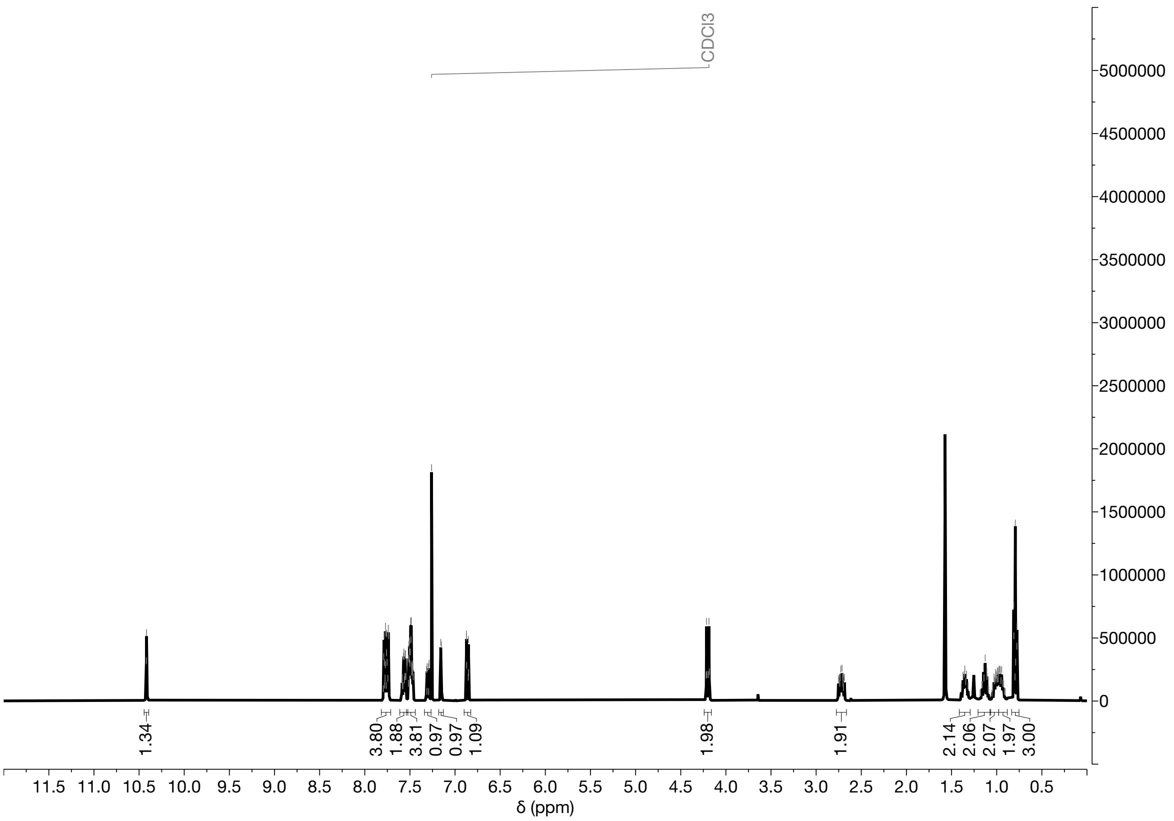

Figure S28. ^{1}H NMR (400 MHz, $CDCl_3$) of compound **5**.

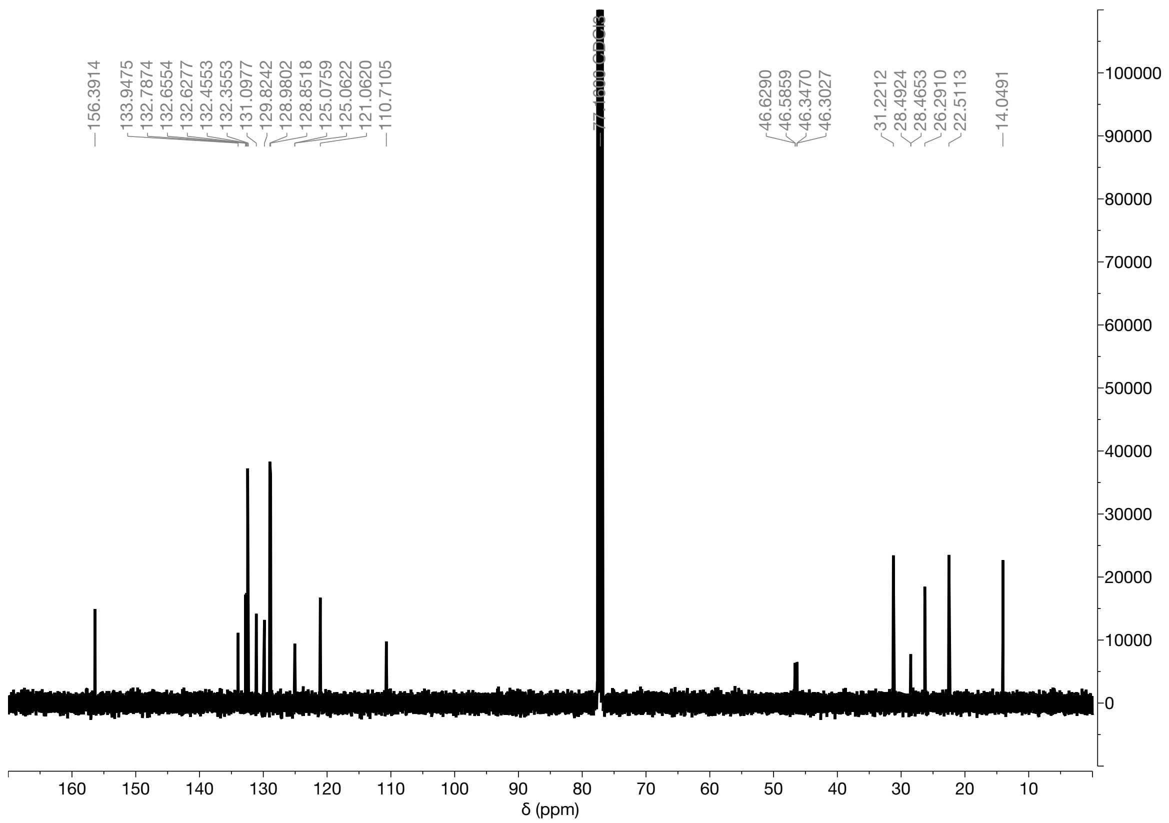

Figure S29. ^{13}C NMR (101 MHz, $CDCl_3$) of compound **5**.

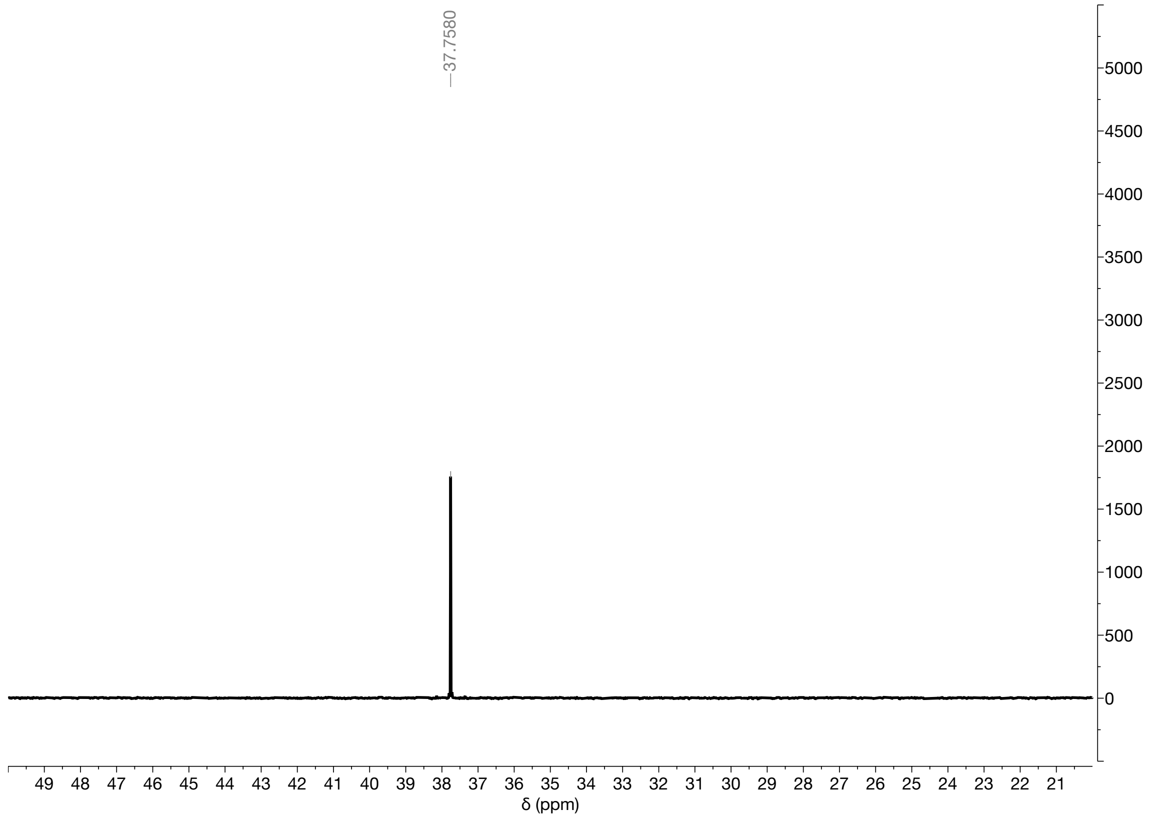

Figure S30. ^{31}P NMR (162 MHz, $CDCl_3$) of compound **5**.

2.6 SYNTHESIS OF 6.

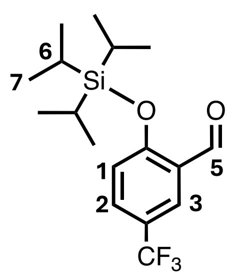

[18] Imidazole (0.73 g, 10.72 mmol) and TIPSCl (1.30 ml, 6.08 mmol) were added to a solution of 2-hydroxy-5-(trifluoromethyl)benzaldehyde (1.00 g, 5.00 mmol) in dimethylformamide (10 ml). The reaction mixture was allowed to stir at room temperature for 18 hours. The solution was then acidified with 0.1M HCl (aq.) and extracted with ethyl acetate. The organic layer was washed with 5% LiCl (aq.), brine and dried over Na_2SO_4 anhydrous. The solution was then concentrated under reduced pressure and the pure product was afforded as a yellow oil by basic alumina flash chromatography using a mixture of petroleum ether and dichloromethane: yield 1.34 g (78%).

^{1}H NMR (400 MHz, $CDCl_3$): δ_H = 10.53 (s, 1H, C<u>H</u>O), 8.09 (d, 1H, J = 2.5 Hz, H_{C3}), 7.68 (dd, 1H, J = 8.7, 2.5 Hz, H_{C2}), 6.99 (d, 1H, J = 8.7 Hz, H_{C1}), 1.38 (m, 3H, H_{C6}), 1.14 (d, 18H, J = 7.5 Hz, H_{C7}).

^{13}C NMR (101 MHz, $CDCl_3$): δ_C = 189.03 (<u>C</u>HO), 161.74 (<u>C</u>-OTIPS), 132.30 (C2), 126.66 (<u>C</u>-C5), 126.31 (d, J = 3.9 Hz,C3), 123.93 (d, J = 271.7 Hz, <u>C</u>-CF_3), 123.78 (q, J = 33.6 Hz, <u>C</u>F_3), 120.36 (C1), 18.03 (C7), 13.12 (C6).

HRMS (ESI+)**:** calculated for $[C_{17}H_{26}F_3O_2Si]^+$ 346.1593, found $[M+H]^+$ 347.1652; calculated for $[C_{17}H_{25}F_3O_2Si]^{2+}$ 173.0797, found $[M]^{2+}$ 173.0795.

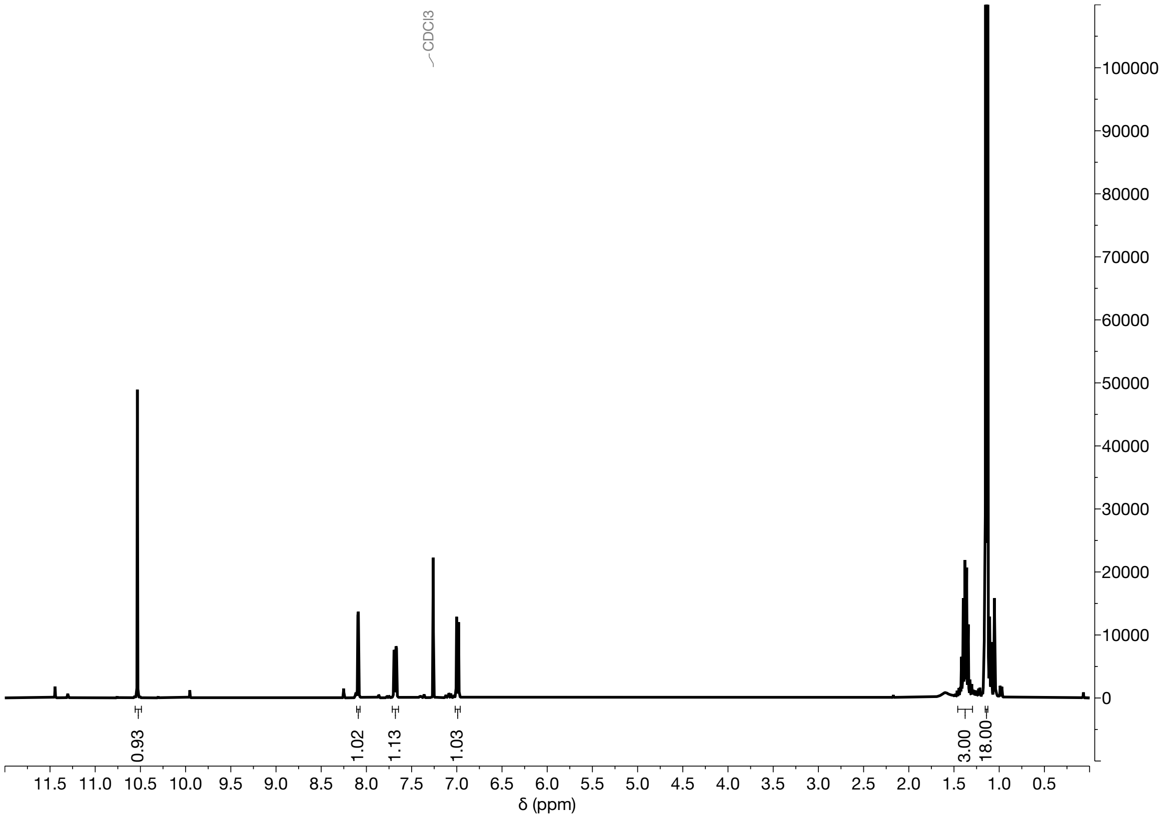

Figure S31. ^{1}H NMR (400 MHz, $CDCl_3$) of compound **18**.

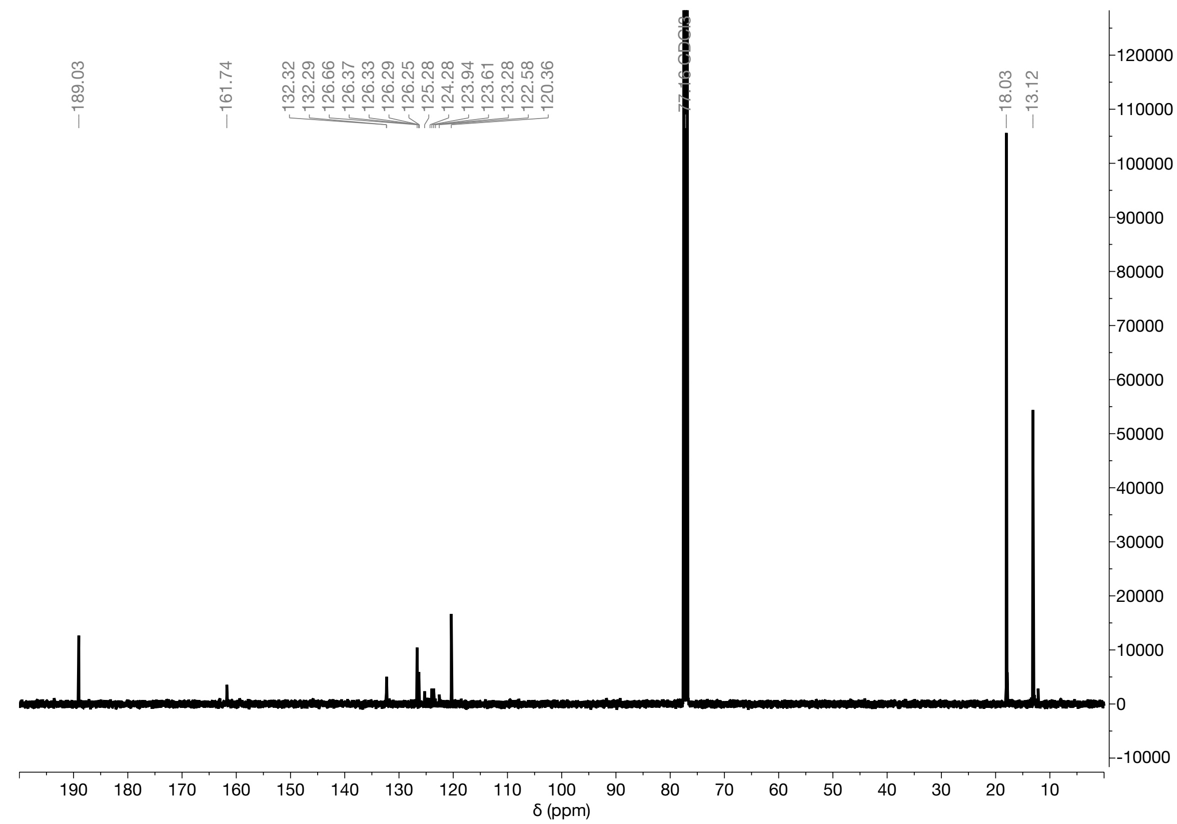

Figure S32. ^{13}C NMR (101 MHz, $CDCl_3$) of compound **18**.

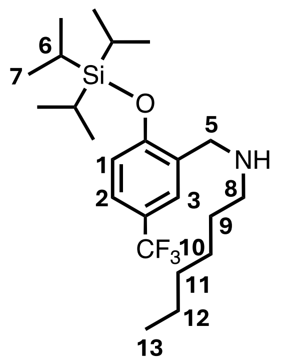

[24] A mixture of **18** (1.03 g, 2.97 mmol) and *n*-hexylamine (0.65 ml, 4.92 mmol) in dry dichloromethane (15 ml) was stirred over molecular sieves at room temperature for 3 hours. The reaction mixture was then cooled to 0 °C and diluted with methanol (15 ml). Sodium borohydride (0.56 g, 14.80 mmol) was added in small aliquots and the reaction was left stirring for 2 hours. The solution was then quenched with water and filtered. After extracting with ethyl acetate, the organic layer was washed with brine and dried over Na_2SO_4 anhydrous. The solution was then concentrated under reduced pressure and the pure product was afforded as a yellow solid by silica flash chromatography using a mixture of dichloromethane and methanol: yield 0.95 g (74%).

^{1}H NMR (400 MHz, $CDCl_3$): δ_H = 7.54 (d, 1H, J = 2.4 Hz, H_{C3}), 7.37 (dd, 1H, J = 8.5, 2.4 Hz, H_{C2}), 6.84 (d, 1H, J = 8.4 Hz, H_{C1}), 3.81 (s, 2H, H_{C5}), 2.60 (t, 2H, J = 7.2 Hz, H_{C8}), 1.50 (p, 2H, J = 7.0 Hz, H_{C9}), 1.32 (m, 9H, $H_{C10\text{-}12}$, H_{C6}), 1.12 (d, 18H, J = 7.4 Hz, H_{C7}), 0.88 (t, 3H, J = 6.3 Hz, H_{C13}).

^{13}C NMR (101 MHz, $CDCl_3$): δ_C = 156.98 (d, J = 1.4 Hz, C-OTIPS), 131.34 (C-C5), 127.05 (d, J = 3.7 Hz, C3), 125.22 (d, J = 3.9 Hz,C2), 124.66 (d, J = 271.3 Hz, C-CF_3), 122.96 (q, J = 32.4 Hz, CF_3), 117.89 (C1), 49.69 (C8), 49.46 (C5), 31.93 (C10), 30.35 (C9), 27.24 (C11), 22.75 (C12), 18.13 (C7), 14.17 (C13), 13.17 (C6).

HRMS (ESI$^+$)**:** calculated for $[C_{23}H_{41}F_3NOSi]^+$ 432.2904, found $[M+H]^+$ 432.2896.

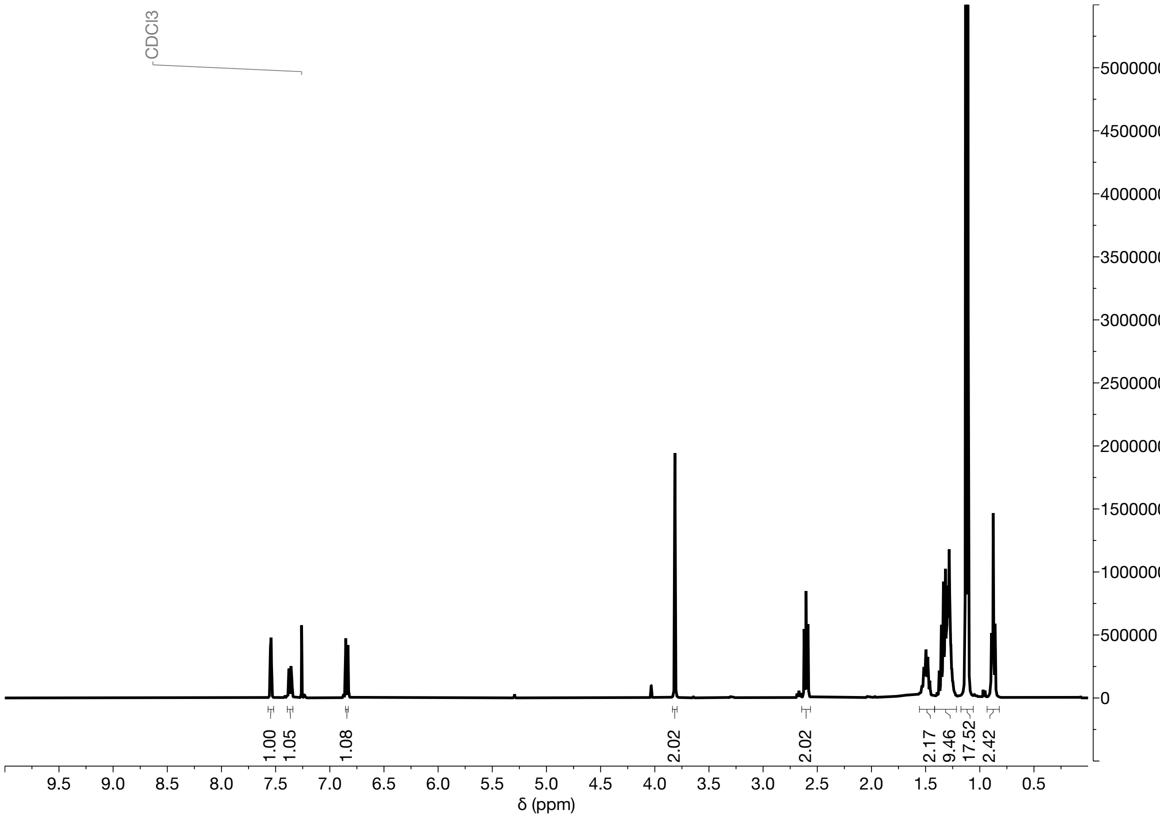

Figure S33. ^{1}H NMR (400 MHz, $CDCl_3$) of compound **24**.

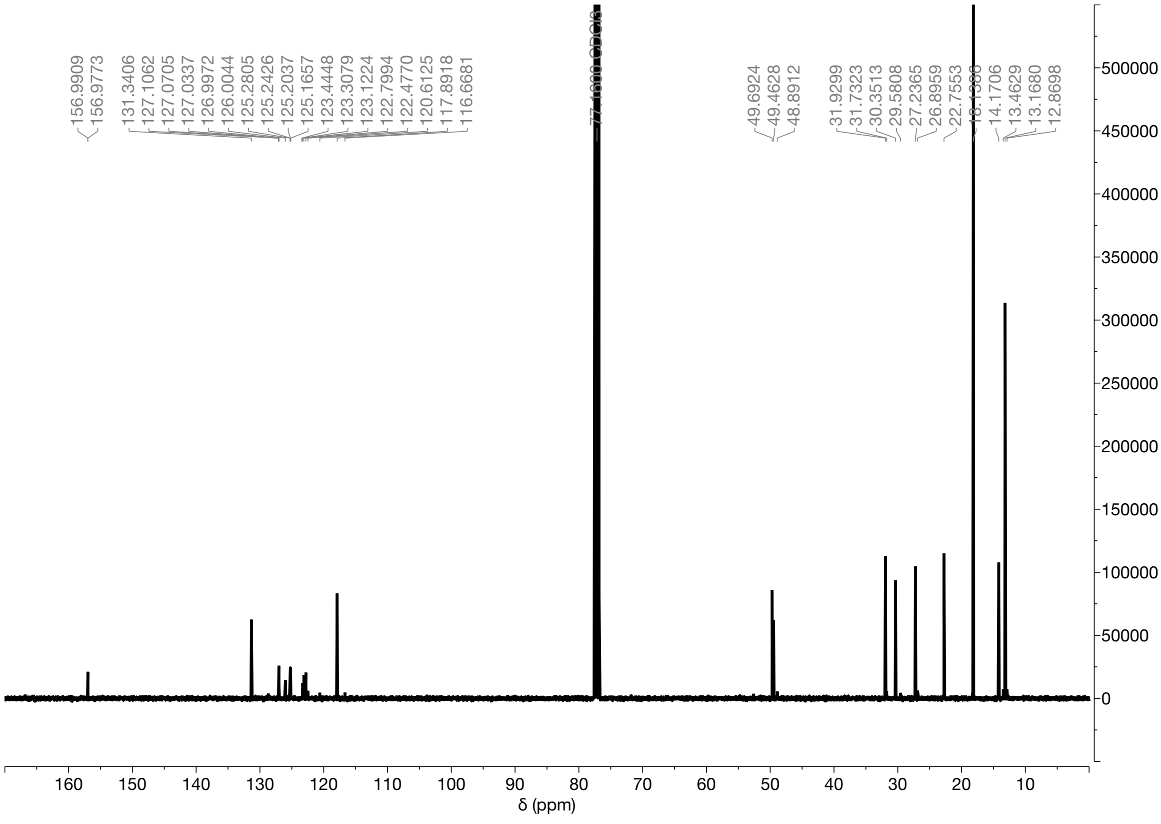

Figure S34. ^{13}C NMR (101 MHz, $CDCl_3$) of compound **24**.

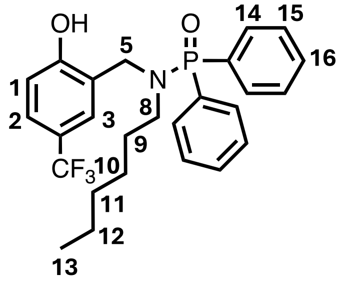

[6] Chlorodiphenylphosphine (0.60 ml, 3.25 mmol) and triethylamine (0.80 ml, 5.74 mmol) were added dropwise to a solution of **24** (0.91 g, 2.10 mmol) in dry tetrahydrofuran (15 ml) at -78°C. The reaction mixture was allowed to stir at room temperature under inert atmosphere for 18 hours. Thereafter, the reaction mixture was cooled to 0 °C and 30% H_2O_2 (aq.) (0.40 ml, 3.91 mmol) was added. The mixture was left stirring for 1 hour before diluting it with iced water. After extracting with dichloromethane, the organic layer was washed with saturated NH_4Cl (aq.) and dried over Na_2SO_4 anhydrous. After concentrating the solution under reduced pressure, 0.20 g of the resulting oil was dissolved in tetrahydrofuran (1 ml) and the solution was cooled to 0 ºC. A 1M solution of TBAF in tetrahydrofuran (0.70 ml, 0.70 mmol) was, then, dropwise added and the mixture was stirred at room temperature for 30 minutes. After adding water (1 ml), the solution was extracted with ethyl acetate. The organic layer was then washed with brine, dried over Na_2SO_4 anhydrous and concentrated under reduced pressure. The final product was purified by preparative HPLC using a XSelect® CSH C18 (5 um, 19 x 150 mm) column with 30% acetonitrile in water: overall yield 0.58 g (58%).

^{1}H NMR (400 MHz, $CDCl_3$)**:** δ_H = 10.95 (s, 1H, O<u>H</u>), 7.76 (m, 4H, H_{C14}), 7.58 (m, 2H, H_{C16}), 7.50 (m, 4H, H_{C15}), 7.47 (m, 1H, H_{C2}), 7.30 (d, 1H, *J* = 2.4 Hz, H_{C3}), 7.03 (d, 1H, *J* = 8.5 Hz, H_{C1}), 4.29 (d, 2H, *J* = 10.1 Hz, H_{C5}), 2.72 (m, 2H, H_{C8}), 1.33 (p, 2H, *J* = 7.3 Hz, H_{C9}), 1.10 (m, 2H, H_{C12}), 0.98 (m, 2H, H_{C11}), 0.91 (m, 2H, H_{C10}), 0.77 (t, 3H, *J* = 7.3 Hz, H_{C13}).

^{13}C NMR (101 MHz, $CDCl_3$)**:** δ_C = 160.23 (<u>C</u>F$_3$), 132.73 (d, *J* = 2.8 Hz, C16), 132.38 (d, *J* = 10.2 Hz, C14), 130.33 (d, *J* = 127.9 Hz, <u>C</u>-P), 128.95 (d, *J* = 12.9 Hz, C15), 127.28 (d, *J* = 3.8 Hz, C2) 124.69 (d, *J* = 270.9 Hz, <u>C</u>-CF$_3$), 123.01 (d, *J* = 1.5 Hz, <u>C</u>-OH), 121.08 (d, *J* = 32.6 Hz, <u>C</u>-C5), 119.29 (C1), 46.99 (d, *J* = 4.4 Hz, C5), 46.65 (d, *J* = 4.5 Hz, C8), 31.15 (C11), 28.57 (d, *J* = 2.7 Hz, C9), 26.24 (C10), 22.45 (C12), 13.94 (C13).

^{31}P NMR (162 MHz, $CDCl_3$): δ_P = 38.35.

HRMS (ESI+)**:** calculated for $[C_{25}H_{30}CF_3NO_2P]^+$ 475.1903, found $[M+H]^+$ 475.1972; calculated for $[C_{25}H_{29}CF_3NO_2PNa]^+$ 498.1903, found $[M+Na]^+$ 498.1794.

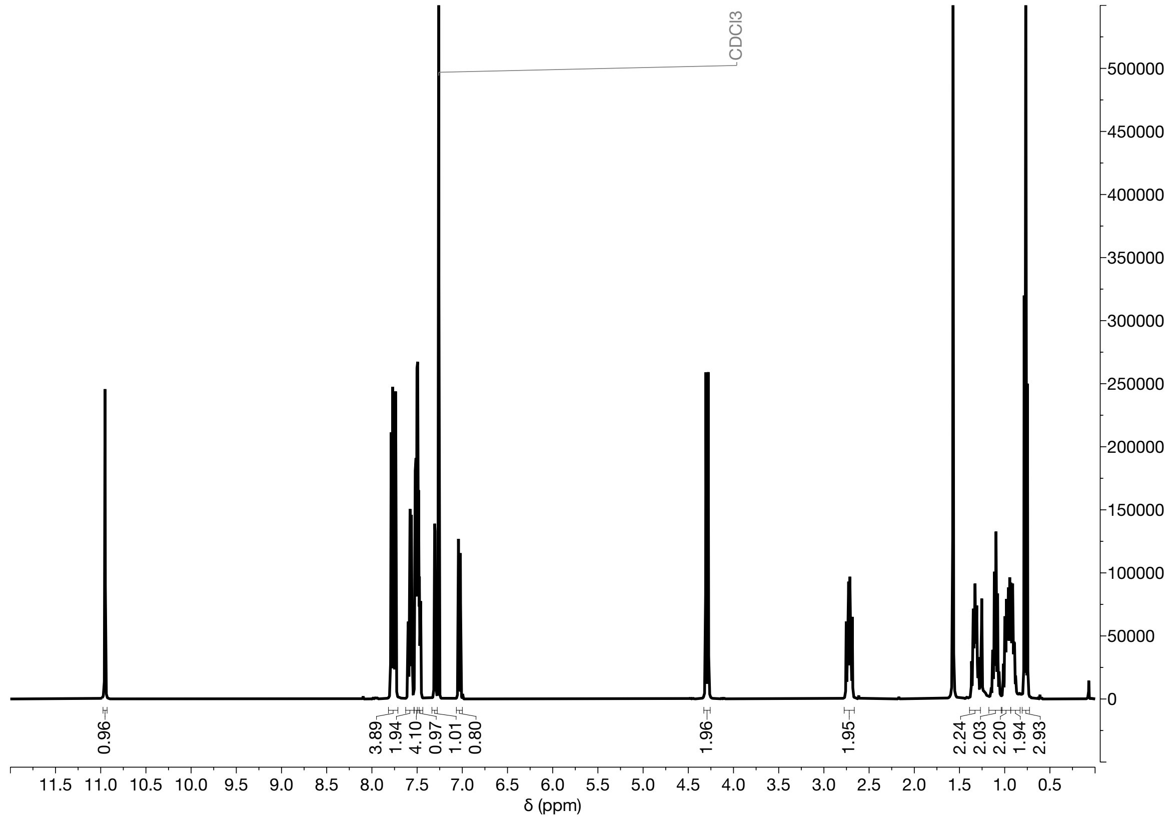

Figure S35. ^{1}H NMR (400 MHz, $CDCl_3$) of compound **6**.

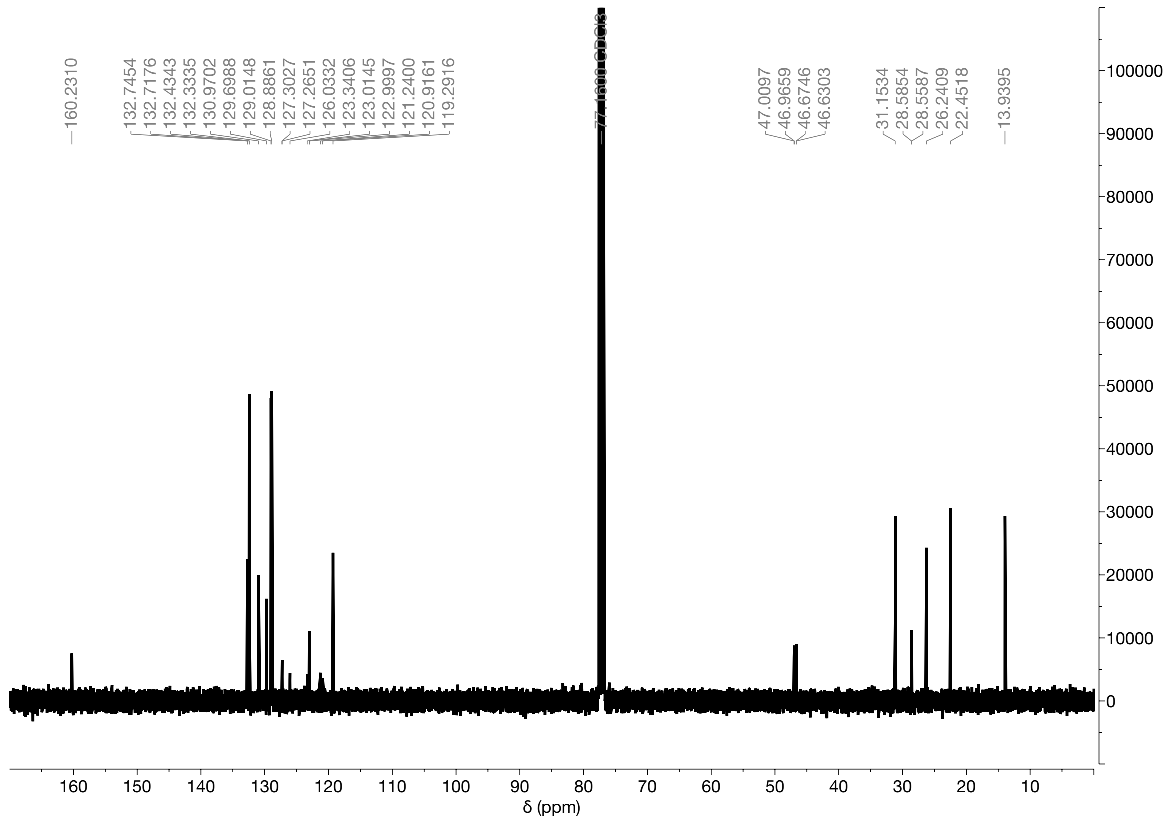

Figure S36. ^{13}C NMR (101 MHz, $CDCl_3$) of compound **6**.

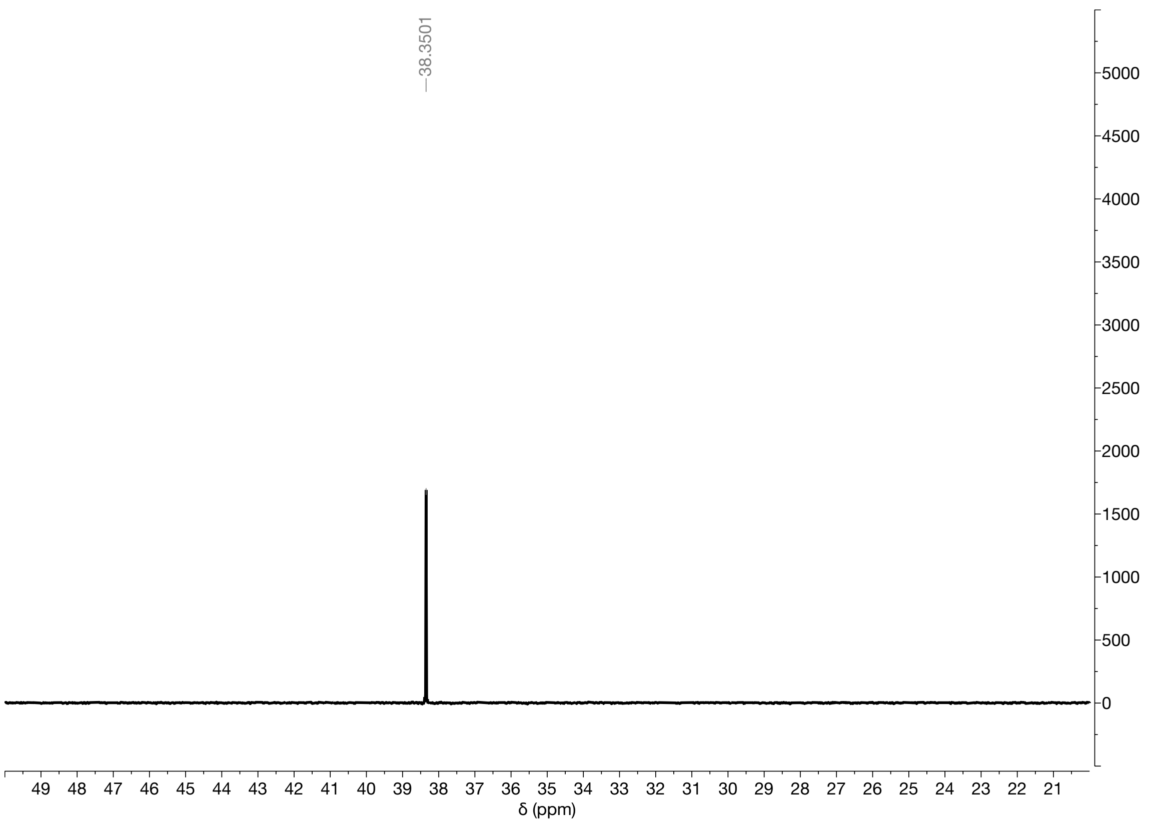

Figure S37. ^{31}P NMR (162 MHz, $CDCl_3$) of compound **6**.

2.7 SYNTHESIS OF 7.

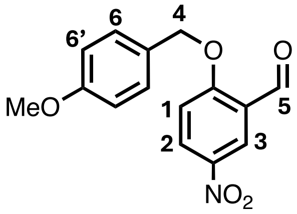

[19] Methoxybenzyl chloride (8.00 ml, 59.00 mmol) was added dropwise to a cooled (0 °C) mixture of 5-hydroxy-2-nitrobenzaldehyde (5.05 g, 30.22 mmol) and K_2CO_3 (8.30 ml, 60.05 mmol) in dimethylformamide (30 ml). The solution was left stirring at room temperature for 18 hours. After adding water, the reaction mixture was extracted with ethyl acetate. The organic layer was washed with and brine, dried over Na_2SO_4 anhydrous and concentrated under reduced pressure. The residue was then purified by recrystallization from ethyl acetate to afford the pure product as a yellow solid: yield 4.21 g (48%).

^{1}H NMR (400 MHz, $CDCl_3$)**:** δ_H = 10.47 (s, 1H, C<u>H</u>O), 8.71 (d, 1H, *J* = 2.9 Hz, H_{C3}), 8.41 (dd, 1H, *J* = 9.2, 2.9 Hz, H_{C2}), 7.37 (d, 2H, *J* = 8.7 Hz, H_{C6}), 7.19 (d, 1H, *J* = 9.2 Hz, H_{C1}), 6.95 (d, 2H, *J* = 8.7 Hz, $H_{C6'}$), 5.25 (s, 2H, H_{C4}), 3.84 (s, 3H, OC<u>H</u>$_3$).

^{13}C NMR (101 MHz, $CDCl_3$)**:** δ_C = 187.69 (<u>C</u>HO), 164.94 (<u>C</u>-OPMB), 160.26 (<u>C</u>-OMe), 141.81 (<u>C</u>-NO_2), 130.67 (C2), 129.51 (C6), 126.59 (<u>C</u>-CHO), 125.06 (C6-<u>C</u>-C4), 124.76 (C3), 114.52 (C6'), 113.63 (C1), 71.63 (C4), 55.50 (O-<u>C</u>H$_3$).

HRMS (ESI$^+$)**:** calculated for $[C_{15}H_{12}NO_5]^-$ 286.0721, found $[M]^-$ 286.0717.

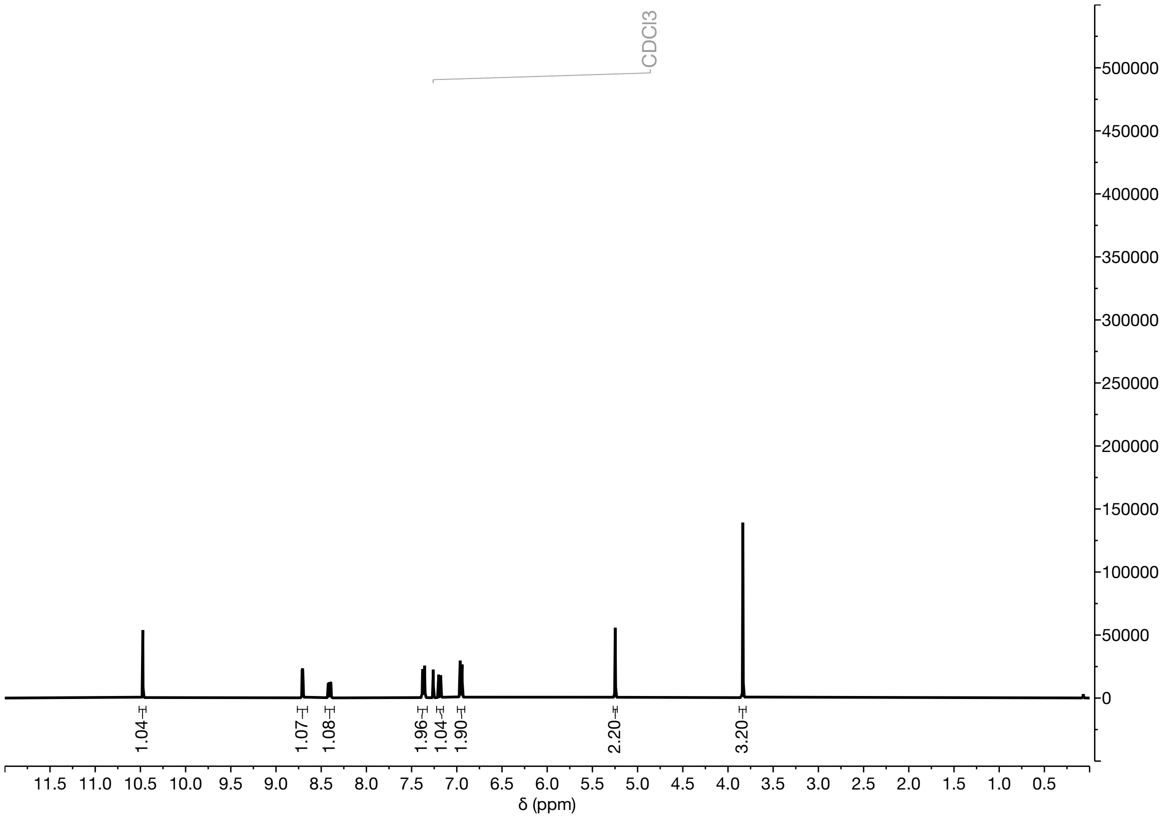

Figure S38. ^{1}H NMR (400 MHz, $CDCl_3$) of compound **19**.

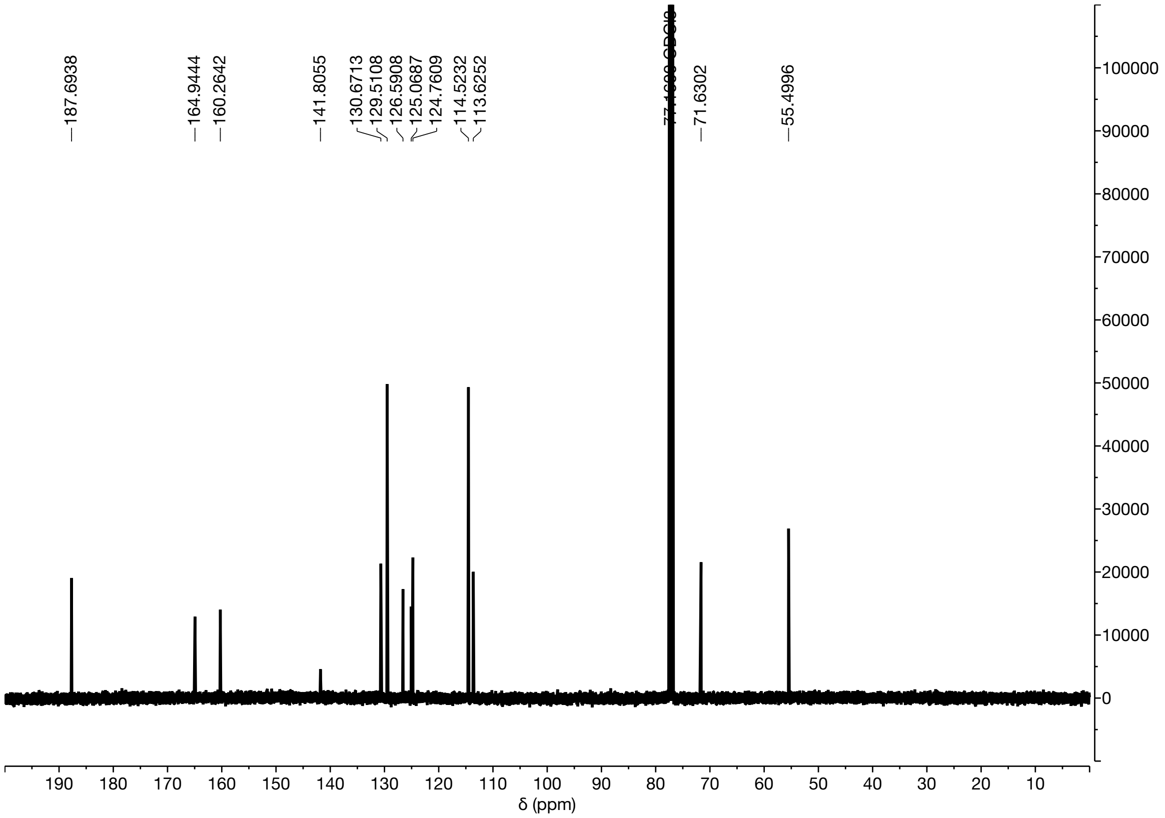

Figure S39. ^{13}C NMR (101 MHz, $CDCl_3$) of compound **19**.

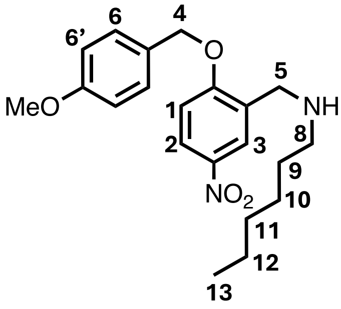

[25] A mixture of **19** (1.12 g, 3.91 mmol) and *n*-hexylamine (0.80 ml, 6.06 mmol) in dry dichloromethane (20 ml) was stirred over molecular sieves at room temperature for 3 hours. The reaction mixture was then cooled to 0 °C and diluted with methanol (20 ml). Sodium borohydride (0.80 g, 21.15 mmol) was added in small aliquots and the reaction was left stirring for 18 hours. The solution was then quenched with water and filtered. After extracting with dichloromethane, the organic layer was washed with brine and dried over Na_2SO_4 anhydrous. The solution was then concentrated under reduced pressure and the pure product was afforded as a yellow oil by silica flash chromatography using a mixture of petroleum ether and ethyl acetate: yield 1.21 g (83%).

^{1}H NMR (400 MHz, $CDCl_3$): δ_H = 8.22 (d, 1H, *J* = 2.8 Hz, H_{C3}), 8.13 (dd, 1H, *J* = 9.0, 2.8 Hz, H_{C2}), 7.33 (d, 2H, *J* = 8.6 Hz, H_{C6}), 6.98 (d, 1H, *J* = 9.0 Hz, H_{C1}), 6.93 (d, 2H, *J* = 8.7 Hz, $H_{C6'}$), 5.12 (s, 2H, H_{C4}), 3.84 (s, 2H, H_{C5}), 3.82 (s, 3H, O-C$\underline{H}_3$), 2.58 (t, 2H, *J* = 7.1 Hz, H_{C8}), 1.46 (p, 2H, *J* = 7.4 Hz, H_{C9}), 1.28 (m, 9H, H_{C10-12}), 0.87 (t, 3H, *J* = 6.8 Hz, H_{C13}).

^{13}C NMR (101 MHz, $CDCl_3$): δ_C = 161.72 ($\underline{C}$-OPMB), 159.91 ($\underline{C}$-OMe), 141.47 ($\underline{C}$-NO_2), 130.63 (C6-$\underline{C}$-C4), 129.18 (C6), 127.70 ($\underline{C}$-C5), 125.09 (C3), 124.54 (C2), 114.31 (C6'), 111.16 (C1), 70.68 (C4), 55.43 (O-$\underline{C}H_3$). 49.59 (C8), 49.74 (C5), 31.89 (C11), 30.24 (C9), 27.12 (C10), 22.74 (C12), 14.18 (C13).

HRMS (ESI$^+$)**:** calculated for $[C_{21}H_{28}N_2O_4Na]^+$ 395.2060, found $[M+H]^+$ 395.1953.

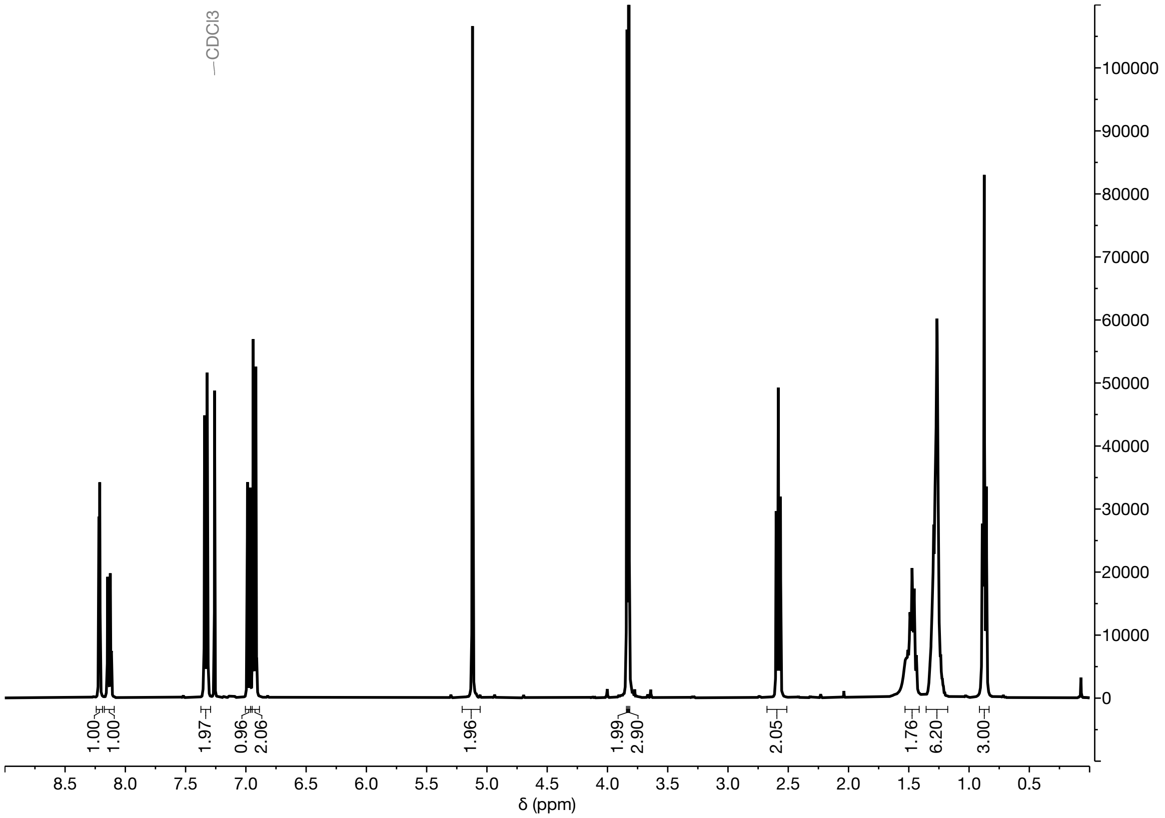

Figure S40. ^{1}H NMR (400 MHz, $CDCl_3$) of compound **25**.

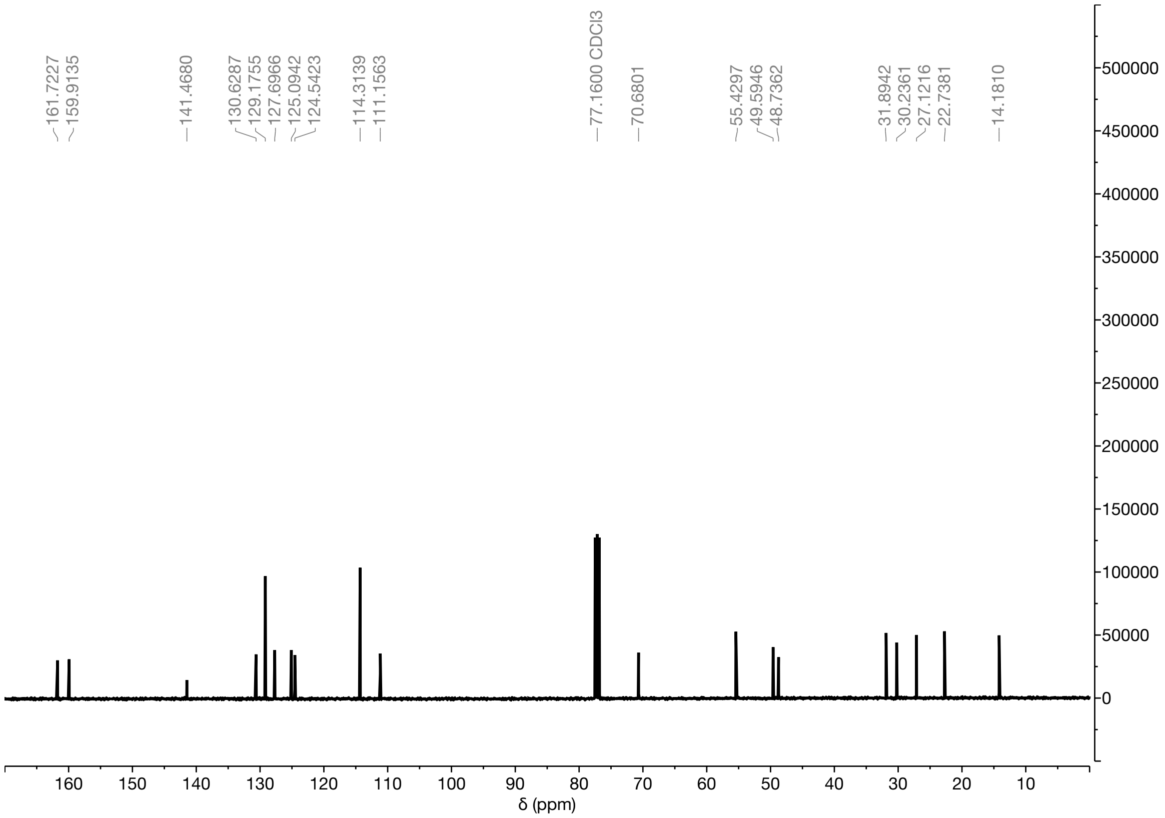

Figure S41. ^{13}C NMR (101 MHz, $CDCl_3$) of compound **25**.

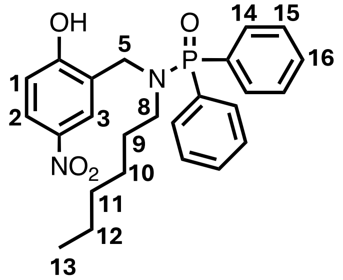

[7] Chlorodiphenylphosphine (0.30 ml, 1.62 mmol) and triethylamine (0.50 ml, 3.59 mmol) were added dropwise to a solution of **25** (0.52 g, 1.40 mmol) in dry tetrahydrofuran (10 ml) at -78°C. The reaction mixture was allowed to stir at room temperature under inert atmosphere for 18 hours. Thereafter, the reaction mixture was cooled to 0 °C and 30% H_2O_2 (aq.) (0.21 ml, 2.09 mmol) was added. The mixture was left stirring for 1 hour before diluting it with iced water. After extracting with dichloromethane, the organic layer was washed with saturated NH_4Cl (aq.) and dried over Na_2SO_4 anhydrous. After concentrating the solution under reduced pressure, 0.02 g of the resulting oil was dissolved in a 1:1 mixture of TFA and dichloromethane (1ml) and the solution was left stirring at room temperature for 15 minutes. After removing TFA by flushing the solution with a stream of nitrogen, the final product was purified by preparative HPLC using a XSelect® CSH C18 (5 um, 19 x 150 mm) column with 55% acetonitrile in water: overall yield 0.28 g (45%).

^{1}H NMR (400 MHz, $CDCl_3$)**:** δ_H = 11.84 (s, 1H, O<u>H</u>), 8.14 (dd, 1H, *J* = 9.1, 2.8 Hz, H_{C2}), 8.03 (d, 1H, *J* = 2.8 Hz, H_{C3}), 7.75 (m, 4H, H_{C14}), 7.60 (m, 2H, H_{C16}), 7.52 (m, 4H, H_{C15}), 7.01 (d, 1H, *J* = 9.1 Hz, H_{C1}), 4.32 (d, 2H, *J* = 10.0 Hz, H_{C5}), 2.75 (m, 2H, H_{C8}), 1.34 (p, 2H, *J* = 7.8 Hz, H_{C9}), 1.09 (m, 2H, H_{C12}), 0.96 (m, 2H, H_{C11}), 0.90 (m, 2H, H_{C10}), 0.76 (t, 3H, *J* = 7.3 Hz, H_{C13}).

^{13}C NMR (101 MHz, $CDCl_3$)**:** δ_C = 163.80 (C-OH), 139.78 (C-NO_2), 132.95 (d, *J* = 3.1 Hz, C16), 132.35 (d, *J* = 10.3 Hz, C14), 129.90 (d, *J* = 127.9 Hz, C-P), 129.05 (d, *J* = 13.0 Hz, C15), 127.79 (C2), 126.36 (C3), 123.10 (C-C5), 119.43 (C1), 47.08 (d, *J* = 4.2 Hz, C5), 46.94 (d, *J* = 4.5 Hz, C8), 31.16 (C11), 28.79 (d, *J* = 2.8 Hz, C9), 26.25 (C10), 22.47 (C12), 13.97 (C13).

^{31}P NMR (162 MHz, $CDCl_3$): δ_P = 39.42.

HRMS (ESI$^+$)**:** calculated for $[C_{25}H_{50}N_2O_4P]^+$ 453.1879, found $[M+H]^+$ 453.1947; calculated for $[C_{25}H_{49}N_2O_4PNa]^+$ 475.1879, found $[M+H]^+$ 475.1771.

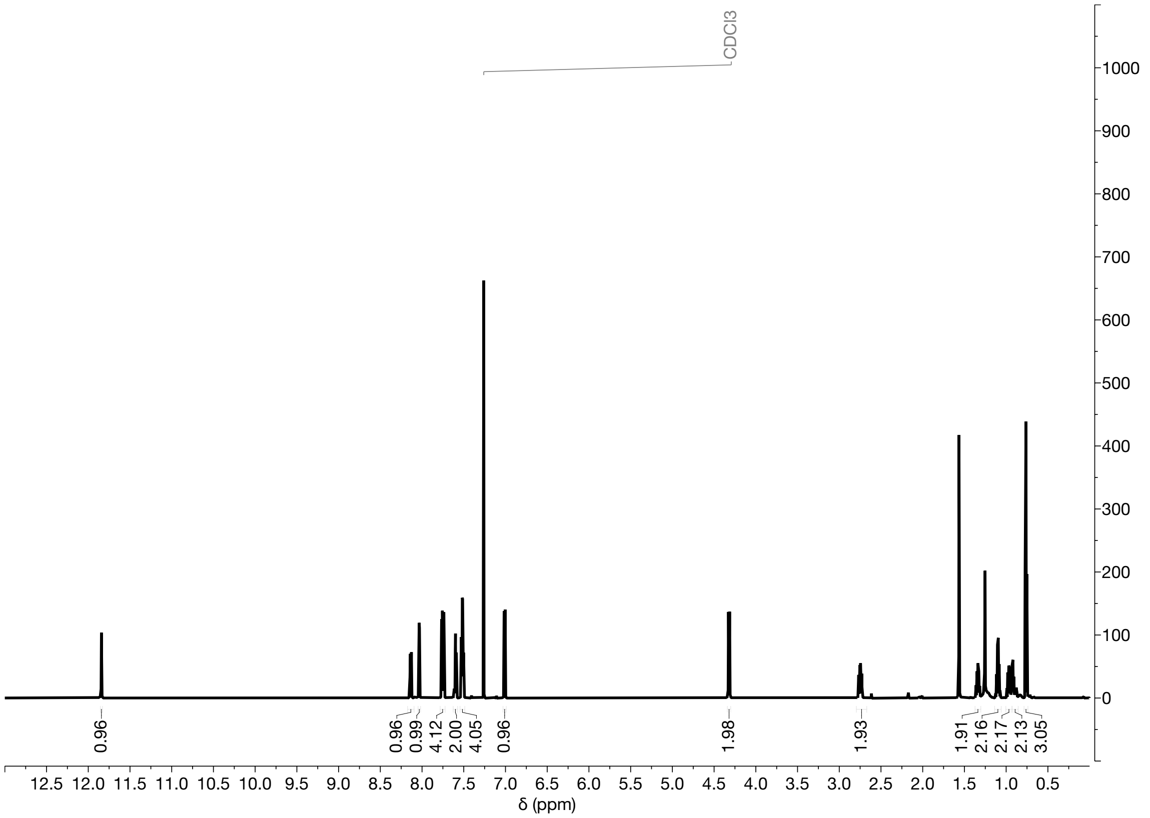

Figure S42. ^{1}H NMR (400 MHz, $CDCl_3$) of compound **7**.

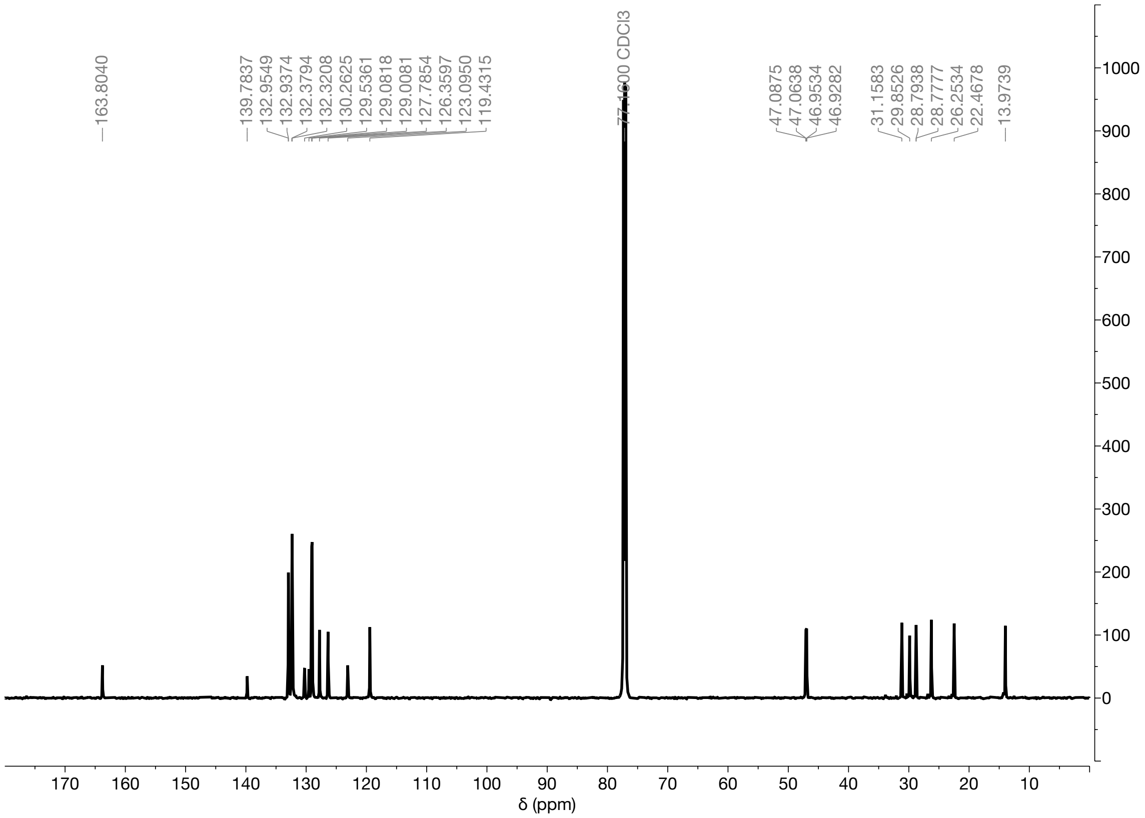

Figure S43. ^{13}C NMR (101 MHz, $CDCl_3$) of compound **7**.

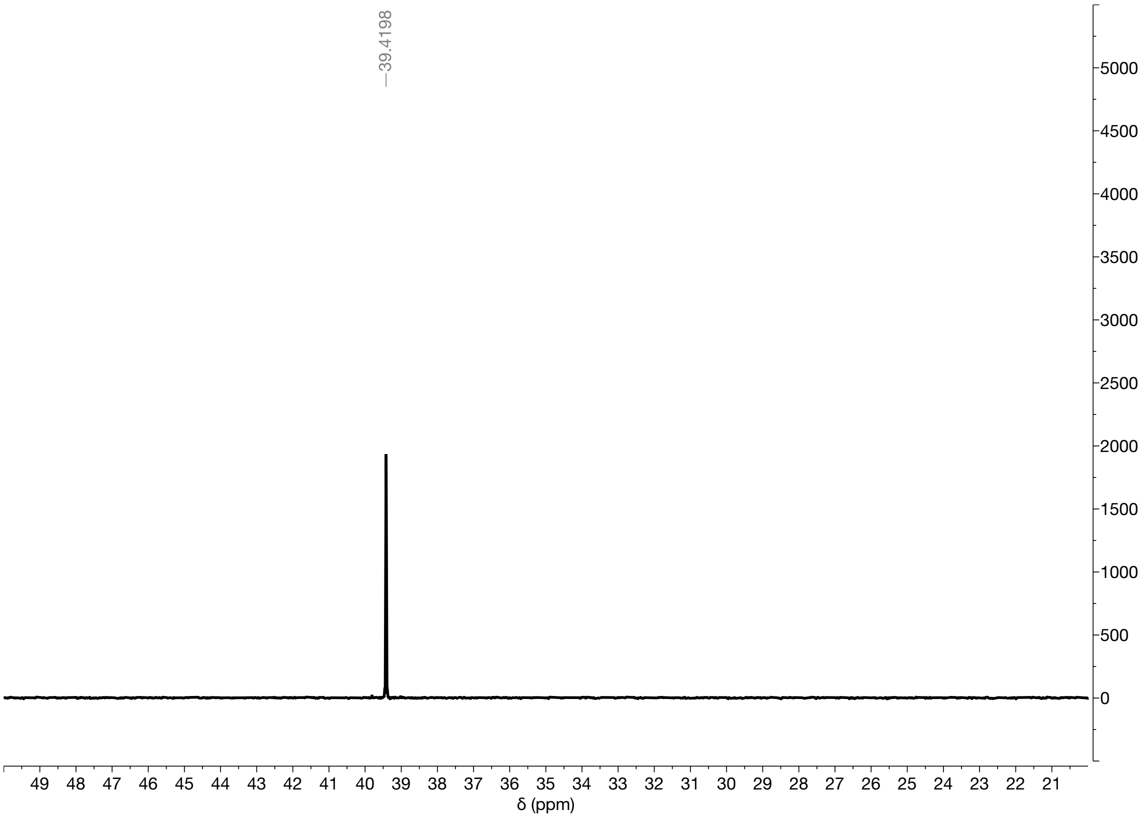

Figure S45. ^{31}P NMR (162 MHz, $CDCl_3$) of compound **7**.

3. NMR TITRATION IN *N*-OCTANE.

NMR titrations were carried out on a Bruker 400 MHz spectrometer using WET solvent suppression. A sample of the host (**1** - **7**) was prepared at a known concentration in *n*-octane. The solution of guest (perfluoro-*t*-butanol, PFTB) was prepared dissolving a known mass of guest in 2 mL of the host solution. A volume of 0.6 mL of the host solution was transferred to an NMR tube and the NMR spectrum of the free host was recorded. Aliquots of the guest solution were successively added to the same NMR tube and the NMR spectrum was recorded after each addition. In case of a dilution experiment, 0.6 mL of pure solvent (*n*-octane) was placed in an NMR tube and aliquots of the compound solution in *n*-octane were added to the same NMR tube, recording the spectrum after each addition. The NMR spectra were then analysed using a purpose-built Python script to fit the changes in the chemical shifts for the different signals to a 1:1 or 1:2 binding isotherms by optimizing the association constant and chemical shift of the free and bound host.

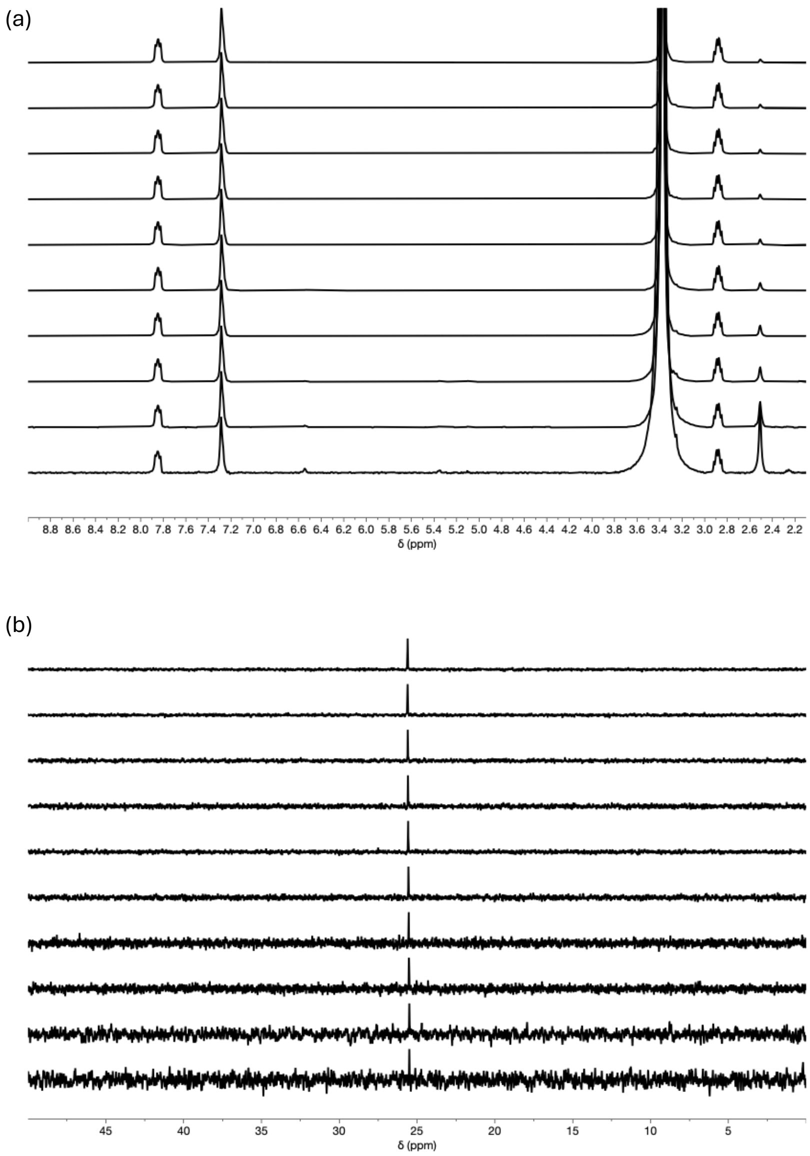

Figure S46. Stack plot for the NMR dilution of **1** in *n*-octane at 298 K, [**1**] ranging from 0.18 mM (bottom) to 4.26 mM (top): (a) ^{1}H NMR; (b) ^{31}P NMR.

(a)

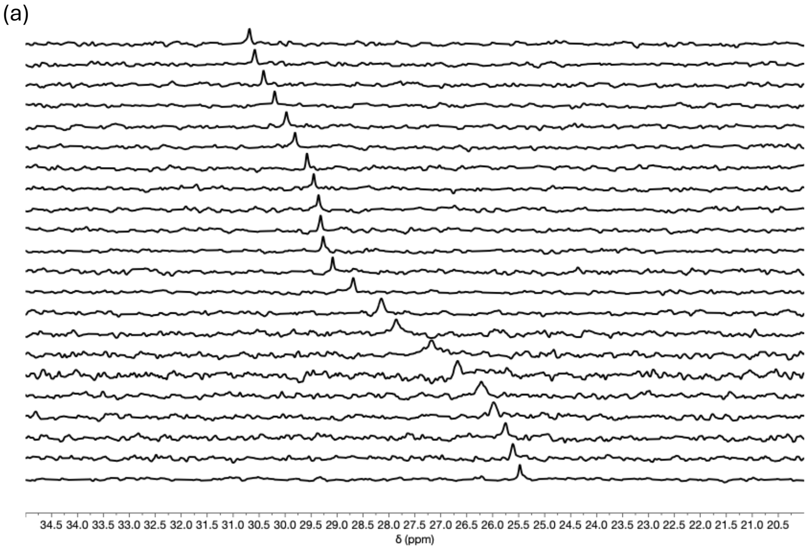

(b)

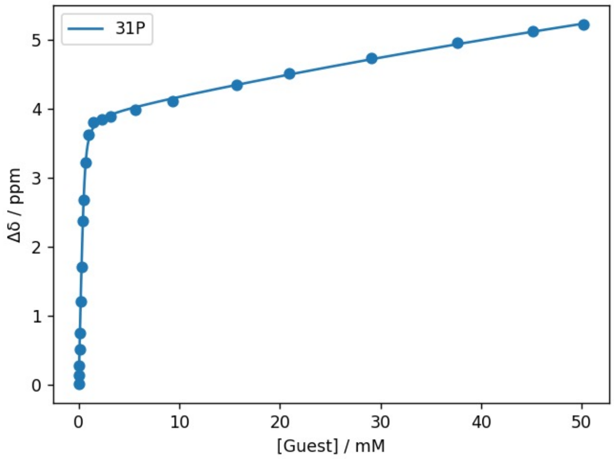

Figure S47. ^{31}P NMR titration of PFTB into **1** (0.55 mM) in *n*-octane at 298 K: (a) stack plot of the NMR spectra, [PFTB] ranging from 0 mM (bottom) to 50.20 mM (top); (b) best fit of the experimental data to a 1:2 binding isotherm.

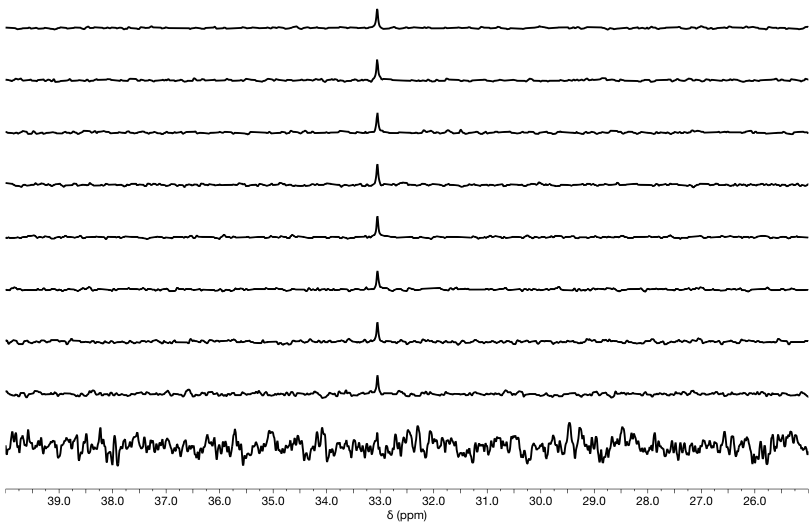

Figure S48. Stack plot for the NMR dilution of **2** in *n*-octane at 298 K, [**2**] ranging from 0.04 mM (bottom) to 1.72 mM (top).

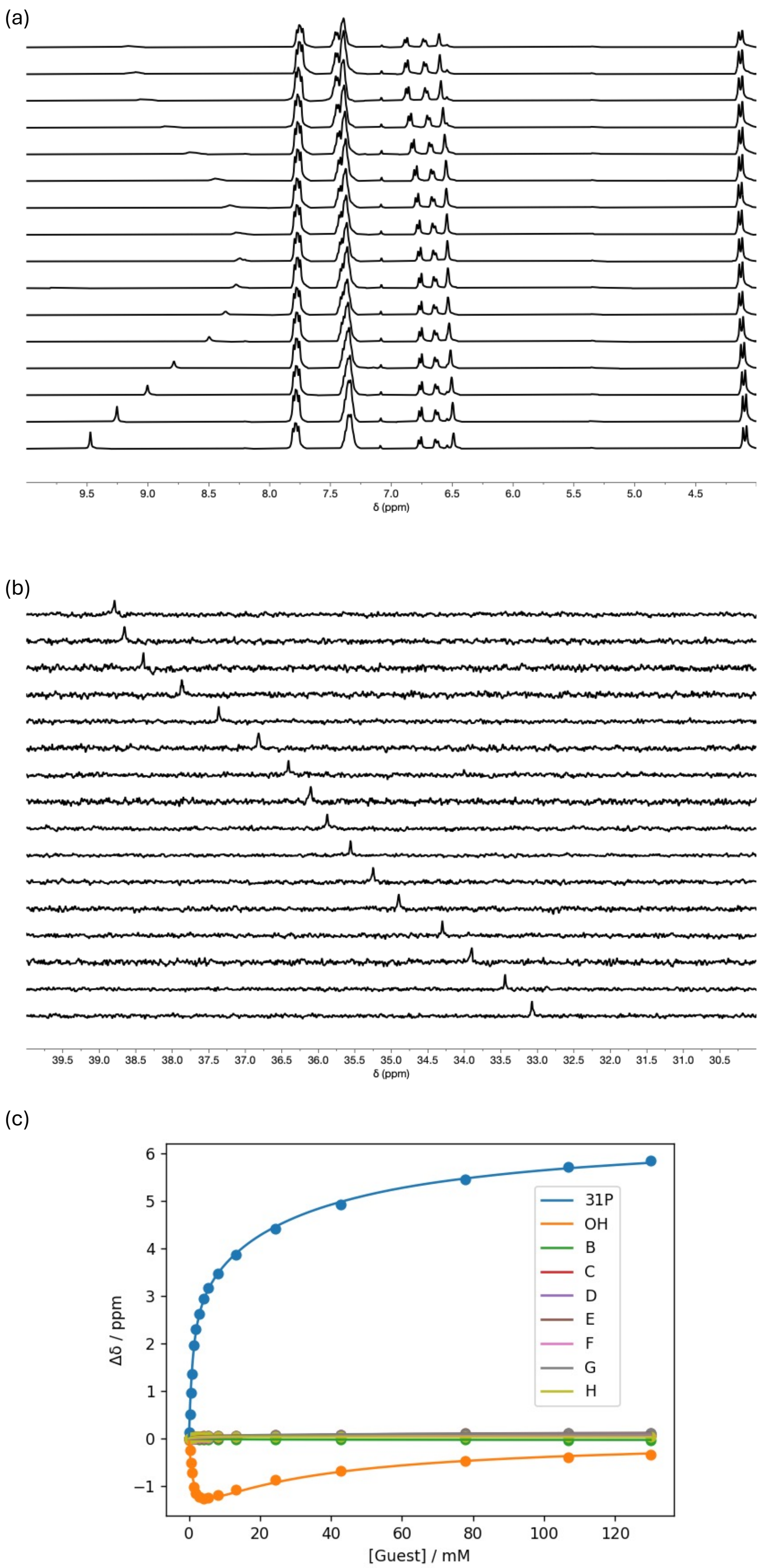

Figure S49. NMR titration of PFTB into **2** (0.50 mM) in *n*-octane at 298 K: (a) stack plot of the ^{1}H NMR spectra, [PFTB] ranging from 0 mM (bottom) to 130.04 mM (top); (b) stack plot of the ^{31}P NMR spectra, [PFTB] ranging from 0 mM (bottom) to 130.04 mM (top); (c) best fit of the experimental data to a 1:2 binding isotherm.

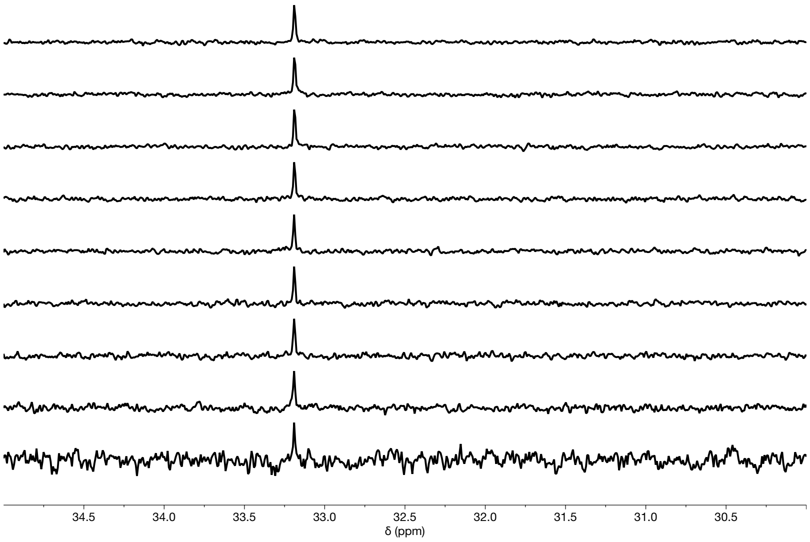

Figure S50. Stack plot for the NMR dilution of **3** in *n*-octane at 298 K, [**3**] ranging from 0.07 mM (bottom) to 1.78 mM (top).

(a)

(b)

(c)

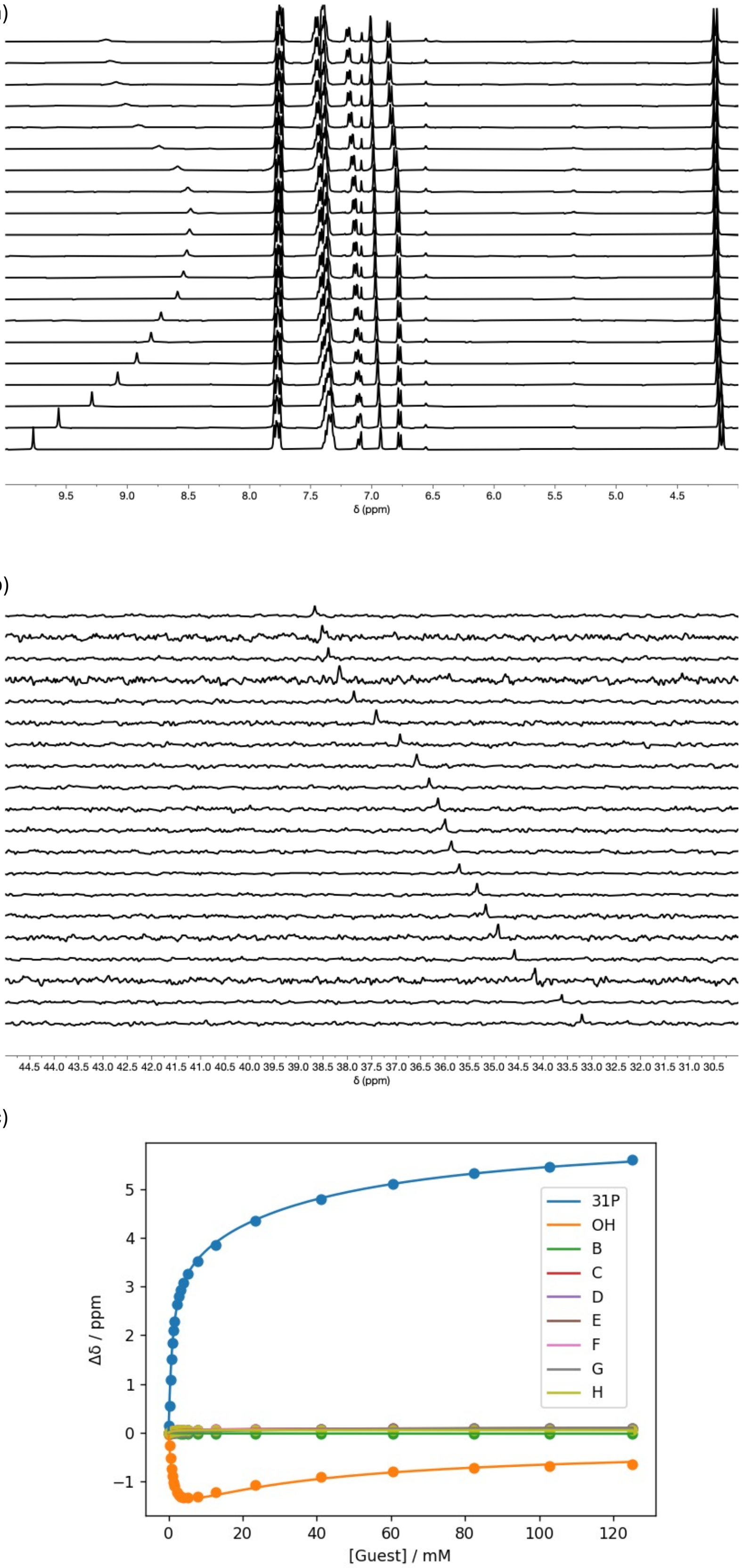

Figure S51. NMR titration of PFTB into **3** (0.50 mM) in *n*-octane at 298 K: (a) stack plot of the ^{1}H NMR spectra, [PFTB] ranging from 0 mM (bottom) to 125.10 mM (top); (b) stack plot of the ^{31}P NMR spectra, [PFTB] ranging from 0 mM (bottom) to 125.10 mM (top); (c) best fit of the experimental data to a 1:2 binding isotherm.

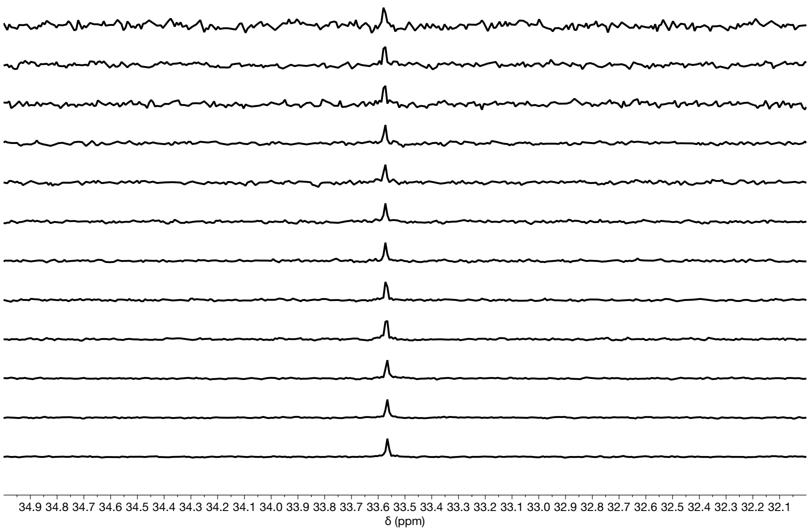

Figure S52. Stack plot for the NMR dilution of **4** in *n*-octane at 298 K, [**4**] ranging from 0.06 mM (bottom) to 2.85 mM (top).

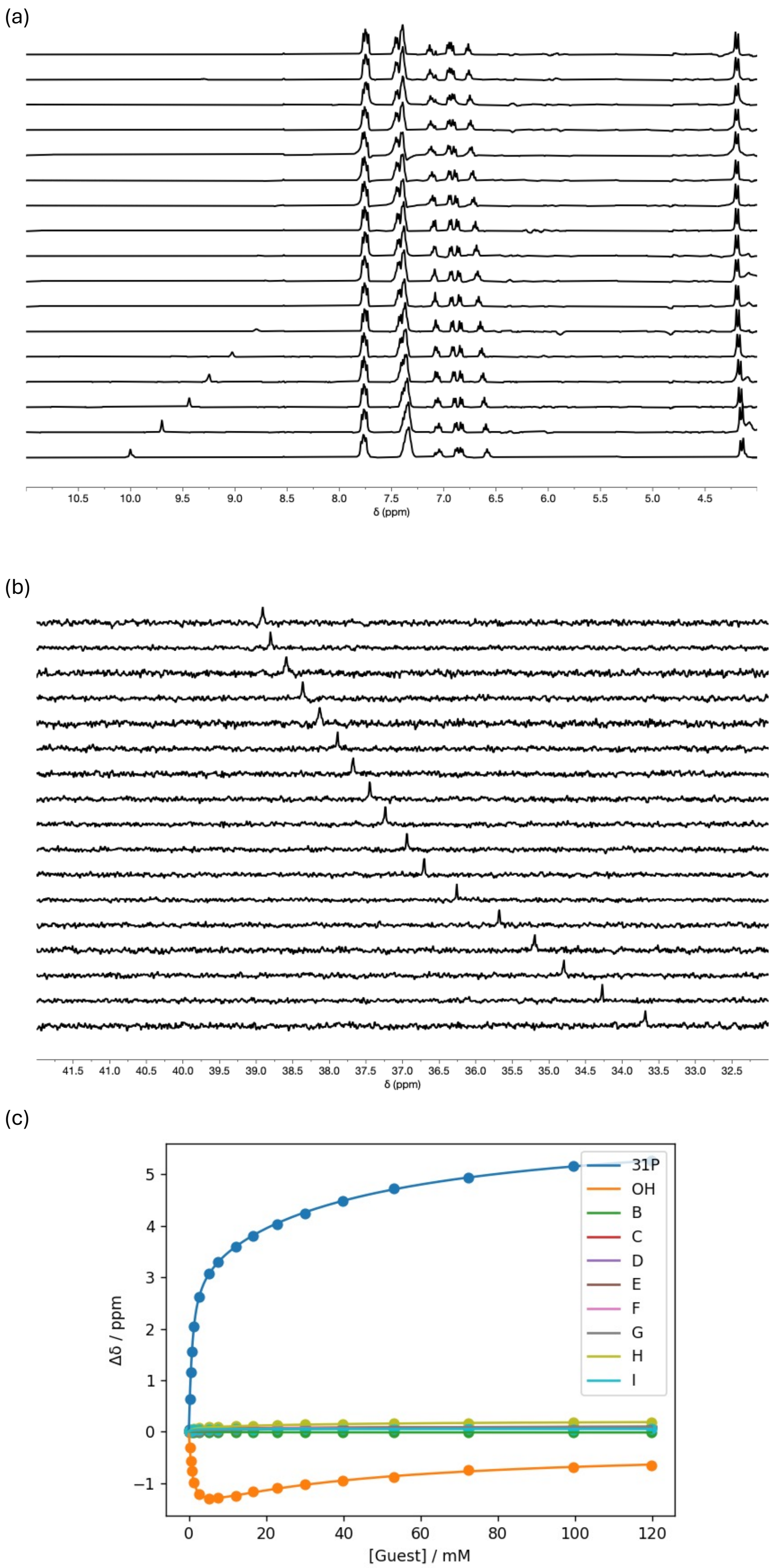

Figure S53. NMR titration of PFTB into **4** (0.48 mM) in *n*-octane at 298 K: (a) stack plot of the ^{1}H NMR spectra, [PFTB] ranging from 0 mM (bottom) to 119.80 mM (top); (b) stack plot of the ^{31}P NMR spectra, [PFTB] ranging from 0 mM (bottom) to 119.80 mM (top); (c) best fit of the experimental data to a 1:2 binding isotherm.

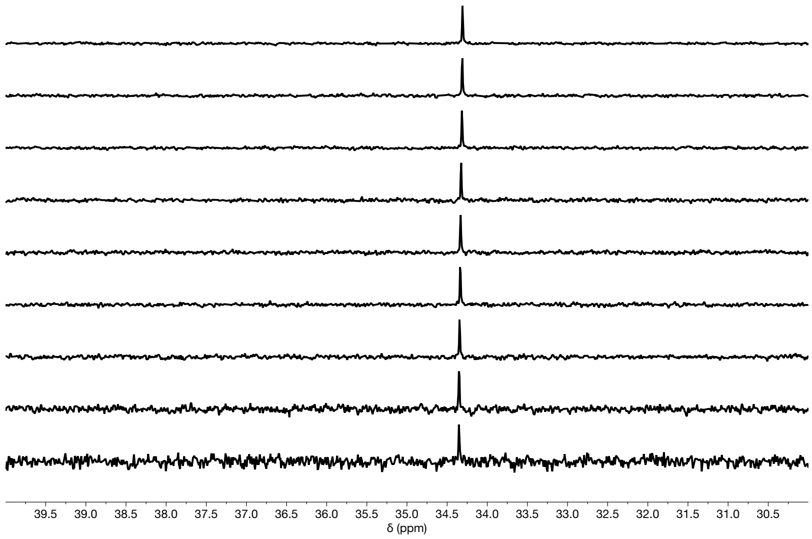

Figure S54. Stack plot for the NMR dilution of **5** in *n*-octane at 298 K, [**5**] ranging from 0.08 mM (bottom) to 1.92 mM (top).

(a)

(b)

(c)

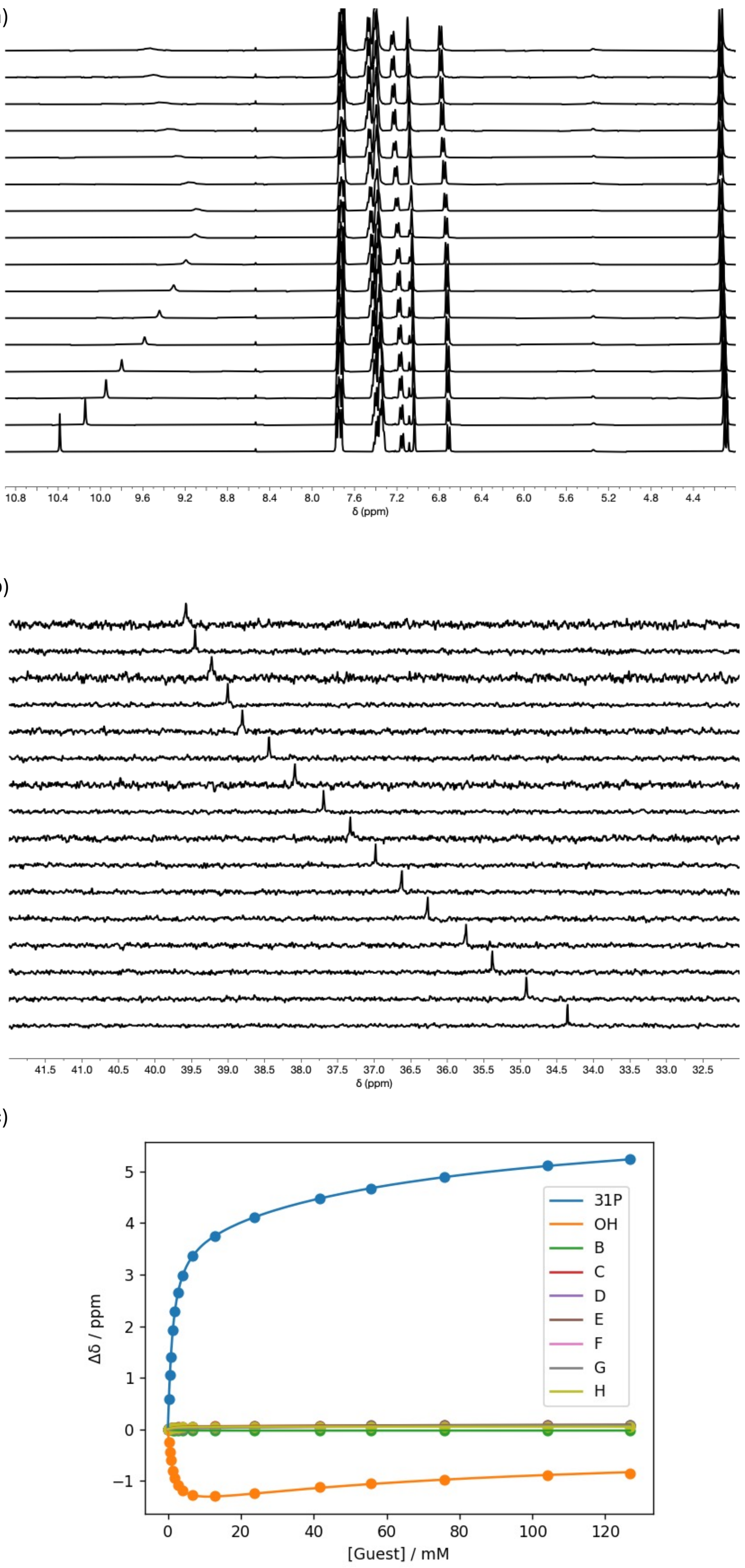

Figure S55. NMR titration of PFTB into **5** (0.50 mM) in *n*-octane at 298 K: (a) stack plot of the ^{1}H NMR spectra, [PFTB] ranging from 0 mM (bottom) to 126.77 mM (top); (b) stack plot of the ^{31}P NMR spectra, [PFTB] ranging from 0 mM (bottom) to 126.77 mM (top); (c) best fit of the experimental data to a 1:2 binding isotherm.

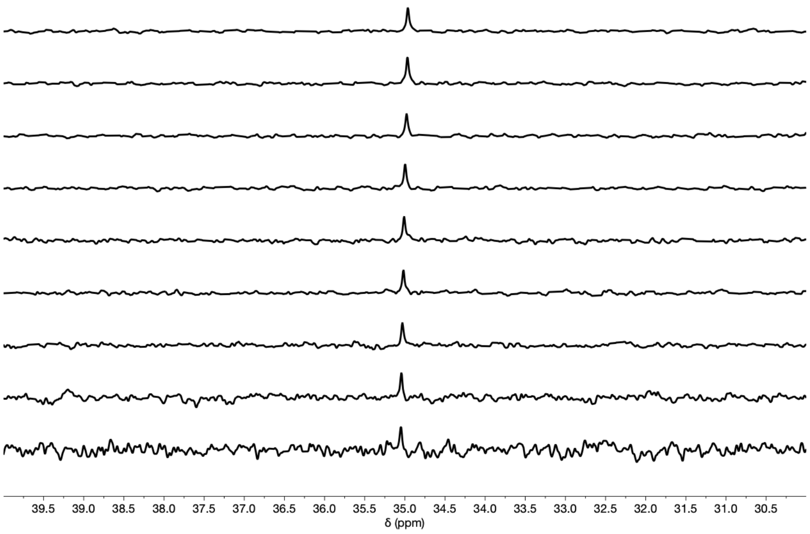

Figure S56. Stack plot for the NMR dilution of **6** in *n*-octane at 298 K, [**6**] ranging from 0.06 mM (bottom) to 1.47 mM (top).

(a)

(b)

(c)

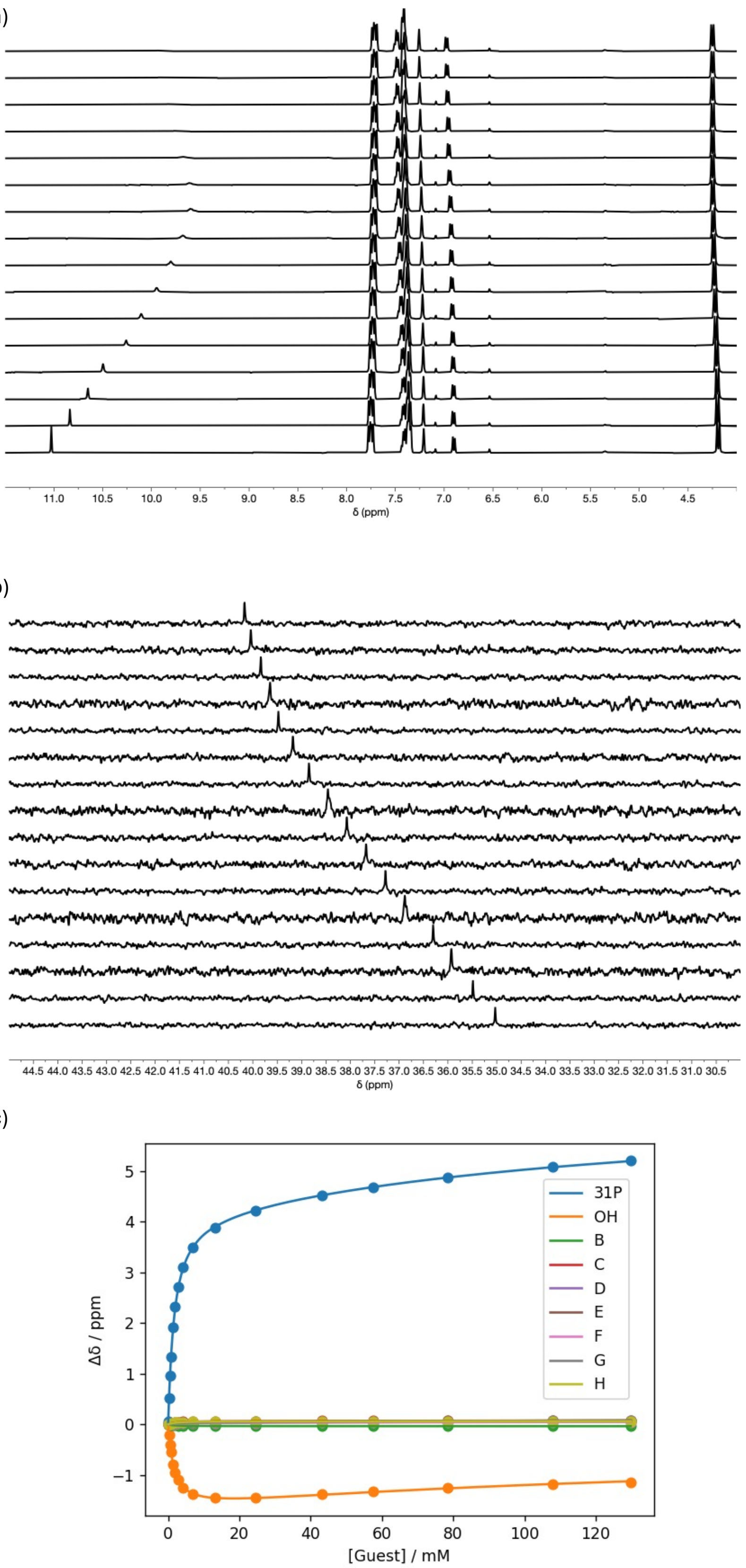

Figure S57. NMR titration of PFTB into **6** (0.50 mM) in *n*-octane at 298 K: (a) stack plot of the ^{1}H NMR spectra, [PFTB] ranging from 0 mM (bottom) to 129.81 mM (top); (b) stack plot of the ^{31}P NMR spectra, [PFTB] ranging from 0 mM (bottom) to 129.81 mM (top); (c) best fit of the experimental data to a 1:2 binding isotherm.

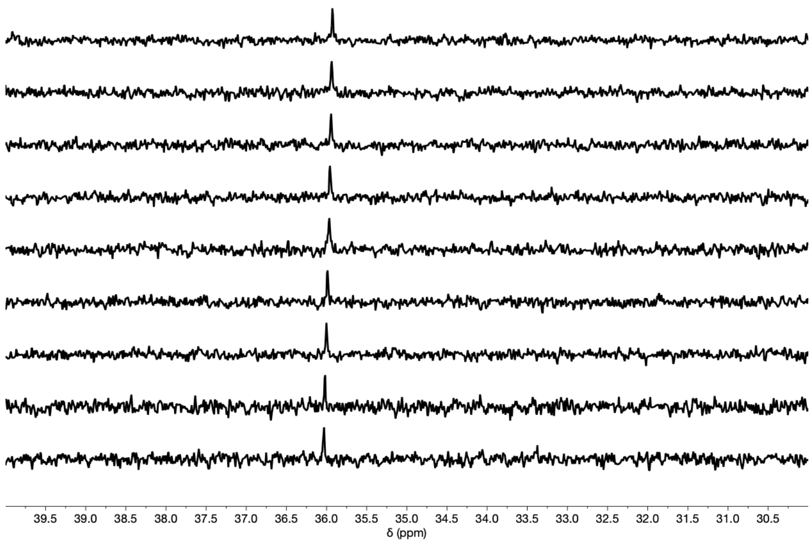

Figure S58. Stack plot for the NMR dilution of **7** in *n*-octane at 298 K, [**7**] ranging from 0.09 mM (bottom) to 0.86 mM (top).

(a)

(b)

(c)

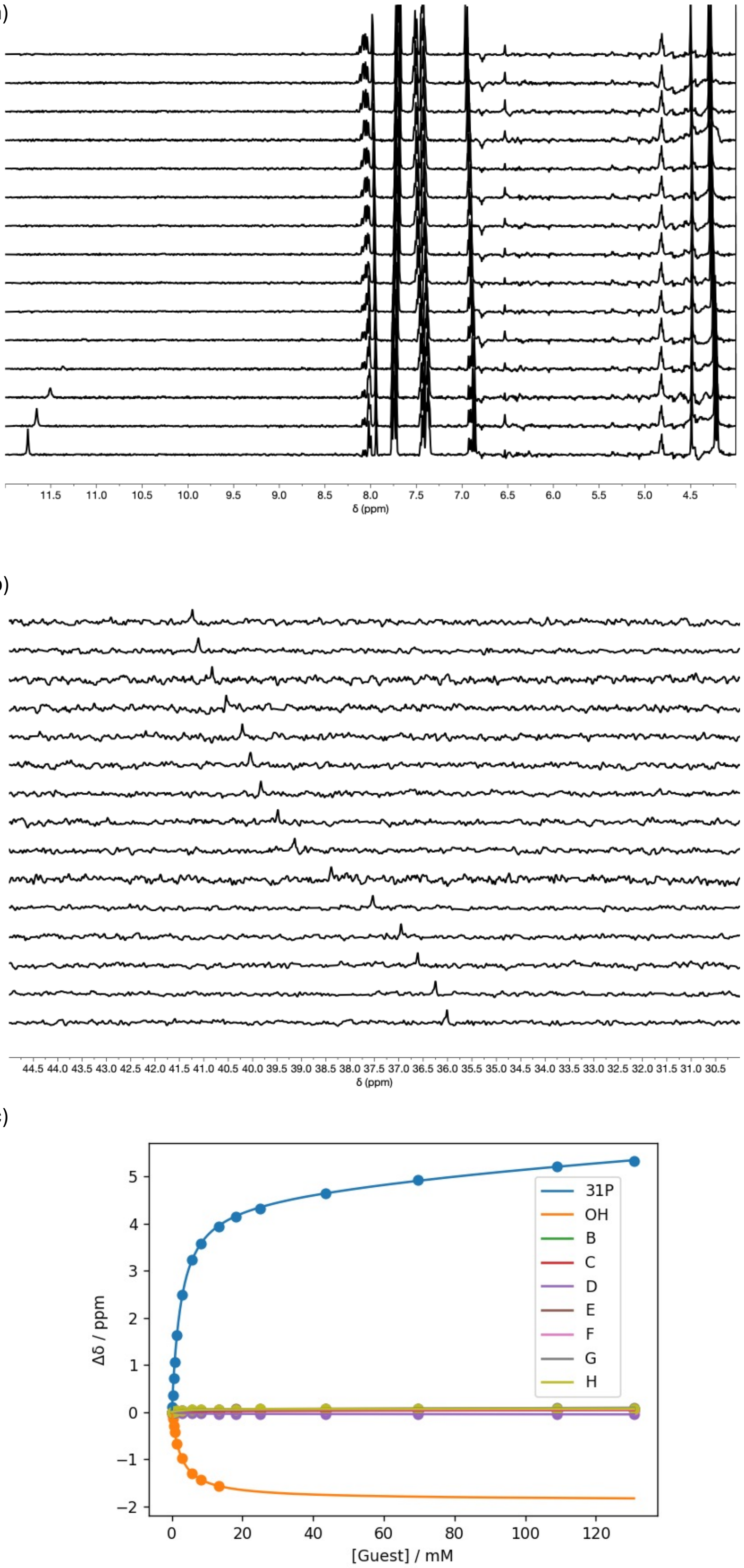

Figure S59. NMR titration of PFTB into **7** (0.56 mM) in *n*-octane at 298 K: (a) stack plot of the ^{1}H NMR spectra, [PFTB] ranging from 0 mM (bottom) to 130.80 mM (top); (b) stack plot of the ^{31}P NMR spectra, [PFTB] ranging from 0 mM (bottom) to 130.80 mM (top); (c) best fit of the experimental data to a 1:2 binding isotherm.

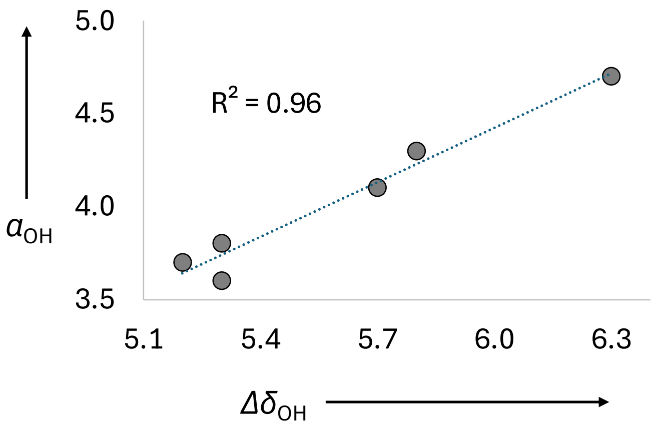

Figure S60. Relationship between the H-bond donor parameter (α) of reference phenols **8**-**13** and the chemical shift difference for the OH signal (**$\Delta\delta_{OH}$**) between compounds **2**-**7** and the corresponding phenol **8**-**13.** The best fit line is y = 0.98x - 1.44 (R^2 = 0.96).

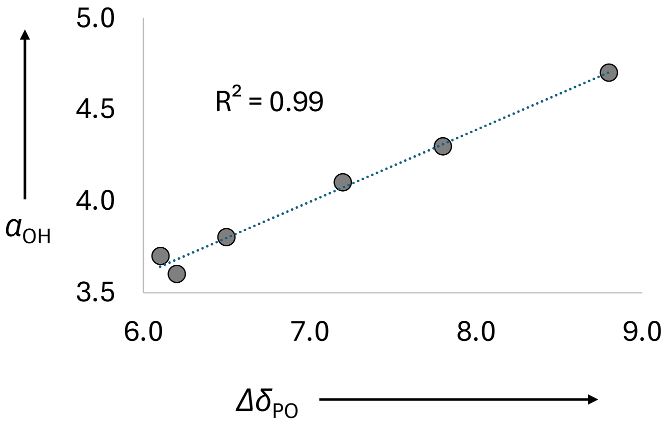

Figure S61. Relationship between the H-bond donor parameter (α) of reference phenols **8**-**13** and the chemical shift difference for the PO signal (**$\Delta\delta_{OH}$**) between compounds **2**-**7** and the corresponding phosphinamide **1.** The best fit line is y = 0.39x + 1.24 (R^2 = 0.99).

4. UV-VIS TITRATION IN *N*-OCTANE.

UV-Vis titrations were carried out on an Agilent Cary 60 UV-Vis spectrophotometer using standard titration protocols. A sample of the host (**1**) was prepared at a known concentration in *n*-octane. The solution of guest (perfluoro-*t*-butanol, PFTB) was prepared dissolving a known mass of guest in 1 mL of the host solution. A volume of 2 mL of the host solution was transferred to a quartz cuvette and the absorption spectrum of the free host was recorded. Aliquots of the guest solution were successively added to the same cuvette and the absorption spectrum was recorded after each addition. The UV-Vis spectra were then analysed using a purpose-built Python script to fit the changes in the absorption at fixed wavelengths to a 1:1 or 1:2 binding isotherms by optimizing the association constant and absorption of the free and bound host.

(a)

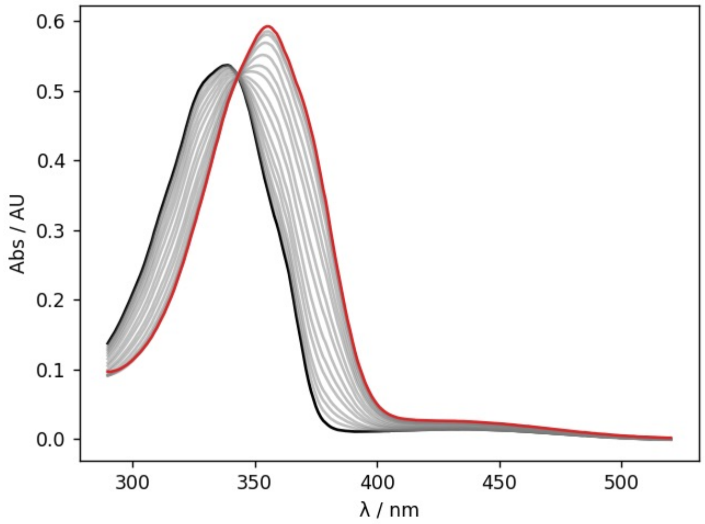

(b)

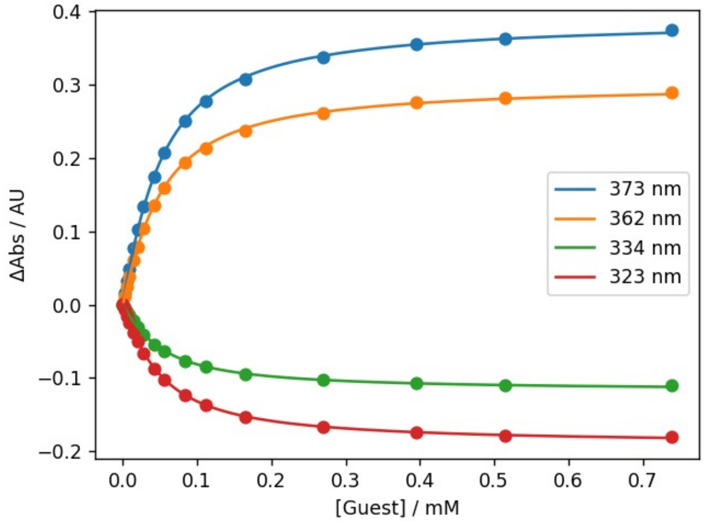

Figure S62. UV-Vis titration of 4-phenyl azophenol into **1** (0.03 mM) in *n*-octane at 298 K: (a) UV-Vis absorption overlapped spectra, [4-phenyl azophenol] ranging from 0 mM (black) to 0.74 mM (red); (b) best fit of the experimental data to a 1:1 binding isotherm.
